# Proceedings of the Canadian Society of Allergy and Clinical Immunology Annual Scientific Meeting 2018

**DOI:** 10.1186/s13223-019-0322-9

**Published:** 2019-03-05

## Allergic Rhinitis/Asthma

### A1 Gene and environmental interactions associated with recurrent wheeze in the Canadian Healthy Infant Longitudinal Development study

#### Jihoon Choi^1^, Christopher Dharma^3^, Amel Lamri^3^, Amirthagowri Ambalavanan^1^, Diana Lefebvre^3^, Stuart Turvey^4^, Piush Mandhane^5^, Allan Becker^6^, Meghan Azad^6^, Theo Moraes^7,8^, Malcom Sears^9,10^, Padmaja Subbarao^7,8,9^, Qingling Duan^1,2^

##### ^1^Department of Biomedical and Molecular Sciences, Queen’s University, Kingston, Ontario, Canada; ^2^School of Computing, Queen’s University, Kingston, Ontario, Canada; ^3^Department of Clinical Epidemiology and Biostatistics, McMaster University, Hamilton, Ontario, Canada; ^4^Division of Allergy and Immunology, Department of Pediatrics, University of British Columbia, BC, Canada; ^5^Division of Pediatric Respiratory Medicine, University of Alberta, Alberta, Canada; ^6^Department of Pediatrics and Child Health, University of Manitoba, Manitoba, Canada; ^7^The Hospital for Sick Children, Toronto, Ontario, Canada; ^8^Department of Paediatrics, University of Toronto, Toronto, Ontario, Canada; ^9^Division of Respirology, Department of Medicine, McMaster University, Hamilton, Ontario, Canada; ^10^Department of Clinical Epidemiology and Biostatistics, McMaster University, Hamilton, Ontario, Canada

**Correspondence:** Jihoon Choi

*Allergy, Asthma and Clinical Immunology* 2019, **15(Suppl 1):**A1

**Background:** Earlier studies have evaluated that genetics contribute to 55–74% of asthma heritability, of which only small percentage may be explained by known loci [1]. We hypothesize that the missing heritability lies within interactions among genes as well as between genes with environmental exposures. Using genomics data from the Canadian Healthy Infant Longitudinal Development study (CHILD; N = 3455) [2], we hereby investigate the effects of common and rare variants as well as their interactions with modifiable exposures on risk of recurrent wheeze during early childhood.

**Methods:** We ascertained genomics data using the Illumina HumanCoreExome BeadChip. After quality control assessments and imputations, 22 million variants from 2830 children were included for analysis. Recurrent wheeze, a clinical outcome strongly correlated with asthma, reported from age 2 to 5 was used as the primary phenotype in our analysis.

**Results:** Our genome-wide association study (GWAS) identified loci on chromosome 17q12, a highly replicated loci for asthma, associated with recurrent wheeze. Genetic risk score analysis (GRS) and SNP-set kernel association test (SKAT), which calculates the accumulated effect of common and rare variations, respectively, identified sets of variants significantly associated with recurrent wheeze in childhood. Gene-environment interaction analysis identified variations correlated with increased wheeze prevalence in children who were exposed to prenatal smoking. Finally, gene–gene (i.e. epistatic) interactions were studied by constructing a network of inter-correlated variants via hierarchical clustering, a machine learning algorithm for grouping similar elements. This analysis identified 8 clusters of interacting genes linked with childhood wheeze.

**Conclusion:** Our results show that both genes and environmental exposures contribute to recurrent wheeze in children as young as age 2, which is associated with asthma diagnosis later in childhood. On-going analyses include additional asthma-related phenotypes such as longitudinal lung function and positive skin prick tests to allergens as well as environmental variables such as pet ownership and nutrition.

**Acknowledgements:** Computations were performed on resources and with support provided by the Centre for Advanced Computing (CAC) at Queen’s University in Kingston, Ontario. The CAC is funded by: The Canada Foundation for Innovation, the Government of Ontario, and Queen’s University. J.C receives funding from the Canadian Institutes of Health Research.

### A2 Asthma prevalence among children of immigrant parents

#### Ahmad Alzahrani^1^, Allan Becker^1^, Padmaja Subbarao^2^, Malcolm R. Sears^3^, Stuart E. Turvey^4^, Piushkumar Mandhane^5^, Elinor Simons^1^

##### ^1^Section of Allergy and Immunology and Children’s Hospital Research Institute of Manitoba, Department of Pediatrics and Child Health, University of Manitoba, Winnipeg, MB, Canada; ^2^Department of Pediatrics, Hospital for Sick Children, Toronto, ON, Canada; ^3^Division of Respirology, Department of Medicine, McMaster University, Hamilton, ON, Canada; ^4^Department of Pediatrics, British Columbia Children’s Hospital, Vancouver, BC, Canada; ^5^Division of Pediatric Respirology, Pulmonology & Asthma, University of Alberta, Edmonton, AB, Canada

**Correspondence:** Ahmad Alzahrani

*Allergy, Asthma and Clinical Immunology* 2019, **15(Suppl 1):**A2

**Background:** Worldwide variation in asthma prevalence suggests that environmental factors are critical to childhood asthma development [1]. We explored associations between childhood asthma and parental immigration status and length of stay in Canada.

**Methods:** We used data from the Canadian Healthy Infant Longitudinal Development (CHILD) prospective birth cohort. Parents reported their birthplace and length of stay in Canada. At age 5 years, children had skin prick testing (SPT) to common environmental and food allergens. Based on history and physical examination, CHILD Study healthcare providers identified children with asthma.

**Results:** Of 2642 sets of parents, 57.1% were both non-immigrants, 11.0% were both immigrants to Canada, 17.8% were one immigrant and one non-immigrant, and 14.1% did not record their birthplace. For 72.9% of both immigrant parents, at least one parent had lived in Canada for ≤ 10 years (new immigrants). Compared to children of both non-immigrants, children of both immigrants had greater adjusted odds of any positive SPT (sensitization) (OR 1.9, 95% CI 1.3–2.8) and asthma plus sensitization (allergic asthma) (OR 2.6, 95% CI 1.4–5.2). Compared to children of non-immigrants, children of both immigrants who had lived in Canada for > 10 years had greater adjusted odds of sensitization (OR 2.7, CI 1.4–5.2) and allergic asthma (OR 4.3, CI 1.6–11.9), while children of new immigrants had similar adjusted odds of sensitization and allergic asthma.

**Conclusions:** Similarities between children of new immigrant and non-immigrant parents may reflect globalization trends, adoption of westernised lifestyle, [2] and fewer environmental differences between Canada and their home country. Increased sensitization and allergic asthma among children of non-recent immigrants may suggest delayed social integration in Canada (because of different countries of origin [3] or a greater degree of cultural nesting that preserves the lifestyle of the source countries [4]), which may make them more susceptible to contributors of allergic diseases.

### A3

Abstract withdrawn

### A4 Immunotherapy preferences in Canadian seasonal allergy patients: a parallel physician–patient survey

#### Anne K. Ellis

##### Department of Medicine, Queen’s University, Kingston, Ontario, Canada

**Correspondence:** Anne K. Ellis

*Allergy, Asthma and Clinical Immunology* 2019, **15(Suppl 1):**A4

**Background:** The Allergy Patient Identification for Immunotherapy (AsPIRe) program was a parallel physician and patient survey. The objective was to examine physician and patient perceptions of seasonal allergy symptoms and their impact on patients, and to examine patient and physician attitudes to allergy immunotherapy (AIT) for seasonal allergies. AsPIRe was led by a steering committee and received research ethics board clearance from Queen’s University.

**Methods:** Allergists (17) from across Canada enrolled in the AsPIRe program and completed an on-line survey to collect demographic information and baseline perceptions. Allergists then recruited patients and completed paper-based parallel physician and patient questionnaires. Patients received an AIT informational booklet with their questionnaire. Patients who were AIT-naïve with no contraindication to AIT and 12 years of age and older met the inclusion criteria.

**Results:** The survey was in field from February 2018 to June 2018. A total of 141 allergist surveys and 136 patient surveys were completed. Mean age of patients was 30 years old (range 12–70). 57% of patients reported prior knowledge of AIT. 72% of patients reported seasonal allergies of longer than 5 years duration and in this subset of patients, 46% were at their first allergist visit. 73% of all patients indicated they would be likely or very likely to try sublingual immunotherapy (SLIT), if recommended by their allergist compared to 36% for subcutaneous immunotherapy (SCIT). Conversely, 10% of patients reported they would be unlikely or very unlikely to try SLIT compared to 46% of patients who would be unlikely or very unlikely to try SCIT if recommended by their allergist.

**Conclusions:** In this particular study cohort, when assessing preference for AIT options, Canadian patients were more likely to follow their allergists’ recommendation to initiate SLIT compared to SCIT, were this to be recommended.

**Acknowledgement:** This program was funded by Stallergenes Greer.

### A5 Household cleaning product use and respiratory health in early life: an analysis from the Canadian Healthy Infant Longitudinal Development (CHILD) Study

#### Jaclyn Parks ^1^, Christoffer Dharma^2^, Jeffrey Brook^3^, James Scott^3^, Stuart Turvey^4^, Piush Mandhane^5^, Allan Becker^6^, Padmaja Subbarao^3,7^, Anita Kozyrskyj^5^, Diana Lefebvre^2^, Malcolm Sears^2^, Lawrence McCandless^1^, Tim K. Takaro^1^

##### ^1^Faculty of Health Sciences, Simon Fraser University, Burnaby, Canada; ^2^Department of Medicine, McMaster University, Hamilton, Canada; ^3^Dalla Lana School of Public Health, University of Toronto, Toronto, Canada; ^4^Department of Pediatrics, University of British Columbia, Vancouver, Canada; ^5^Department of Pediatrics, University of Alberta, Edmonton, Canada; ^6^Department of Pediatrics & Child Health, University of Manitoba, Winnipeg, Canada; ^7^Department of Pediatrics, University of Toronto & Hospital for Sick Children, Toronto, Canada; ^8^Canadian Healthy Infant Longitudinal Development Study (all investigators listed in Online Supplement)

**Correspondence:** Tim K. Takaro

*Allergy, Asthma and Clinical Immunology* 2019, **15(Suppl 1):**A5

**Abstract**

**Introduction:** Asthma is the most common chronic disease of childhood. This study from the Canadian Healthy Infant Longitudinal Development (CHILD) birth cohort examines the frequency of use of 26 common household cleaning products at 3–4 months of age, and the relationships between product use and outcomes of wheeze, atopy and diagnosed asthma at age 3. The outcomes of this analysis can be used to corroborate another project currently underway by CHILD Investigators to understand inflammatory exposures within the home in early life.

**Methods:** Our sample consisted of 2138 children with complete exposure, covariate, and outcome data at 3 years. We examined household cleaning product exposure using questionnaire responses to create a cumulative Frequency of Use Score (FUS). Outcomes, defined by questionnaire and clinical assessments, among those in the highest quartile of FUS were compared to those in the lowest quartile using multivariate logistic regression modeling. Adjusted models were also used to determine if frequent use of specific cleaning products modified risk when compared to less frequent users of the same products.

**Results:** Following adjustment for potential confounders, 3-year-old children from a home with a very high frequency of any cleaning product use, as assessed at 3–4 months, had a higher odds of recurrent wheeze (OR 1.64; 1.08–2.53, p = 0.02), recurrent wheeze with atopy (OR 2.73; 1.15–7.23, p = 0.03), and asthma diagnosis (OR 2.14; 1.28–3.67, p = 0.004) when compared to children from a home with low product use. There was no increase in the odds of atopy among those living in a home with a very high level of cleaning product use. Several individual products were associated with recurrent wheeze, recurrent wheeze with atopy, and asthma at age 3, including air fresheners, glass cleaner, multi-surface cleaner, dusting polish or spray, purell-type hand cleaner, and oven cleaner. Many of these products also showed importance in machine learning methods used the development in an Indoor Environmental Exposure Index to assess inflammation in the home, complimenting results of this current work.

**Conclusions:** While household cleaning product use is associated with an increased risk for childhood wheeze and asthma, but not of atopy. These results suggest that effects on the airway early in life from frequent cleaning product use may be due to an innate, inflammatory response rather than an acquired allergic response.

**Funding sources:** Canadian Institutes of Health Research (CIHR), Allergy, Genes and Environment Network of Centers of Excellence (AllerGen NCE, Inc.).

### A6 Longitudinal study of patient profile and treatment patterns of omalizumab for patients with asthma refractory to inhaled medications

#### Jason K. Lee^1^, Suvina Amin^2^, Michelle Erdmann^2^, Atif Kukaswadia^3^, Jelena Ivanovic^3^, Aren Fischer^3^, Alain Gendron^2,4^

##### ^1^Evidence Based Medical Educator Inc. Toronto, On, Canada and Section Head of Asthma at Canadian Society of Allergy and Clinical Immunology, Toronto, Canada; ^2^AstraZeneca, Mississauga, ON, Canada; ^3^IQVIA, Toronto, ON, Canada; ^4^Department of Medicine, University of Montreal, QC, Canada

**Correspondence:** Jason K. Lee

*Allergy, Asthma and Clinical Immunology* 2019, **15(Suppl 1):**A6

**Background:** Omalizumab was approved in Canada in 2004 for patients with moderate to severe, persistent asthma. There is currently limited evidence on omalizumab patients, their demographic profile, treatment discontinuation patterns, and concomitant medication use within Canadian real-world settings. This study described Canadian patients treated with omalizumab, their discontinuation patterns, and assessed concomitant medication use.

**Methods:** Real-world patient data was sampled from IQVIA’s private and public claims databases using an observational retrospective study design. Patients with at least one omalizumab claim between February 2007 and June 2015 were included in this analysis. Time to discontinuation was measured using Kaplan–Meier curves and patients were censored after 36 months post omalizumab initiation. Concomitant medication use was evaluated in the 6 months before, during omalizumab use, and the 6 months post-discontinuation, and compared using McNemar’s test.

**Results:** The sample consisted of 1160 patients. The mean age of the study cohort on omalizumab initiation was 45 years. 47% of patients were still receiving omalizumab at 36 months; almost half discontinued within 24 months. Of those who discontinued, 29.5% discontinued within 12 months and 17.8% discontinued in the 12 to 24-month period. Patients aged 12–19 and greater than 65 years were observed to have the highest 36-month persistence on omalizumab in the cohort (50.7% and 54.1%, respectively). No significant difference was observed in the prevalence of SABA or LAMA use in the time 6 months before, during omalizumab use, and 6 months post-discontinuation. However, there was a significant decrease in the prevalence of ICS/LABA use before vs. after omalizumab therapy of 7.39% (2.96, 11.82) (95% CI); p = 0.0016).

**Conclusion:** This study demonstrated that almost half of patients’ refractory to inhaled therapies discontinued omalizumab within the first 24 months. Patients aged 20‒34 years had a significantly greater likelihood of discontinuing omalizumab vs. patients aged 12‒19 years.

### A7 The house dust mite SLIT-tablet is well tolerated in subjects with house dust mite allergic asthma

#### Marianne Witten^1^, Waltraud Emminger^2^, Jennifer Maloney^3^, Dorte Rehm^1^, Trine M. Kruse^1^, Glenis Scadding^4^

##### ^1^ALK, Horsholm, Denmark; ^2^Allergy Outpatient Clinic, Rennweg, Vienna, Austria; ^3^ALK, Bedminster, NJ, United States; ^4^Department of Allergy and Medical Rhinology, Royal National Throat, Nose and Ear Hospital, London, United Kingdom

**Correspondence:** Marianne Witten

*Allergy, Asthma and Clinical Immunology* 2019, **15(Suppl 1):**A7

**Background:** Six clinical trials with the house dust mite (HDM) SLIT-tablet (ALK, Denmark) have been conducted in subjects with allergic rhinitis and/or allergic asthma. Pooled safety data in HDM allergic asthma subjects were analyzed.

**Methods:** Safety data from 6 phase II/III RDBPC clinical trials including subjects with mild or moderate asthma were pooled and analyzed. Only data for the European and US approved dose of 12 SQ-HDM are presented. Asthma adverse events (AEs) were defined by the MedDRA query “Asthma/Bronchospasm”.

**Results:** Of the 4860 subjects in the trials, 3209 had asthma (951 12 SQ-HDM; 1085 placebo). Approximately 70% of asthma diagnoses were moderate in severity by GINA criteria. Regardless of asthma status, the proportion of subjects with AEs was numerically higher in the active group (with asthma 86%; without asthma 85%) compared to placebo (with asthma 70%; without asthma 64%). The most common AEs were local allergic reactions, mainly mild or moderate in severity. A numerically higher proportion of subjects with asthma (active 11%; placebo 10%) experienced asthma-related AEs than subjects without (< 1% both treatments). A higher proportion of subjects with moderate asthma experienced asthma-related AEs (13% both treatments) compared to subjects with mild asthma (active 7%; placebo 5%). Few subjects with asthma, mostly moderate in severity, discontinued treatment due to an asthma AE, regardless of treatment (active 1%; placebo < 1%). There were few serious asthma AEs (3 active; 3 placebo). One subject (12 SQ-HDM) with moderate asthma and a recent viral infection experienced a treatment-related serious asthma AE on day 1 of treatment; following a week of asthma worsening, the subject was hospitalized for 4 days and recovered.

**Conclusion:** HDM SLIT-tablet was well tolerated in the allergic asthma population. Asthma AEs were primarily associated with the presence of asthma and asthma severity rather than HDM SLIT-tablet treatment.

### A8

Abstract withdrawn

### A9 Developing an ‘urban greenness’ index, an approach based on Toronto census metropolitan areas

#### Myrtha Reyna-Vargas^1^, Erjia Ge^2^, Wendy Lou^1^

##### ^1^Biostatistics department, University of Toronto, Toronto, Ontario, Canada; ^2^Epidemiology department, University of Toronto, Toronto, Ontario, Canada

**Correspondence:** Myrtha Reyna-Vargas

*Allergy, Asthma and Clinical Immunology* 2019, **15(Suppl 1):**A9

**Background:** Associations between greenness exposure and asthma are inconclusive, probably due to the use of single dimensional measurements to assess exposure, such as residential-surrounding greenness or green space proximity, that might not be sufficient to adjust for complex modifiable factors associated with the exposures. We propose an urban greenness index with adjustment for variability between multiple inputs on the judgement of importance of each index attribute.

**Methods:** We generated an index to assess urban greenness exposure with attributes, including overall vegetation (NDVI), building height, proximity to green spaces, tree basal area and population density per km^2^ at the Census Metropolitan Area level using 15-min walk travel network buffers. The importance of each attribute was obtained by using preference matrices (simulated and completed by CHILD investigators and environmental experts), variability of matrices was adjusted for using an integrative Fuzzy Analytic Hierarchy Process (FAHP). Weights generated from FAHP triangular, trapezoidal and Gaussian functions were compared. To demonstrate the index applicability, an analysis was made using lung function outcomes and asthma diagnosis at 5 years from children living in Toronto.

**Results:** Based on the pilot study, an ‘urban greenness’ index was obtained from simulated preference matrices where the ranking of the attributes is NDVI, followed by proximity to green spaces, population density, tree basal area and building height. Attribute weights obtained by triangular and trapezoidal functions follow the same ranking order and were similar in quantity. Results from expert’s preference matrices are to be analyzed.

**Conclusion:** The creation of an urban greenness index though FAHP allows the exploration of associations with pulmonary function taking into consideration the multifaceted aspects of greenness exposure and adjusting for the variability originated when considering various experts opinions and knowledge. The index developed can be applied to broader cohorts related to asthma, allergies, and other related health conditions.

### A10 Subjective improvement of chronic rhinosinusitis symptoms in patients on omalizumab therapy for allergic asthma

#### Khaldon F. Abbas^1^, Safi Zaidi^1^, Deena Kobric^1^, Gordon Sussman^1,2^

##### ^1^Gordon Sussman Clinical Research Inc., Toronto, Ontario, Canada; ^2^University of Toronto, Toronto, Ontario

**Correspondence:** Khaldon F. Abbas

*Allergy, Asthma and Clinical Immunology* 2019, **15(Suppl 1):**A10

**Background:** According to multiple studies, there is a high prevalence of chronic rhinosinusitis (CRS) in patients with asthma [1]. Evidence has shown that the IgE-mediated effects of omalizumab treatment used concurrently to treat allergic asthma (AA), could serve as an effective treatment option for CRS [2]. This study evaluates the effect of omalizumab on self-reported improvements of CRS symptoms in patients receiving omalizumab as part of their AA treatment.

**Methods:** Chart review was performed and 26 AA patients on omalizumab with a history of CRS were identified and invited to complete questionnaires. Patients recorded the level of improvement of their olfaction, facial pain, nasal congestion, and rhinorrhea since stating omalizumab therapy. 37 questionnaires were completed in total at various time points.

**Results:** The mean age of patients was 51 years (range 25–69, M:F 14:12), and the mean duration being on omalizumab was 43.3 months (range 2.6–87.5 months). Subjects took doses ranging from 150 mg every 3 months to 750 mg monthly. Subjects with olfaction problems prior to omalizumab reported an average improvement of 40.1%, with 48.5% reporting at least 50% symptom improvement. Subjects with facial pain prior to omalizumab reported an average improvement of 77.3%, with 87.5% reporting at least 50% symptom improvement. Subjects with nasal congestion reported an average improvement of 70.2%, with 85.3% reporting at least 50% symptom improvement. Subjects with rhinorrhea reported an average improvement of 69.7%, with 78.8% reporting at least 50% symptom improvement. Overall average improvements across the 4 symptoms for all 37 questionnaires was 58.6%. Patients taking 151–300 mg omalizumab monthly reported the highest average improvement (83.5%), followed by ≤ 150 mg (66.4%), and then ≥ 300 mg (42.5%).

**Conclusion:** Self-reported improvement of CRS symptoms in patients with AA was significant. A well-designed study is warranted to further investigate efficacy of omalizumab as a treatment option for CRS patients with AA.

### A11 Trends in asthma epidemiology in Alberta from 1995 to 2015

#### Ana-Maria Bosonea^1^, Heather Sharpe^1^, Harissios Vliagoftis^1^, Larry Svenson^1^, Dean Befus^1^

##### ^1^Department of Medicine and Dentistry, University of Alberta, Edmonton, Alberta, Canada

**Correspondence:** Ana-Maria Bosonea

*Allergy, Asthma and Clinical Immunology* 2019, **15(Suppl 1):**A11

**Background:** Asthma is a chronic respiratory disease characterized by reversible bronchoconstriction and airway inflammation. According to Statistics Canada in 2014, 8.1% of Canadians aged 12 and older reported having asthma; there are an estimated 274,661 persons with asthma in Alberta [1]. Most epidemiological studies estimate prevalence and incidence using survey-based data, which has limitations [2, 3]. The Ontario Asthma Surveillance Information System (OASIS) group uses provincial health databases and a validated asthma definition: two or more ambulatory care visits and/or one or more hospitalization(s) for asthma in 2 years [4,5]. In Alberta, there are some studies using provincial databases, but most studies are restricted to ER visits and do not represent the entire asthma population [6–9]. We used extensive health databases and the same definition for asthma as OASIS to investigate and report on province-wide asthma prevalence, incidence and mortality from 1995 to 2015.

**Methods:** Data from administrative databases, provided by Alberta Health, was analyzed to determine age and sex specific prevalence, incidence and mortality of the asthma population. The population cohort is all individuals residing in the province of Alberta, ages 0 to 99 from 1995 to 2015. Kendall’s Tau coefficient test was used to ascertain whether differences in trends were statistically significant.

**Results:** Between 1995 and 2015, the age-standardized incidence of asthma has decreased from 1.57 to 0.67% in males and from 1.67 to 0.70% in females. Prevalence increased over the last 20 years for both genders from 3.93 to 12.06% in females and from 3.74 to 11.63% in males but at a decreasing rate. All-cause mortality is higher in asthmatics versus the non-asthmatic population.

**Conclusions:** The incidence of asthma is decreasing in both females and males while prevalence continues to increase but at a slower rate. All-cause mortality in asthma patients is higher than in non-asthmatics, but both are decreasing over time.

**Acknowledgements:** Alberta Strategy for Patient Oriented Research Support Unit (SPOR) Asthma Working Group, Respiratory Health Strategic Clinical Network

### A12 Symptomatology as predictors of positive patient-requested skin prick tests for environmental allergens

#### Chaocheng Liu^1^, Karl Narvacan^1^, Xiao Wu^2^, Wei Han^2^

##### ^1^Faculty of Medicine and Dentistry, University of Alberta, Edmonton, AB, Canada; ^2^United Health Centre, Edmonton, AB, Canada

**Correspondence:** Chaocheng Liu

*Allergy, Asthma and Clinical Immunology* 2019, **15(Suppl 1):**A12

**Background:** In primary care, it is common to see patients contribute symptoms to environmental allergens, and many request referrals for a skin prick test. The aim of this study is to identify symptoms that could be used as predictors of a positive environmental skin prick test to facilitate early diagnosis and management of allergic conditions.

**Methods:** Over 1-year period, patients visiting one family clinic in Alberta were screened. 132 adults with no known allergic conditions who requested referrals to a skin prick test were included in the study. A standardized patient survey was filled out, which consisted of demographic information and symptoms patients believed that were caused by environmental allergens. They underwent standard skin prick tests for 54 common environmental allergens in Alberta. Symptomatology and the results of skin prick tests were analyzed for measures of diagnostic reliability.

**Results:** The mean age of the total 132 participants was 47 (22 to 82) and 84 were females. The most common presenting symptoms for requesting a skin prick test were runny/stuffy nose, sneezing, and nasal congestion. 69% of the total population were tested positive for at least one environmental allergen. Having itchy eyes had the highest positive likelihood ratio (LR) for a positive test (LR+ 1.40, LR− 0.49) followed by runny/stuffy nose (LR+ 1.20, LR− 0.68). Discrete symptoms including post-nasal drip, headache, and chest tightness were unlikely to predict a positive test.

**Conclusion:** Among patients requesting referrals for a skin prick test, the ones presenting with itchy eyes or running/stuffy nose were more likely to have positive results. However, no isolated symptom can be used as a single indicator of a positive skin prick test to environmental allergens in patients who believe the symptom is caused by allergy. Taking appropriate history and performing a good physical examination before referral are strongly recommended.

### A13 Early lung function changes associated with wheezing phenotypes in the CHILD Study

#### Zihang Lu^1,2,#^, Christopher J. Olesovsky^1,3,#^, Melanie Emmerson^1^, Krzysztof Kowalik^1^, Meghan B. Azad^4,5^, Aimée Dubeau^1^, Per Gustafsson^6^, Diana L. Lefebvre^7^, Stuart E. Turvey^8^, Allan B. Becker^4^, Piush J. Mandhane^9^, Felix Ratjen^1^, Wendy Lou^2^, Theo J. Moraes^1^, Malcolm R. Sears^7^, and Padmaja Subbarao^1^

##### ^1^Department of Pediatrics, and Translational Medicine, SickKids Research Institute, The Hospital for Sick Children, Toronto, Ontario, Canada; ^2^Dalla Lana School of Public Health, University of Toronto, Toronto, Canada; ^3^Faculty of Medicine, University of Toronto, Toronto, Ontario, Canada; ^4^Department of Pediatrics and Child Health, University of Manitoba, Winnipeg, MB, Canada; ^5^Children’s Hospital Research Institute of Manitoba, Winnipeg, MB, Canada; ^6^Department of Pediatrics, Central Hospital, Skövde, Sweden; ^7^Department of Medicine, Faculty of Health Sciences, McMaster University, Hamilton, Ontario, Canada; ^8^Department of Pediatrics, Child & Family Research Institute, BC Children’s Hospital, University of British Columbia, Vancouver, BC, Canada; ^9^Department of Pediatrics, University of Alberta, Edmonton, Alberta, Canada

**Correspondence:** Zihang Lu

^#^Zihang Lu and Christopher J. Olesovsky contributed equally to this work

*Allergy, Asthma and Clinical Immunology* 2019, **15(Suppl 1):**A13

**Background:** The trajectory of lung function in infancy prior to development of asthma is controversial. Methodologies applicable to any age are helpful in tracking early life lung function changes associated with wheezing disorders. This study aims to determine trajectories of lung function associated with early life wheezing phenotypes.

**Methods:** A sub-cohort of Toronto participants in the Canadian Healthy Infant Longitudinal Development (CHILD) study underwent repeated pulmonary function testing from infancy using multiple breath washout and forced expiratory maneuvers methodologies. Three wheezing phenotypes were defined by symptoms over the first 3 years of life: transient wheezers (symptoms in the first 24 months only), recurrent wheezers (≥ 2 episodes by 3 years) and non-wheezers.

**Results:** Lung function was tested in infancy and 3 years in 43 transient wheezers, 42 recurrent wheezers and 95 non-wheezers. In infancy, lung clearance index (LCI) was significantly worse in transient compared to recurrent wheezers (mean difference (MD) = 0.47; 95% CI 0.07 to 0.86; p = 0.02) and non-wheezers (MD = 0.54; 95% CI 0.19 to 0.88; p = 0.002). These differences fully resolved by age three. At 3 years of age, LCI was significantly worse in recurrent wheezers compared to non-wheezers (MD: 0.52; 95% CI 0.25 to 0.80; p < 0.001) and transient wheezers (MD = 0.39; 95% CI 0.06 to 0.7; p = 0.02). Spirometry data provided complementary information. During infancy, recurrent wheezers had similar FEV_t_/FVC (FEV_0.5_/FVC for infancy and FEV_0.75_/FVC for preschool-age) z-scores to non-wheezers, whereas transient wheezers had significantly lower FEV_t_/FVC z-scores compared to non-wheezers (MD = − 0.76; 95% CI − 1.42 to − 0.11; p = 0.02). However, by 3 years of age recurrent wheezers were significantly lower than non-wheezers (MD = − 0.53; 95% CI − 0.93 to − 0.14, p = 0.01), whereas there were no significant differences between transient wheezers and non-wheezers.

**Conclusions:** The lung function of recurrent wheezers was similar to non-wheezers in the first year of life but the trajectory significantly differed with lower function at age three.

**Acknowledgements:** CIHR, AllerGen NCE, Don & Debbie Morrison, SickKids Foundation

### A14

Abstract withdrawn

### A15 Using latent class analysis to identify childhood wheeze phenotypes from birth to age 5 years

#### Ruixue Dai^1^, Maxwell M. Tran^1^, Theo J. Moraes^1^, Wendy Y. W. Lou^2^, Allan B. Becker^3^, Piush J. Mandhane^4^, Stuart E. Turvey^5^, Malcolm R. Sears^6^, Padmaja Subbarao^1^

##### ^1^Department of Pediatrics, University of Toronto & Hospital for Sick Children, Toronto, Ontario, Canada; ^2^Dalla Lana School of Public Health, University of Toronto, Toronto, Ontario, Canada; ^3^Department of Pediatrics & Child Health, University of Manitoba, Winnipeg, Manitoba, Canada; ^4^Department of Pediatrics, University of Alberta, Edmonton, Alberta, Canada; ^5^Department of Pediatrics, University of British Columbia, Vancouver, British Columbia, Canada; ^6^Department of Medicine, McMaster University, Hamilton, Ontario, Canada

**Correspondence:** Ruixue Dai

*Allergy, Asthma and Clinical Immunology* 2019, **15(Suppl 1):**A15

**Background:** Past birth cohort studies have derived longitudinal wheeze phenotypes that may account for heterogeneity in atopy, asthma, and lung function [1–3]. We used a data-driven approach to identify wheeze phenotypes from birth to age 5 years in the Canadian Healthy Infant Longitudinal Development (CHILD) Study, a multicenter, general population-based birth cohort.

**Methods:** Data on wheeze were prospectively collected at 9 time points between birth and age 5 years. Wheeze was defined by a positive parent response to, “In the last 3/6/12 months, has your child had a wheezing noise coming from his/her chest either with or without a cold?” Latent class analysis was used to derive phenotypes based on the longitudinal prevalence of wheeze. Weighted logistic regression models were used to examine associations between risk factors and phenotypes, and between phenotypes and clinical outcomes including atopy (positive skin prick test to ≥ 1 allergen) and diagnosed asthma at 3 and 5 years.

**Results:** Using data from 3154 children with wheeze data at ≥ 2 time points, five classes were identified: no wheeze (71.50%), transient-early (6.31%), late-onset (9.73%), intermediate-onset (9.64%), and persistent wheeze (2.82%). Compared to no wheeze, persistent wheeze had the strongest associations with maternal asthma (OR 3.33, 95% CI 2.19–5.06) and prenatal smoke exposure (OR 2.21, 95% CI 1.36–3.61). Late-onset and persistent wheeze were both significantly associated with atopy at age 5 years (OR 2.42, 95% CI 1.83–3.19; OR 1.88, 95% CI 1.14–3.12, respectively). Persistent wheeze had the strongest association with asthma at ages 3 (OR 71.59, 95% CI 39.41–130.05) and 5 years (OR 46.15, 95% CI 25.36–83.99).

**Conclusions:** Five wheeze phenotypes were identified in the CHILD Study to age 5 years: no wheeze, transient-early, intermediate-onset, late-onset, and persistent wheeze. Persistent wheeze had the strongest associations with asthma at ages 3 and 5 years.

**Acknowledgments:** The authors are grateful to the families who participated in the study, CHILD Study staff and investigators, and the Allergy, Genes and Environment (AllerGen) Network of Centres of Excellence.

### A16 Thymic stromal lymphopoietin (TSLP) polymorphism rs1837253 minor allele may provide protection from asthma by altering TSLP long isoform gene expression in nasal epithelial cells

#### Amy Moorehead, Raphael Hanna, Delia Heroux, Judah A. Denburg, Loubna Akhabir

##### Department of Medicine, McMaster University, Hamilton, Ontario, Canada

**Correspondence:** Amy Moorehead

*Allergy, Asthma and Clinical Immunology* 2019, **15(Suppl 1):**A16

**Background:** Genome wide association studies have identified a strong association of the single nucleotide polymorphism rs1837253 in the thymic stromal lymphopoietin (TSLP) gene with asthma and airway hyperresponsiveness. The functional role of rs1837253 in gene regulation in relation to clinical phenotypes is unclear. TSLP has two gene isoforms which exhibit functional differences. We investigated the TSLP isoform roles in response to a pro-inflammatory signal and the rs1837253 genotype in relation to the TSLP isoform regulation in nasal epithelial cells (NECs).

**Methods:** Nasal epithelial cells from 15 asthmatic individuals and 30 non-asthmatic individuals, were cultured to 90% confluency. Cells were exposed to poly(I:C) for 3, 6 and 24 h, and gene expression profiles were measured using qPCR. Protein level expression in the cell supernatants were assessed using Bio-Plex assays. Mouthwash samples were collected and DNA was isolated using QIAamp Blood Mini Kits (Qiagen). Genotyping was then performed using Taqman genotyping assay for rs1837253. Gene expression and protein secretion were compared between asthmatics and non-asthmatics, and among homozygous major, heterozygous and homozygous minor alleles for the rs1837253 genotype.

**Results:** The long isoform of TSLP was more responsive to poly(I:C) stimulation. Asthmatic individuals expressed increased TSLP protein secretion from NECs when compared to healthy controls. Additionally, the rs1837253 minor T allele was less inducible by poly(I:C) stimulation than the major C allele, with lower gene expression of the long isoform of TSLP.

**Conclusions:** Our results provide important insights into the dichotomy of the TSLP isoforms. The data suggest that the rs1837253 T allele may lead to asthma protective effects through differential binding and regulation of the expression of long form TSLP, mitigating allergic inflammatory responses. This evidence allows us to further understand the role of TSLP in allergic asthma and may potentially be utilized to further stratify patients for anti-TSLP therapy.

### A17 A comparison of clinical responses to Bermuda grass, birch, and ragweed extracts using nasal allergen challenge

#### Matthew Rawls^1,3^, Jenny Thiele^1,2,3^, Lisa M. Steacy^3^, Daniel E. Adams^3^, and Anne K. Ellis^1,2,3^

##### ^1^Department of Biomedical and Molecular Science, Queen’s University, Kingston, Ontario, Canada; ^2^Department of Medicine, Queen’s University, Kingston, Ontario, Canada; ^3^Allergy Research Unit, Kingston General Health Research Institute, Kingston, Ontario, Canada

**Correspondence:** Matthew Rawls

*Allergy, Asthma and Clinical Immunology* 2019, **15(Suppl 1):**A17

**Background:** Bermuda grass (BG) flourishes in warm, tropical locations and is a prevalent allergen of the southern United States. However, we have observed high rates of BG skin sensitization in the temperate climate of Kingston, Ontario. In this study, we investigated whether BG extract elicits allergic rhinitis (AR) symptoms similar to native allergens during a nasal allergen challenge.

**Methods:** 59 allergic and 32 non-allergic consenting participants that previously completed a nasal allergen challenge were included in this analysis. A single allergen extract (either BG, birch or ragweed) was administered in a series of increasing dilutions to both nostrils. Total Nasal Symptom Score (TNSS) and Peak Nasal Inspiratory Flow (PNIF) were collected at baseline and approximately 15 min after each allergen dilution. Qualifying allergen dose was classified as a TNSS ≥ 8 and PNIF fall ≥ 50%. Specific IgE (sIgE) was measured using ImmunoCAP assays from collected serum. All statistical analyses were performed using GraphPad Prism 7.0.

**Results:** BG allergics had significantly increased wheal size (p < 0.0001), sIgE levels (p < 0.0001), TNSS (p < 0.0001), and significantly decreased PNIF fall (p < 0.0001) compared to non-allergics when challenged with BG. No significant differences were observed in wheal size, sIgE levels, and nasal symptoms between BG, birch, and ragweed allergics. Wheal size was positively correlated with sIgE levels in BG allergics alone, BG participants (allergic and non-allergic), and all allergics (BG, birch, ragweed challenged) (Spearman r = 0.8650 p = 0.0010, r = 0.9422 p < 0.0001, r = 0.6020 p = 0.0001, respectively). Qualifying allergen dose was negatively correlated with wheal size in BG participants, and all allergics (Spearman r = − 0.7900 p < 0.0001, r = − 0.3656 p = 0.0106). Qualifying allergen dose was negatively correlated with sIgE in BG participants (Spearman r = − 0.7014 p = 0.0004).

**Conclusion:** BG extracts can induce significant AR symptoms in allergic participants from the Kingston area during allergen challenge. Although BG is a non-native allergen in Canada, allergic participants experience similar nasal symptoms compared to the native birch and ragweed allergens.

### A18 Cost-minimization analysis of introducing sublingual immunotherapy for the treatment of house dust mite allergic rhinitis in Ontario

#### Anne K. Ellis^1^, Eva Hammerby^2^, Courtney Abunassar^3^, Andrea Lau^3^

##### ^1^Division of Allergy & Immunology, Department of Medicine, Queen’s University, Kingston, Ontario, Canada; ^2^ALK-Abello A/S, Hørsholm, Denmark; ^3^PDCI, Toronto, Ontario, Canada

**Correspondence:** Anne K. Ellis

*Allergy, Asthma and Clinical Immunology* 2019, **15(Suppl 1):**A18

**Background:** The SQ^®^ HDM SLIT-tablet was recently approved by Health Canada as allergy immunotherapy (AIT) for the treatment of moderate to severe house dust mite (HDM) induced allergic rhinitis (AR)^1^. AIT is a 3-year treatment and has traditionally been delivered as subcutaneous (SCIT) injections, administered in the physician’s office. The sublingual immunotherapy tablet (SLIT-tablet) is suitable for at-home treatment after the first dose has been administered in the physician’s office. This analysis was done to understand the economic implications of introducing SQ^®^ HDM SLIT-tablet in Ontario, where SCIT is already an available treatment.

**Methods:** Based on the evidence available it was assumed that the SQ^®^ HDM SLIT-tablet has at least the same efficacy as SCIT, and thus a cost-minimization analysis (CMA) was deemed appropriate. A societal perspective was adopted in the model, including relevant costs such as; costs of medications, services of health care professional and patient resources. Costs and resources were based on published sources, where possible. When no published sources were available the input to the model was based on physician opinion. The time horizon used was 3 years, which corresponds to a standard treatment course of AIT. A discount rate of 1.5% was applied to all costs in accordance with CADTH guidelines^2^. To understand the robustness of the results, sensitivity analyses were performed.

**Results:** The CMA shows that the societal cost of a 3-year treatment with SCIT was 7420 CAD, compared to 5048 CAD if treated with SQ^®^ HDM SLIT-tablet, leading to an overall saving of 2372 CAD. The sensitivity analyses showed the results to be robust. Nurse time per injection visit as well as number of injections per vial had the biggest impact on the results.

**Conclusion:** The economic analysis shows that SQ^®^ HDM SLIT-tablet is a cost-minimizing alternative to HDM SCIT when considered from a social perspective in Ontario.

### A19 Cost-minimization analysis of introducing sublingual immunotherapy for the treatment of house dust mite allergic rhinitis in Quebec

#### Rémi Gagnon^1^, Eva Hammerby^2^, Courtney Abunassar^3^, Andrea Lau^3^

##### ^1^Association des Allergologues et Immunologues du Québec, Québec, Canada; ^2^ALK-Abello A/S, Hørsholm, Denmark; ^3^PDCI, Ottawa, Ontario, Canada

**Correspondence:** Eva Hammerby

*Allergy, Asthma and Clinical Immunology* 2019, **15(Suppl 1):**A19

**Background:** The SQ HDM SLIT-tablet was recently approved by Health Canada as allergy immunotherapy (AIT) for the treatment of moderate to severe house dust mite (HDM) induced allergic rhinitis (AR)[1]. AIT is a 3-year treatment and has traditionally been delivered as subcutaneous (SCIT) injections, administered in the physician’s office. The sublingual immunotherapy tablet (SLIT-tablet) is suitable for at-home treatment after the first dose has been administered in the physician’s office. This analysis was done to understand the economic implications of introducing SQ HDM SLIT-tablet in Quebec, where SCIT is already an available treatment option.

**Methods:** As there is no available evidence indicating that the SQ HDM SLIT-tablet should not have at least the same efficacy as SCIT a cost-minimization analysis (CMA) was deemed appropriate. A societal perspective was adopted in the model, including relevant costs such as; costs of medications, services of health care professional and patient resources. Costs and resources were based on published sources, where possible. In case no published sources were available the input to the model was based on physician opinion. The time horizon in the model was 3 years, which corresponds to treatment course of AIT. A discount rate of 1.5% was applied in accordance with CADTH guidelines [2]. To understand the robustness of the results, sensitivity analyses were performed.

**Results:** The CMA shows that the societal cost of 3 year treatment with SCIT was 5830 CAD, compared to 5075 CAD if treated with SQ HDM SLIT-tablet, leading to an overall saving of 755 CAD. The sensitivity analyses showed the results to be robust. Nurse time per injection visit as well as number of injections per vial had the biggest impact on the results.

**Conclusion:** The economic analysis shows that SQ HDM SLIT-tablet is a cost-minimizing alternative to HDM SCIT when considered from a social perspective in Quebec.

### A20

Abstract withdrawn

### A21 Molecular diagnostics as a predictor of efficacy of sublingual allergen-specific immunotherapy in pediatric patients

#### Lala Allahverdiyeva^1^, Afah Khalilova^1^, H. Salimzade^1^, Veronica M. Samedi^2^

##### ^1^Division of Pediatrics, Azerbaijan Medical University, Baku, Azerbaijan; ^2^University of Calgary, Alberta, Canada

**Correspondence:** Afah Khalilova

*Allergy, Asthma and Clinical Immunology* 2019, **15(Suppl 1):**A21

The success of allergen-specific immunotherapy (ASIT) depends on the accuracy of detection of the causative allergen or allergens, as well as the cross-reactive allergens. A molecular diagnostic is a valuable tool that helps to individualize diagnosis and treatment of allergic conditions in children [1, 2].

**Methods:** 79 children aged 5 to 18 years with respiratory allergy (atopic bronchial asthma and/or allergic rhinitis) were included in the study. 68 out of 79 (86%) were found to have sensitization to house dust mites Dermatophagoides Pteronyssinus and/or Dermatophagoides Farinae. All these patients underwent molecular diagnostics using ImmunoCAP (Phadia 100) to determine the major (r Der p1, r Der p 2) and minor allergens (r Der p10) of the house dust mites. 56 patients were found to have a high and medium sensitivity to these allergens.

These patients were divided into two groups: study group: 36 children with sensitivity to major allergens received sublingual therapy with a mixture of house dust mites (Dermatophagoides Pteronyssinus and/or Dermatophagoides Farinae), and control group: 20 children with sensitivity to minor allergens who received symptomatic basal therapy. To compare the efficacy of SLIT, we evaluated the clinical symptoms of respiratory allergy on a point scale.

The degree of severity of each symptom was assessed in scores from 0 to 3 before the start of therapy and after 6 months of treatment:

- 0—no symptoms (no manifestations);
- 1—mild symptoms (minimal manifestations)
- 2—moderate symptoms (moderate severity of symptoms);
- 3—severe symptoms (affecting the quality of life).

**Results:** At the beginning of the study, all children had similar clinical symptoms, with the average daily score in the SLIT group of 2.5 ± 0.7; and in the control group—2.3 ± 0.8 points. After 6 months of treatment, we found a significant difference between groups, where SLIT group of patients showed a daily score of 0.5 ± 0.2, and the control group 1.9 ± 0.5 (p < 0.05).

**Conclusion:** Molecular diagnosis helps to select patients and predict the efficiency of the allergen-specific immunotherapy performed in patients with respiratory allergy.

### A22 Aspirin exacerbated respiratory disease transcriptome analysis using RNA-Seq

#### V. S. Priyadharshini, Luis M. Teran

##### Department of Immunogenetics and Allergy, Instituto Nacional de Enfermedades Respiratorias, Mexico City, Mexico

**Correspondence:** V. S. Priyadharshini

*Allergy, Asthma and Clinical Immunology* 2019, **15(Suppl 1):**A22

**Background:** Aspirin-exacerbated respiratory disease (AERD) is a syndrome characterized by rhinosinusitis nasal polyps asthma and aspirin intolerance. The pathogenic mechanisms associated with AERD include overproduction of cysteinyl leukotrienes (CysLTs), increased CysLTR1 expression in the airway mucosa and decreased lipoxin and PGE2 synthesis. However, the pathophysiology is not fully understood.

**Aim:** To establish the transcriptome of the nasal airway epithelial cells derived from AERD patients in order to uncover the genes patterns during this disease.

**Methods:** Nasal airway epithelial cells were isolated from nasal polyps derived from AERD patients, as well as healthy cells from AERD patient from and normal controls. To reduce genetic variability, airway epithelial cells were isolated from normal tissue derived from AERD patients. RNA was poly (A) selected and was sequenced with 25 million reads per sample on the Illumina HiSeq 2000 platform. The raw output data of the HiSeq was preprocessed according to illumina Hi Seq standard protocol. Data analysis: The read mapping to specific regions of the reference genome was done with hisat2. Counting was done with a flexible overlap approach. Up-regulated genes should have a logarithmic fold change > log_2_ (1.5) and down-regulated genes < − log_2_ (1.5). The p-values were adjusted step wise using the Benjamini–Hochberg procedure, p-value at or below 0.05 are displayed in a heatmap. R package DESeq2 was used for differential expression analysis.

**Results:** We have successfully sequenced AERD Polyp (11), AERD Healthy Cells (9) and Healthy controls (4). Data from AERD polyps and AERD Healthy controls: Twenty genes had twofold mean regulation expression differences or greater. Seven genes were unregulated, and thirteen genes were down regulated including cell adhesion, defense and immunity, protein binding and metabolic process was identified using PANTHER. Significant enriched pathways were plasma membrane organization, macroautophagy, leukotriene metabolic process, protein localization to plasma membrane and drug catabolic process.

**Conclusion:** I would like to conclude that the current study will reveal novel genes transcripts involved in the disease pathogenesis which will in turn help in developing novel drug targets.

### A23 Effectiveness of an online continuing medical education program in allergic rhinitis

#### Geoffrey Saroea

##### Department of Medical Affairs, GlaxoSmithKline Consumer Health, Mississauga, Ontario, Canada

**Correspondence:** Geoffrey Saroea

*Allergy, Asthma and Clinical Immunology* 2019, **15(Suppl 1):**A23

**Background:** Allergic rhinitis (AR) is a common disorder affecting 20–25% of Canadians [1]. Intranasal corticosteroids (INCS) are a mainstay of AR treatment [2–4]. The aim of this review was to evaluate the effectiveness of an online Continuing Medical Education program (eCME) in the management of AR.

**Methods:** This was a 1-h accredited eCME that provided a review of the disease and its current management incorporating case studies. Health Care Professionals (HCPs) completed a survey prior to taking the program and a second 1, 2 months afterwards, assessing the change in their understanding and behaviour. The online program was initiated in January 2017 and available for a year.

**Results:** A total of 4543 HCPs, exceeding the target of 2000, successfully completed the program. Three provinces had the highest enrollment: 25.9% (British Columbia), 24.7% (Alberta) and 20.5% (Ontario). Upon completion of the educational program, HCPs were more confident in assessing allergic rhinitis (increase of 23% on a 5-point scale: 1 = not confident, 5 = very confident) and were more inclined to recommend over the counter nasal corticosteroids as the first choice for appropriate patients with allergic rhinitis (increase of 27% on a 5-point scale: 1 = no patients with AR, 5 = all patients with AR). In addition, HCPs reported that it was important to provide ocular relief in combination with 24-h nasal symptom relief (13% increase on a 5-point scale: 1 = not important, 5 = very important). Forty-one to sixty percent of HCPs reported a 16% increase in their patients with allergies who suffered from both nasal and ophthalmic symptoms. Ninety-eight percent of HCPs reported that this learning experience was relevant to their practice.

**Conclusions:** The results demonstrated that this online CME program was effective. HCPs reported a better understanding of allergic rhinitis and its current management, and in recommending an INCS as first-line treatment for appropriate allergic rhinitis patients.

## Immunology

### A24 Genetic panel testing in children with suspected primary immunodeficiencies and immune dysregulation: experiences in a pediatric tertiary care center

#### A. Falkenham^1^, T. Issekutz^1^, A. Vandersteen^2^, B. Derfalvi^1^

##### ^1^Division of Immunology, Department of Pediatrics, Faculty of Medicine, Dalhousie University, Halifax, NS, Canada; ^2^Division of Genetics, Department of Pediatrics, Faculty of Medicine, Dalhousie University, Halifax, NS, Canada

**Correspondence:** A. Falkenham

*Allergy, Asthma and Clinical Immunology* 2019, **15(Suppl 1):**A24

**Background:** Primary immunodeficiencies (PID) and immune dysregulatory diseases (IDD) represent a wide-spectrum of immune abnormalities, often with an underlying genetic cause. Starting in 2013, Immunology could order genetic panels for clinical PID phenotypes. These panels allow to better characterization of a patient’s underlying immune abnormality, potentially leading to changes in treatment. In this study, we reviewed: (1) demographics of patients sent for genetic testing, (2) success rate of identifying genetic causes of PID and IDD, and (3) whether a genetic diagnosis changed treatment/care.

**Methods:** Clinical and immunological phenotypes and genetic results (post-2013) were collected for IWK patients who had genetic testing for PID and IDD. For analysis, patients were categorized into phenotypes based on their clinical presentation and supporting non-molecular laboratory tests. Each patient was then assessed for whether their treatment changed due to genetic testing.

**Results:** 84 patients had immunologic genetic testing between 2013 and 2017 with the following clinical phenotypes: 35.4% autoimmune/hyper-inflammation, 21.5% auto-inflammatory, 16.9% CVID, 18.5% SCID, and 7.7% other PID. Potential genetic abnormalities were detected in 59.3% of patients, of whom 43.8% received a molecular diagnosis. The PID panel successfully identified underlying genetic abnormalities in 62.1% of patients. In contrast, the periodic fever panel only identified an abnormality in 8.3% of patients. Notably, 56.9% of patients are followed by multidisciplinary teams, of which, 16.2% had a change in treatment/care due to genetic results.

**Conclusion:** In this study, we demonstrated that genetic testing for PID and IDD was able to provide valuable, treatment-guiding information for select patients. As we continue this research, our aim is to characterize patient and family histories to determine whether these could better inform which patients would benefit from PID and IDD genetic testing.

### A25 Delivery of subcutaneous immunoglobulin by rapid “push” infusion for primary immunodeficiency patients

#### Reyna Altook^1^, Chrystyna Kalicinsky^1,2^, Marianne Miguel^1,2^, Tamar S. Rubin^3,4^

##### ^1^Department of Internal Medicine, University of Manitoba, Winnipeg, Canada; ^2^Section of Allergy and Clinical Immunology, University of Manitoba, Winnipeg, Canada; ^3^Department of Pediatrics and Child Health, University of Manitoba, Winnipeg, Canada; ^4^Section of Pediatric Allergy and Clinical Immunology, University of Manitoba, Winnipeg, Canada

**Correspondence:** Tamar S. Rubin

*Allergy, Asthma and Clinical Immunology* 2019, **15(Suppl 1):**A25

**Background:** Pump-free subcutaneous immunoglobulin (SCIg) self-administration by rapid push has been reported as a safe, effective and convenient method of immune globulin (Ig) replacement in patients with primary immune deficiency diseases (PID).

**Methods:** We conducted a retrospective review of all patients in enrolled the SCIg push program in Manitoba, Canada. We included adult patients (≥ 18 years old) receiving SCIg push for PID for at least 12-months. Eligible patients were either Ig replacement naïve, or had previously received Ig by intravenous infusion (IVIg). We extracted individual patient IgG levels before and after starting SCIg, as well as the number of systemic antibiotic courses filled before and after starting SCIg. We also reviewed patient-reported adverse events, and reasons for discontinuation from the program.

**Results:** Sixty-six patients were included, of whom 24 were naïve to Ig replacement, and 42 had previously received IVIg. Of naïve patients, the mean IgG levels 6 months prior to SCIg and 12 months after were 6.6 g/L (range: < 0.33 g/L–15.8 g/L), and 11.3 g/L (range: 6.52 g/L–16.1 g/L). The mean numbers of antibiotic prescriptions in the 12 months prior and 12 months after were 5.1 (range 1–15) and 4.6 (range 0–18). For patients previously receiving IVIG, the mean IgG levels 6 months prior and 12 months after were 8.5 g/L (range: 0.38 g/L–15.5 g/L), and 11.8 g/L (4.9 g/L)–39 g/L). The average number of antibiotic prescriptions 12 months prior and 12 months after starting SCIg was 4.6 (range 0–19) and 4.2 (range 0–13). Adverse events were all local and mild. Statistical analysis is pending.

**Conclusions:** SCIg push appears to provide adequate steady state IgG concentrations, and resulted in reduction in antibiotic use in patients with PID. Compared to IVIg, SCIg push provided higher average IgG levels, and appeared to result in reduced antibiotic use.

### A26 Mast cell responses to respiratory syncytial virus infection

#### Christopher R. Liwski, Liliana Portales-Cervantes, Ian D. Haidl, Jean S. Marshall

##### Department of Microbiology and Immunology, Dalhousie University, Halifax, Nova Scotia, B3H 1X5, Canada

**Correspondence:** Jean S. Marshall

*Allergy, Asthma and Clinical Immunology* 2019, **15(Suppl 1):**A26

**Background:** Mast cells are abundant at areas contacting the environment, allowing them to mobilise responses to pathogens. They are suggested to be involved in respiratory syncytial virus (RSV) infection symptoms by producing mediators also involved in allergic responses. However, their exact role in RSV infections remains unclear. We have shown that in response to RSV in vitro, human mast cells upregulate type I interferon (IFN) production and various cytokines and chemokines potentially involved in effector cell recruitment, tissue remodelling, and modulating inflammatory responses. Mast cell degranulation and leukotriene production in response to RSV may also be important. This study aims to further examine human mast cell responses to RSV in vitro and the role of mast cells in vivo in a mouse model of RSV infection.

**Methods:** Cord blood-derived human mast cells were treated with RSV (MOI of 3–4) for 90 min at 4 °C, warmed to 37 °C for 30 min and assessed for mediator production. Mast cell-deficient mice (Hello Kitty (Cpa3-Cre; Mcl-1^fl/fl^) and mast cell-containing littermates were infected intranasally and monitored for 7 days.

**Results:** Human mast cells produced a range of cytokines, chemokines and interferons following RSV treatment. However, they did not demonstrate short-term degranulation or LTC_4_ generation following RSV treatment. Mast cell-deficient and -containing mice demonstrated similar weight change in response to intranasal RSV infection. More detailed analyses of the role of mast cells in the immune response to RSV, including studies assessing mast cell stabilisers in vivo, are underway.

**Conclusion:** The mast cell response to RSV infection is a complex process. We hope our findings from in vitro studies of human mast cells and in vivo infection of mice will further our understanding of how mast cell responses to RSV might be modulated therapeutically to improve antiviral immunity and limit airway inflammation.

*Funded by AllerGen N.C.E.*

### A27 IL-4 modulates IFN responses by virus-infected human mast cells

#### Liliana Portales-Cervantes, Owen Crump, Jean S. Marshall

##### ^1^Dalhousie Inflammation Group, Department of Microbiology and Immunology, Dalhousie University, Halifax, Nova Scotia, Canada

**Correspondence:** Jean S. Marshall

*Allergy, Asthma and Clinical Immunology* 2019, **15(Suppl 1):**A27

**Background:** Mast cells can respond to respiratory viruses such as influenza A virus (IAV) and reovirus through the selective production of pro-inflammatory cytokines. Allergic asthma is associated with a Th2 driven inflammation and increased susceptibility to viral infections which are important triggers of asthma exacerbations. Interferons (IFNs) have a key role in controlling virus replication and their production has been reported to be lower in asthmatic compared to healthy subjects. Th2 cytokines may be involved in this difference in response. Mast cells respond to multiple Th2 cytokines and are an important source of IFNs. Here we analyzed whether mast cell IFN responses to reovirus and IAV are impaired in the presence of Th2 cytokines.

**Methods:** Cord blood-derived human mast cells (CBMC) were cultured in medium alone or stimulated with 10 ng/ml IL-4, IL-5, IL-9, IL-13, or 1 ng/ml IL-33 for 48 h followed by infection with 5 MOI reovirus type 3 Dearing or IAV (H1N1 A/CA/07/2009). Supernatants were harvested 24 h post infection for cytokine analysis. mRNA gene expression was analyzed by qPCR.

**Results:** IFN production by reovirus-infected CBMC was not impaired by pre-treatment with Th2 cytokines. Strikingly, IL-4 selectively enhanced the production of type I and type III IFNs. Similar results were observed in response to IAV. Although IL-4 and IL-13 share a common receptor, these responses were specific for IL-4. Reovirus-infected CBMC stimulated with IL-4 at the time of viral infection did not have enhanced IFN production. IL-4 pre-treated CBMC also had increased production of IL-6, CCL3, and CXCL10 in response to reovirus.

**Conclusions:** Our data suggest that IL-4 modifies mast cell IFN responses when administered prior to reovirus or IAV infection, likely through type I IL-4 receptors. We suggest that mast cell responses to viral infections might be substantially modified in asthmatics by IL-4-enhanced production of IFNs.

**Acknowledgements:** This work is supported by CIHR. Reovirus and IAV were kindly provided by Dr. Shashi Gujar and Dr. Craig McCormick, respectively.

### A28

Abstract withdrawn

### A29 Comparison of IVIg with reduced anti-A/anti-B isoagglutinins to IVIg without reduction of isoagglutinins using serology and monocyte monolayer assay

#### Selena Cen^1^, Laura Gromolard^1^, Donald Branch^1,2^

##### ^1^Canadian Blood Services, Toronto, Ontario, Canada; ^2^University of Toronto, Toronto, Ontario, Canada

**Correspondence:** Selena Cen

*Allergy, Asthma and Clinical Immunology* 2019, **15(Suppl 1):**A29

**Background:** Intravenous immunoglobulin (IVIg) has been used to treat a number of autoimmune/inflammatory diseases with few side effects. However, high doses of IVIG (1–2 g/kg) have been recognized as a cause of hemolytic anemia in non-O blood group patients. Hemolysis when observed has been due to anti-A and anti-B isoagglutinins contained in the IVIg preparations. These isoagglutinins reacting with patients’ red blood cells (RBCs) can result in hemolytic anemia through destruction of the patients’ isoagglutinin-coated RBCs via the mononuclear phagocyte system. Recently, one manufacturer has produced an IVIg whereby the anti-A and anti-B titers have been greatly reduced by an immunoaffinity chromatographic approach. We aimed to investigate whether IVIg depleted of isoagglutinins, used to opsonize group A1, B or A1B RBCs, induces significantly lower phagocytosis compared to non-depleted IVIg.

**Methods:** An indirect antiglobulin test (IAT) was performed to evaluate reactivity of anti-A and anti-B isoagglutinins in the two IVIg preparations using titration. A monocyte monolayer assay (MMA) was performed to examine the erythrophagocytosis of IVIg-opsonized A1, B, and A1B RBCs.

**Results:** Isoagglutinin-reduced IVIg had significantly lower anti-A and anti-B titers compared to non-reduced IVIg. Isoagglutinin-reduced IVIg opsonized A1, B and A1B RBCs showed a significant reduction in IAT titration comparing to non-reduced IVIg. Using 33 mg/ml of IVIg (to give the approximate high-dose IVIg that would be administered to a patient of 80 kg; equivalent to 2 g/kg), phagocytosis of non-reduced IVIg opsonized A1, B, and A1B RBCs was determined to be clinically significant using MMA, with a phagocytic index (PI) of 42, 18, and 31 respectively. However, phagocytosis was largely absent when isoagglutinin-reduced IVIg was used at the same concentration to opsonize A1, B, and A1B RBCs with PI of 3, 1, and 2 respectively. A PI of > 5 is considered potentially clinically significant. In addition, using activated monocytes and M1 inflammatory macrophages as our sources of MMA for phagocytosis analysis, PI of non-reduced IVIg opsonized RBCs is consistently higher than PI of reduced IVIg opsonized RBCs.

**Summary:** Phagocytosis observed using an MMA with isoagglutinin-reduced IVIg at an equivalent in vivo dose of 2 g/kg to opsonize RBCs predicted a non-hemolytic event if infused into a patient of group A1, B, or A1B. We conclude that isoagglutinin-reduced IVIg would provide fewer hemolytic events in patients requiring high-dose IVIg therapy.

### A30

Abstract withdrawn

### A31 Priming human mast cells with IL-4 reduces gene expression of viral recognition genes TLR2 and MyD88

####  O. Crump^1^, L. Portales-Cervantes^1^, I. Haidl^1^, J. S. Marshall^1^

##### Department of Microbiology and Immunology, Dalhousie University. Halifax, NS., Canada

**Correspondence:** O. Crump.

*Allergy, Asthma and Clinical Immunology* 2019, **15(Suppl 1):**A31

**Background:** Asthmatic patients show an increased susceptibility to respiratory viruses, which trigger asthma exacerbations^1^. It has been shown that asthma-associated Th2 cytokines IL-4 and IL-13 can impair anti-viral responses and may be involved in such susceptibility^1^. Mast cells are well known for their detrimental role in asthma. Recently, their role as sentinel cells of mucosal immunity has been highlighted^2^. Mast cells can respond to diverse respiratory viruses such as Respiratory Syncytial Virus (RSV) by producing pro-inflammatory cytokines^3^. In addition, mast cells become activated by multiple Th2 cytokines^4^.

**Methods:** The purpose of this work was to determine if IL-4 interaction with human mast cells may affect their involvement in anti-viral responses. Mature cord blood-derived human mast cells (CBMCs) (n = 7) were primed with 10 ng/ml IL-4 for 48 h. RNA was isolated and the expression of several genes involved in viral recognition, including MyD88, STING, PKR, TLR2, and TLR3, was determined by qPCR.

**Results:** It was found that the mRNA for TLR2 and for the adaptor protein MyD88, were downregulated in mast cells primed with IL-4. In contrast, expression of TLR3 in these CBMCs was upregulated due to IL-4 priming. TLR2 is known to recognize envelope glycoproteins of some viruses and initiates an antiviral signal that is propagated by MyD88 to induce an antiviral response.

**Conclusions:** Our results suggest that IL-4 decreases the expression of certain viral pattern recognition receptors, such as TLR2, and other antiviral signaling-associated proteins, such as MyD88 on mast cells. Decreased expression of these genes may negatively impact mast cells’ anti-viral immune responses against viruses such as RSV during asthma, which could contribute to the enhanced susceptibility to respiratory viruses in asthmatic.

**Acknowledgements:** This work was supported by CIHR

### A32

Abstract withdrawn

### A33

Abstract withdrawn

### A34 Good’s Syndrome: two cases of immunodeficiency and thymoma with varying clinical presentations

#### Angeliki Barlas^1^, Hien Reeves^2^

##### ^1^Department of Internal Medicine, University of British Columbia, Vancouver, British Columbia, Canada; ^2^Department of Allergy and Immunology, University of British Columbia, Kelowna, British Columbia, Canada

**Correspondence:** Angeliki Barlas

*Allergy, Asthma and Clinical Immunology* 2019, **15(Suppl 1):**A34

**Background:** Good’s Syndrome (GS) is a rare adult-onset condition often affecting patients between 40 and 70 years of age, and is characterized by immunodeficiency and thymoma [1,2 ]. Specifically, patients with GS exhibit features of hypogammaglobulinemia, a reduction of peripheral B-cells and CD4+ lymphopenia [2]. These patients have increased susceptibility to various infections as well as autoimmunity. Given the rarity of this condition, there have not been any reported cases of Good’s syndrome in Canada upon review of the current literature.

**Cases presentation:** A chart review was performed of two patients in our clinic in British Columbia with immunodeficiency and thymoma, one of whom presented with bacterial respiratory infections, and one who presented primarily with fungal infections and autoimmunity. Case 1 describes a 51-year-old man with Good’s syndrome who presented with 3 years of recurrent bronchial, sinus and ear infections prior to the diagnosis of thymoma on chest x-ray. Hypogammaglobulinemia was only discovered post-operatively in the intensive care unit following his thymectomy, when the patient was found to have had fevers, pancytopenia and septicemia. Case 2 describes a 68-year-old man who presented with a history of recurrent thrush and fungal nail infections, 4 years prior to the diagnosis of thymoma. One year following his thymectomy, he was found to have had immunodeficiency with low B and T cells as well as autoimmunity, although he never had known bacterial infections. Several years later, he passed away from metastatic pancreatic cancer.

**Conclusions:** Although GS is a rare cause of immunodeficiency, it must be considered in all patients who are diagnosed with thymoma. The two cases summarized in this report highlight the importance of recognizing immunodeficiency for patients with thymoma and initiating treatments promptly to ensure favorable outcomes.

**Consent to publish:** was obtained from the patient [or guardians of the patient] involved in this study.

### A35 Combination of autologous and allogeneic AML-DCs is a potent CTL Inducer: prospective use in AML immunotherapy

#### Seyed M. Moazzeni^1^, Kambiz Bagheri^1^ and Seyed S. Moazzeni^2^

##### ^1^Immunolgy Department, Tarbiat Modares University, Tehran, Iran; ^2^School of Medicine, Tehran University of Medical Sciences, Tehran, Iran

**Correspondence:** Seyed M. Moazzeni

*Allergy, Asthma and Clinical Immunology* 2019, **15(Suppl 1):**A35

**Background:** Dendritic cells (DCs) with stimulatory potential of anti-leukemic CTLs could be derived from AML blasts. In this study a different strategy for generation of anti-leukemic CTLs was evaluated.

**Methods:** Peripheral blood blasts of AML patients were differentiated into leukemic DCs with characteristic morphology, immunophenotype and functional activity of dendritic cells. The leukemic origin of DCs was approved by pattern of angiotensin-converting enzyme (ACE) expression. In the proposed method of this study (autologous-allogenic protocol), AML patient’s T cells were stimulated 2 rounds by autologous leukemic DCs and 1 round by allogenic DCs, converse to conventional procedures (autologous protocol) which T cells are stimulated by autologous DCs three times. Specific cytotoxicity, IFN-γ and IL-4 production and the pattern of CD8/CD4 ratio were examined and compared in primed T cells generated from each protocol. Autologous blasts, autologous normal cells and K562 cell line were used as targets for CTL assays.

**Results:** In addition to the morphological changes, upregulation of all DC associated phenotypic markers and increased T cell stimulatory potential in allogenic MLR were occurred during culturing period of leukemic DCs. ACE expression pattern and the presence of 15;17 translocation, in leukemic DCs, approved the leukemic origin of generated DCs. The anti-leukemic cytotoxicity of primed T cells in autologous-allogenic protocol was significantly higher than that of conventional protocol, although there was no significant difference in cytotoxicity against K562 and normal cells. An increase in CD8+ cells was also occurred by allogenic protocol. Regarding the IL-4 production no significant difference was seen between two protocols but the proposed method was significantly more successful in induction of IFN-γ production.

**Conclusion:** Collectively it seems that our proposed autologous-allogenic protocol could be a successful strategy for AML immunotherapy by the generation of more potent anti-leukemic CTLs without increasing the risk of auto-reactivity.

## FOOD ALLERGY/ANAPHYLAXIS

### A36 Are we causing this? Food protein induced enterocolitis syndrome (FPIES) as a possible consequence of early peanut introduction

#### Jason A. Ohayon^1^, Vince Wu^2^

##### ^1^Department of Pediatrics, McMaster University, Hamilton, Ontario, Canada; ^2^Department of Biochemistry and Biomedical Sciences, McMaster University, Hamilton, Ontario, Canada

**Correspondence:** Jason A. Ohayon

*Allergy, Asthma and Clinical Immunology* 2019, **15(Suppl 1):**A36

**Background:** Food protein induced enterocolitis syndrome (FPIES) is a non-IgE mediated allergic reaction characterized by delayed vomiting, diarrhea 2–4 h post-ingestion. Extreme cases can lead to shock. Etiology of FPIES is unknown and not felt to be related to early food introduction.

**Methods:** A retrospective chart review of high risk atopic infants undergoing oral peanut (PN) challenge were followed with post challenge communication to determine continued at home tolerance to PN. Initial in office dosing followed standard PN updosing to a final dose of 7.75 g over 90 min. If no initial allergic reaction occurred, parents were requested to continue dietary PN twice weekly at PN doses of 2 teaspoons/dose. Families were contacted after challenge to determine status of continued PN tolerance. In cases of delayed allergic response, suspicious of FPIES, a physician supervised repeat PN oral challenge was offered in hospital for confirmation. Standard FPIES protocol dosing (0.6 gm/kg) of PN protein was given spaced out equally in 3 equal portions, with monitoring 4 h post exposure, as per guidelines^(1)^.

**Results:** From 2017 Jan, 3/28 infants developed delayed PN vomiting, 2–3 h post exposure. Two of these developed after a period of initial 2+ week period of peanut tolerance and one occurred at home after initial PN updosing. One of the 3 was confirmed with FPIES from subsequent inpatient admission for repeat challenge as per above protocol. All three families have withheld PN reintroduction.

**Conclusions:** With increasing numbers of atopic infants challenged early to PN, allergic reactions may be seen. FPIES was confirmed in 1/28 and suspected in 2/28 infants challenged. Of concern, FPIES developed in infants initially tolerant to PN. Physicians encouraging early PN introduction in atopic infants should be aware of the possibility of non-IgE mediated reactions at home, with the possibility of FPIES, as a consequence of changing feeding practices.

**Statement of consent:** Consent to publish was obtained from the guardians of the patient involved in this study.

The patient completed the 3 doses without immediate reaction. At 2¼ h post initial PN dose the patient vomited. Ondansetron (Zofran) and IV fluids were administered with full recovery. Electrolytes and CBC were both within normal limits before and after emesis.

### A37 Engaging knowledge users: usability testing of a Canadian anaphylaxis emergency plan

#### Abeer Hegazi^1^, Susan Waserman^1^, Ernie Avilla^1^, Monika Kastner^2^

##### ^1^Division of Clinical Allergy and Immunology, Department of Medicine, McMaster University, Hamilton, Ontario, Canada; ^2^Knowledge Translation and Implementation, North York General Hospital, Toronto

**Correspondence:** Abeer Hegazi

*Allergy, Asthma and Clinical Immunology* 2019, **15(Suppl 1):**A37

**Background:** There are no published studies on the usability of Anaphylaxis Emergency Plans (AEP). Most have not had methodologically rigorous usability evaluations to reveal problems and errors, nor have they been tested on relevant knowledge end-users. Such rigorous usability evaluations are particularly important for increasing our understanding of possible deficiencies. Importantly, they have the potential to reveal the specific features, function, and mitigating factors associated with the current lack of appropriate implementation and use. Our objective was to conduct a usability evaluation of the Canadian Society of Allergy and Clinical Immunology (CSACI) AEP

**Methods:** *Population:* Study inclusion criteria: 1) adult patients (age ≥ 18) at risk of anaphylaxis; 2) children (age < 18 years) at risk of anaphylaxis who are able to self-administer an EAI or their caregivers if they are unable to self administer an EAI, and 3) staff at schools (i.e. teachers). Teacher Recruitment was a convenience sample within one school board. *Setting*: Usability sessions took place at the Clinical Skills Simulation Laboratory at McMaster university. *Primary outcome*: Correct use of the AEP to identify situations when it is most appropriate to administer an EAI and its proper technique

**Results:** 12 participants agreed to participate; 4 children, 6 parents, 2 school staff.

The sessions were 45–60 min, audio- and video-taped and transcribed verbatim. Participants were asked to demonstrate the use of the AEP using a simulation man in the context of multiple scenarios. After the simulation, participants were interviewed using a semi-structured, open-ended questionnaire and Likert-type questions about the format, features, and other qualities of the AEP. Overall satisfaction of the AEP was 90%, 90% of participants identified anaphylaxis symptoms, 50% demonstrated proper EpiPen injection technique, and 30% verbally said will call 911

**Conclusion:** Understanding the usability factors that affect end-user knowledge and use of CSACI *AEP* is critical during the development of AEPs

### A38 Prescribed medications as risk factors for severe anaphylaxis

#### John Molloy^1^, Ann Clarke^2^, Judy Morris^3^, Moshe Ben-Shoshan^1^

##### ^1^Division of Pediatric Allergy and Clinical Immunology, Department of Pediatrics, McGill University Health Centre, Montreal, Quebec, Canada; ^2^Division of Rheumatology, Department of Medicine, Cumming School of Medicine, University of Calgary, Calgary, Alberta, Canada; ^3^Department of Emergency Medicine, Sacré-Coeur Hôpital, Montreal, Quebec, Canada

**Correspondence:** John Molloy

*Allergy, Asthma and Clinical Immunology* 2019, **15(Suppl 1):**A38

**Introduction:** The prevalence of emergency department visits for anaphylaxis has increased recently. Several classes of prescribed medications are suggested to increase the risk of severe anaphylaxis including cardiac medications, specifically beta-blockers, angiotensin converting enzyme inhibitors (ACEIs) and angiotensin receptor antagonists (ARAs); psychiatric medications, specifically tricyclic antidepressants and monoamine oxidase inhibitors (MAOIS), and non-steroidal anti-inflammatory drugs (NSAIDS).

**Objective:** To examine whether regular use of cardiac or psychiatric medications or NSAIDS in adults is associated with an increased risk of severe anaphylaxis.

**Methods:** Physicians completed questionnaires on episodes of anaphylaxis presenting to the emergency department at Hôpital du Sacré-Cœur de Montréal and contributing factors. The exposure was regular use of beta-blockers, ACEIs, ARAs, tricyclic antidepressants, MAOIS or NSAIDS. Anaphylaxis severity was defined as either mild-skin or subcutaneous tissue involvement; moderate-respiratory, gastrointestinal or cardiac involvement; severe-hypoxia, hypotension or neurological compromise. Multinomial logistic regression was used to examine relationships between exposure medication use in adults and severity of anaphylaxis, including adjustment for potential confounding.

**Results:** Between July 2012 and October 2017, 397 adults of whom 40% were male, with a median age of 39.1 years (interquartile range 27.3–52.7) presented to the emergency department with anaphylaxis. Food was the trigger in 52.4% (95% confidence intervals (CI) 47.4–57.4) of presentations and exposure medications were regularly used by 17.4% (95% CI 13.6–21.7). At least one dose of epinephrine was administered in 71% (95% CI 67.1–76.2) to treat anaphylaxis. There was no evidence of associations between use of beta-blockers, ACEIs, ARAs, tricyclic antidepressants, MAOIS or NSAIDS and moderate (adjusted relative risk (aRR) 1.10, 95% CI 0.43–2.84) or severe anaphylaxis (aRR 1.39, 95% CI 0.45–4.33).

**Conclusions:** Cardiac, psychiatric and anti-inflammatory medications were not associated with increased severity of anaphylaxis. Our results suggest such medications may be less detrimental in patients at risk of anaphylaxis but larger studies are required.

### A39 Peanut oral immunotherapy: preliminary results from London, Ontario

#### Pascale Dupuis^1^, Samira Jeimy^1^, Harold Kim^1,2^

##### ^1^Division of Clinical Immunology and Allergy, Western University, London, Ontario, Canada; ^2^Division of Clinical Immunology and Allergy, McMaster University, Hamilton, Ontario, Canada

**Correspondence:** Pascale Dupuis

*Allergy, Asthma and Clinical Immunology* 2019, **15(Suppl 1):**A39

**Background:** Peanut allergy affects 1.1% of Canadians [1]. Peanut oral immunotherapy (OIT) is a new, life-changing intervention in which patients are exposed to small amounts of peanut daily to decrease the risk of reaction associated with accidental exposure.

**Methods:** Patients aged 3 to 18 were diagnosed with peanut allergy by clinical history, evidence of IgE sensitization, and/or failed oral challenge. For the desensitization process, defatted peanut powder is mixed in oat flour or lactose powder, and encapsulated by the hospital outpatient pharmacy. Patients are administered an initial dose of 1 mg of peanut flour (containing 0.5 mg peanut protein) under clinical observation. Patients continue this dose daily for 1 month, then return to clinic for dose escalation. Each visit entails 1 h of close observation for adverse reactions. After a 13-month long build-up phase to 500 mg (approximately one peanut), patients are transitioned to a maintenance dose of one Peanut M&M^®^ daily.

**Results:** To date, nine patients have completed build-up and have transitioned to a daily Peanut M&M^®^, and 55 more have begun therapy. A total of 11,460 doses have been administered, including 390 dose escalations. In our experience, peanut OIT is very well tolerated. One reaction required epinephrine. The subject tolerated subsequent doses after starting a regular inhaled steroid for asthma. One patient stopped treatment due to anxiety. One patient had anaphylaxis while on maintenance therapy after he ingested a substantial amount of peanut. There have been no reactions upon transition from peanut flour to Peanut M&M^®^.

**Conclusions:** Compared to the 6 month induction period in published protocols, our thirteen-month long protocol is conservative. However, we note a high tolerance for therapy, lower rates of adverse reactions and a lower dropout rate. Further research is needed to guide treatment after initiation of the maintenance phase.

**Statement of consent:** Consent to publish was obtained from all patients involved.

### A40 Benefits and risks of anaphylactic interventions: a systematic review and meta-analysis

#### Amanda J. Lee, Yosef Ellenbogen, Susan Waserman, Derek K. Chu

##### Department of Medicine, McMaster University, Hamilton, Ontario, Canada

**Correspondence:** Amanda J. Lee, Yosef Ellenbogen

*Allergy, Asthma and Clinical Immunology* 2019, **15(Suppl 1):**A40

**Background:** Although epinephrine, corticosteroids, and anti-histamines are currently used to treat anaphylaxis, they can also contribute adverse outcomes, including life-threatening arrhythmia [1–6]. Previous systematic reviews of randomized controlled trials (RCTs) have failed to provide any evidence to inform the efficacy and safety of these therapies [3–6]. In the absence of RCTs, observational studies are the ideal data source to examine the risks and benefits of interventions. In this review, we aim to evaluate the risks and benefits of epinephrine, corticosteroids, and anti-histamines, versus no treatment for anaphylaxis.

**Methods:** We searched MEDLINE, EMBASE, LILACS, ICTRP, and CENTRAL up to Jan 16, 2017. Selection criteria included RCTs, cohort studies, and case series involving anaphylaxis. Studies limited to anaphylaxis in-hospital or long-term care facilities, or in patients with mastocytosis or aspirin-exacerbated respiratory disease were excluded. We followed the Cochrane Handbook for Systematic Reviews and registered this review with PROSPERO.

**Results:** Database searches identified 3441 studies: 332 from MEDLINE, 2337 from EMBASE, 582 from LILACS, 190 from CENTRAL, none from ICTRP. After deduplication, 3052 unique records were screened independently and in duplicate, yielding 153 studies satisfying inclusion criteria. After full-text review, data will be extracted, risk of bias will be assessed using Cochrane risk of bias tools, and the overall quality of evidence for each outcome will be assessed using the GRADE approach [7]. Outcomes that will be presented include: critical clinical outcomes (mortality, cardiorespiratory arrest, biphasic reaction), hospital-related outcomes (hospital admission, ICU admission, length of hospital stay), and adverse treatment events (e.g. arrhythmia, myocardial infarction, hyperglycemia, diabetes). Meta-analyses will be by random effects models using established approaches, giving preference to adjusted estimates of treatment effect [8].

**Conclusion:** This methodologically rigorous systematic review will be the first to provide evidence-based data regarding the risks and benefits of common treatments for anaphylaxis.

### A41 Understanding iKT in a biomedical study of food allergy: a mixed methods approach

#### Emily Shantz^1^, Susan J. Elliott^1^, Jenna Dixon^1^, and Ann E. Clarke^2^

##### ^1^Department of Geography and Environmental Management, University of Waterloo, Waterloo, ON, Canada; ^2^Division of Rheumatology in the Department of Medicine, Cumming School of Medicine, University of Calgary, Calgary, AB, Canada

**Correspondence:** Emily Shantz

*Allergy, Asthma and Clinical Immunology* 2019, **15(Suppl 1):**A41

**Background:** *Genetics, Environment and Therapies: Food Allergy Clinical Tolerance Studies* (GET-FACTS) is a CIHR-funded project investigating food allergy. Based on 4 research pillars [identifying novel biomarkers for food allergy; environmental and genetic factors influencing tolerance to foods; and integrated knowledge translation (iKT)], project scientists collaborate with a steering committee of food allergy stakeholders to facilitate end user-driven research. The objective of this presentation is to report experiences and perceived outcomes associated with this model of knowledge creation using a mixed methods approach.

**Methods:** A mid-point touch-base survey was administered to all scientists and steering committee members to investigate levels of knowledge and perceptions of experiences with the iKT model employed. In addition, qualitative semi-structured interviews were conducted to gain a better understanding of their respective perceptions and experiences. Interviews were digitally recorded and transcribed verbatim for thematic analysis. The survey was completed by n = 15 scientists and n = 7 steering committee members, while n = 12 scientists and n = 8 steering committee members participated in interviews.

**Results:** Both groups indicated that their knowledge of the iKT process increased over the course of GET-FACTS, and steering committee members reported an improved understanding of the scientific process. Researchers were generally satisfied with communications among scientists and the steering committee, however, many expressed a desire for more opportunities to connect. Perceived outcomes included positive impacts on the scientists’ research, the work produced by end user organizations, and the dissemination of GET-FACTS research findings.

**Conclusions:** GET-FACTS researchers demonstrated a range of experiences and perceived outcomes related to the iKT process in which they participated. These findings will inform future iKT practices among GET-FACTS researchers as well as the development and dissemination of a Performance Measurement Framework to evaluate the model for use in future health research.

**Acknowledgements:** This study was conducted on behalf of the GET-FACTS Steering Committee (https://uwaterloo.ca/get-facts-knowledge-translation/about/people).

### A42

Abstract withdrawn

### A43 Food protein-induced enterocolitis syndrome: a single institution review at Halifax Asthma and Allergy Associates

#### Laura A. Murphy^1^, Lori A. Connors^2^, Sandeep Kapur^1^, Greg Rex^1^, Gina Lacuesta^2^, Mary McHenry^1^

##### ^1^Department of Pediatrics, Division of Allergy & Immunology, Dalhousie University, Halifax, Canada; ^2^Department of General Internal Medicine, Division of Allergy & Immunology, Dalhousie University, Halifax, Canada

**Correspondence:** Laura A. Murphy

*Allergy, Asthma and Clinical Immunology* 2019, **15(Suppl 1):**A43

**Background:** Food Protein-Induced Enterocolitis Syndrome (FPIES) is a non-IgE-mediated disorder seen in children and adults. FPIES is triggered by the ingestion of specific food proteins resulting in vomiting and diarrhea 1–4 h after ingestion.

**Methods:** A retrospective, descriptive chart review was conducted on patients seen at the Halifax Asthma and Allergy Associates between January 2014 and June 2017 with a suspected diagnosis of FPIES. FPIES was confirmed based on predetermined clinical criteria of vomiting and diarrhea within 4 h of ingestion. Demographic and clinical characteristics were collected from electronic medical records for analysis.

**Results:** A total of 235 cases were reviewed. Twenty subjects > 18 years of age and 83 children < 18 years of age fulfilled the FPIES criteria. In our pediatric group, the majority of patient’s (64%) were under 36 months of age with cow’s milk being the most common food trigger (36%) followed by egg, grains and soy. In children > 36 months and < 18 years of age, shellfish was the most common food trigger (75%) followed by fish, and egg. FPIES reactions were also identified to poultry, beef, fruits, vegetables, and legumes. Sixty-three children had between 1 and 4 FPIES episodes (median 2 per child), remaining 20 children > 4. Fifty-nine (71%) children reacted to a single food. Vomiting occurred in 93% of children. Skin prick tests (SPT) were negative to trigger food in 94% of pediatric cases. Fifty-one (61%) children were atopic and 30 (36%) had a positive family history of atopy. Twenty-eight (33%) children had oral food challenges (OFC), 46% successfully ate a serving size of the trigger food based on age. Oral food challenges were deferred in children with recent accidental exposures who reacted to trigger food. In our adult group, shellfish was the most common food trigger (75%), followed by fish. Seventeen (85%) adults reacted to a single food. Vomiting occurred in 100% of adults. SPT were negative to trigger food in 95% of adult cases. Nine (45%) adults were atopic.

**Conclusion:** FPIES reactions were similar to previous reports with vomiting being a prominent feature. The most common food trigger identified in both pediatric and adults was shellfish. Clinician-supervised OFC was used in pediatrics. Management involves avoidance and education.

### A44 Barriers to epinephrine use by parents of children with anaphylaxis

#### Keelia Farrell^1^, Mona Jabbour^1,2^, Simon Hotte^1^, Waleed Alqurashi^1,2^

##### ^1^Department of Pediatrics, University of Ottawa, Ottawa, Ontario, Canada; ^2^Clinical Research Unit, Children’s Hospital of Eastern Ontario, Ottawa, Ontario, Canada

**Correspondence:** Keelia Farrell

*Allergy, Asthma and Clinical Immunology* 2019, **15(Suppl 1):**A44

**Background:** One of the commonly cited reasons by parents for not giving epinephrine in the prehospital setting is the inability to recognize the symptoms of anaphylaxis. The main goal of this study is to assess parents’ ability to recognize the symptoms of anaphylaxis and to correctly make the decision to give epinephrine when appropriate.

**Methods:** We prospectively surveyed parents of patients presenting to the CHEO Emergency Department (ED) with a diagnosis of food-induced anaphylaxis. Our Needs Assessment Survey for Anaphylaxis (NASA) tool consists of 2 parts: characteristics of previous and presenting anaphylaxis, and a validated Food Allergy Knowledge Test (FAKT) [1] to evaluate participant’s knowledge of recognition and treatment of anaphylaxis. The NASA survey is administered through RedCap. Monetary incentives were used to enhance the participation rate.

**Results:** Preliminary results from the first 15 families enrolled showed that 73% gave the epipen in the prehospital setting and the median number of previous anaphylaxis reactions was 3.5. Half of respondents perceived the reaction as life threatening and half called 911. The most frequent barrier to epinephrine use reported by parents was difficulty recognizing symptoms, although most parents still felt confident that this would not be a significant barrier. While mean score on the FAKT was 75%, many parents demonstrated that they could identify isolated symptoms of anaphylaxis but were unsure whether to give epinephrine when presented with a “real-world” scenario.

**Conclusions:** The higher percentage of parents who gave epinephrine in the prehospital setting than previously reported may be attributed to the high level of education of most parents and the high number of anaphylactic reactions children in our study. Based on preliminary data, parents are better able to identify the symptoms of anaphylaxis in isolation than when presented with “real world” scenarios. We hope to use these data to guide the creation of an e-module for parents that focuses on anaphylaxis recognition and treatment.

### A45 The use of a counselling video during an oral immunotherapy counselling session to improve parent and patient knowledge

#### Douglas P. Mack^1,2^, Mariam A. Hanna^1,2^

##### ^1^Halton Pediatric Allergy, Burlington, Ontario, Canada; ^2^Department of Pediatrics, McMaster University, Hamilton, Ontario, Canada

**Correspondence:** Douglas P. Mack

*Allergy, Asthma and Clinical Immunology* 2019, **15(Suppl 1):**A45

**Background:** Oral immunotherapy (OIT) for food has been studied in the pediatric population in phase 2 and phase 3 trials, and the use of OIT in private practice has been expanding in Canada. Families and physicians are interested in this approach of actively managing food allergy however misconceptions are common and effective education of these families is essential prior to properly obtaining informed consent. We investigated the use of a counselling video to improve parent and patient knowledge about OIT.

**Methods:** This is a quality assurance review conducted from 2017 to 2018 in a pediatric outpatient clinic. After initial consultation and review of information package, 209 parent and patient (> 12 years of age) participants performed pre- and post-tests in conjunction with a counselling video during a 2 h counselling session for OIT. This was a mandatory session prior to obtaining informed consent. 21 questions pertaining to OIT were asked of each patient both prior to and after watching a 45 min video. We reviewed the answers with each participant after completion of the post-test.

**Results:** Evaluation of pre- and post-test performance suggested an improvement in the ability of patients to answer relevant questions pertaining to OIT. This was statistically significant for all groups including mothers, fathers and children. Mothers performed better than fathers in both pre- and post-tests. Patient scores were not statistically different than parents. Importantly, this survey revealed no major deficiencies in understanding of key principles of OIT at the end of the counselling session. Reported satisfaction was high amongst participants.

**Conclusion:** This is the first study to evaluate the use of a counselling video in order to educate families about the key principles of OIT. We suggest that as part of extensive counselling for OIT that an educational video is beneficial in a pediatric outpatient clinic.

### A46 Update on peanut and tree nut oral immunotherapy—Canadian Pediatric Private Practice Oral Immunotherapy using NuT Suspension. (Canadian—PPPOINTS)

#### Mariam A. Hanna^1,2^, Douglas P. Mack^1,2^

##### ^1^Halton Pediatric Allergy, Burlington, Ontario, Canada; ^2^Department of Pediatrics, McMaster University, Hamilton, Ontario, Canada

**Correspondence:** Mariam A. Hanna

*Allergy, Asthma and Clinical Immunology* 2019, **15(Suppl 1):**A46

**Background:** As the incidence of food allergies continues to rise, potential therapies are undergoing investigation. We previously reported the first Canadian pediatric peanut and treenut oral immunotherapy (OIT) experience. Families are keenly interested in this approach of actively managing food allergy despite our lack of knowledge of long term outcomes. We report an update of pediatric patients undergoing peanut and treenut OIT in an outpatient clinic.

**Methods:** This is a retrospective review from 2014 to 2018 of OIT in a pediatric outpatient clinic (8 months to 17 years). After initial evaluation, testing, detailed informed consent/assent and potential oral challenge, participants were given the opportunity to be desensitized to peanuts and treenuts. A patient could be desensitized up to 3 nuts at one time. Peanut and treenut flours were suspended in a suspension medium and were given daily as sublingual/oral administration up to 50 mg nut protein in suspension and then transitioned to actual weighed nut. Doses were increased in clinic on a biweekly basis.

**Results:** Oral desensitization was successfully completed in a high majority of the patients who enrolled. The majority of individuals reported mild side effects. However, moderate to severe reactions were reported in several individuals during build up and maintenance requiring epinephrine use. Cofactors for reactions such as asthma, illness, empty stomach and compliance were identified. Side effects diminished with prolonged use. Skin prick testing decreased after being on maintenance therapy for 6–12 months.

**Conclusion:** This is an update on the first Canadian data to report on the safety and efficacy of pediatric oral immunotherapy in private practice for peanut and tree nut allergies. A suspension medium allowed for safe dosing titration. As knowledge of immunotherapy increases, allergists will need to become comfortable utilizing these approaches for the active management of food allergies.

### A47 The role of maternal TLR2 during breastfeeding on oral tolerance development

#### Bassel Dawod^1^, Alexander Edgar^2^, Matthew C. Tunis^2^, Jean S. Marshall^1,2^

##### ^1^Department of Pathology, Dalhousie University, Halifax, NS, Canada, ^2^Department of Microbiology & Immunology, Dalhousie University, Halifax, NS, Canada

**Correspondence:** Bassel Dawod

*Allergy, Asthma and Clinical Immunology* 2019, **15(Suppl 1):**A47

**Background:** Breastfeeding has beneficial effects on the development of oral tolerance towards food allergens. Multiple factors in breast milk help shape the immune system, including cytokines, growth factors, and beneficial bacteria. Toll-like receptor-2 (TLR2) activation by pathogen products can lead to tolerance disruption. Human milk also contains Soluble TLR2 (sTLR2) that acts as a decoy receptor, but its role in oral tolerance is unknown.

**Hypothesis:** We hypothesise that either a TLR2 deficient maternal in utero environment or breast milk from mothers deficient in TLR2 does not support the normal development of oral tolerance.

**Methods:** A murine cross-fostering model was designed to evaluate the impact of maternal TLR2 and breast milk-associated factors on tolerance development. Pups from crosses of male TLR2^−/−^ with female TLR2^+/+^ and male TLR2^+/+^ crossed with female TLR2^−/−^ were divided into two groups, such that half of each litter remained with its biological mother while the other half was cross fostered by a mother of the alternate genotype. The pups were exposed to 20 µg/ml ovalbumin daily during lactation (day 10–17) to induce tolerance, which was assessed by measuring serum anti-OVA IgE responses following i.p. OVA immunisation and levels of T-regulatory cells (Tregs) in the intestine of the pups after weaning.

**Results:** The most effective oral tolerance generation, marked by low anti-OVA IgE (*P *= 0.0065) in serum, higher intestinal Tregs (*P *= 0.0477), and decreased permeability of the intestine occurred in the animal nursed by TLR^+/+^ mothers regardless of their in utero exposure to maternal TLR2.

**Conclusions:** Our results confirm an important role for TLR2 in the development of oral tolerance, by a breast milk dependent mechanism.

**Funding source:** Supported by CIHR and Allergen-NCE.

### A48 Reactions to known food allergens in pediatric patients

#### Laura May Miles^1^, Sofianne Gabrielli^1^, Ann Clarke^2^, Judy Morris^3^, Jocelyn Gravel^4^, Paul Enarson^5^, Edmond S Chan^6^, Andrew O’Keefe^7^, Robert Porter^8^, Rodrick Lim^9^, Moshe Ben Shoshan^1^

##### ^1^Division of Allergy and Clinical Immunology, Department of Pediatrics, Montreal Children’s Hospital, McGill University Health Centre, Montreal, QC, Canada; ^2^Division of Rheumatology, Department of Medicine, Cumming School of Medicine, University of Calgary, Calgary, Alberta, Canada; ^3^Department of Emergency Medicine, Sacré-Coeur Hôpital, Montreal, Quebec, Canada; ^4^Division of Emergency Medicine, Department of Pediatrics, Centre hospitalier universitaire Sainte-Justine, Montreal, Quebec, Canada; ^5^Division of Emergency Medicine, Department of Pediatrics, BC Children’s Hospital, University of British Columbia, Vancouver, British Columbia, Canada; ^6^Division of Allergy and Immunology, Department of Pediatrics, BC Children’s Hospital, University of British Columbia, Vancouver, British Columbia, Canada; ^7^Department of Pediatrics, Faculty of Medicine, Memorial University, St. John’s, Newfoundland & Labrador, Canada; ^8^Discipline of Pediatrics, Faculty of Medicine, Memorial University, St. John’s, Newfoundland & Labrador, Canada; ^9^Division of Pediatrics and Emergency Medicine, Children’s Hospital at London Health Science Centre, London, Ontario, Canada

**Correspondence:** Laura May Miles

*Allergy, Asthma and Clinical Immunology* 2019, **15(Suppl 1):**A48

**Background:** Over the past two decades, awareness for food allergies has been on the rise, thus resulting in an increased demand for allergen labeling on pre-packaged food. The risk of pediatric patients with a known food allergy presenting with anaphylaxis to the same food is unknown. We aimed to assess the percentage of patients with anaphylaxis and known food allergy presenting with anaphylaxis to the same food allergen, and which food allergens are the most common culprits for repeat anaphylactic reactions.

**Methods:** Over a 6-year period, children presenting to six Emergency Departments (ED)s in British Columbia, Ontario, Quebec, and Newfoundland with anaphylaxis were recruited as part of the Cross-Canada Anaphylaxis Registry (C-CARE) study. Cases were recruited either at the time of ED presentation (prospective) or identified using a previously validated algorithm based on ICD-10 diagnosis codes related to anaphylaxis (retrospective). A standardized data entry form was used to collect data on patients’ demographics, co-morbidities (e.g. presence of a known food allergy, presence of asthma), and triggers of anaphylaxis.

**Results:** From 2011 to 2017, 1323 pediatric patients with a known food allergy presented to six pediatric EDs across Canada with an anaphylactic reaction to food, of which 61.9% (95% CI 59.3%, 64.5%) were male, with a median age of 6.5 years (interquartile range [IQR]: 3.1, 11.7). Among patients with anaphylaxis and known allergy to peanut, 42.81% (95% CI 39.00%, 46.71%) presented with anaphylaxis to peanut. Cases with anaphylaxis and known milk allergy and cases with anaphylaxis and known tree-nut allergy presented with food-induced anaphylaxis to the corresponding allergens in 42.26% (95% CI 36.29%, 48.47%) and 37.82% (95% CI 32.12%, 43.86%) of cases respectively.

**Conclusion:** Our findings indicated that in those with anaphylaxis and a known food allergy, peanut, tree-nut and milk are the most common culprits for repeat anaphylactic reactions.

### A49 Rates of anaphylaxis for the most common food allergies

#### Bruce T. Miles^1^, Sofianne Gabrielli^1^, Ann Clarke^2^, Moshe Ben-Shoshan^1^

##### ^1^Division of Pediatric Allergy and Clinical Immunology, Department of Pediatrics, McGill University Health Center, Montreal, QC, Canada; ^2^Division of Rheumatology, Department of Medicine, Cumming School of Medicine, University of Calgary, Calgary, Alberta, Canada

**Correspondence:** Bruce T. Miles

*Allergy, Asthma and Clinical Immunology* 2019, **15(Suppl 1):**A49

**Background:** Approximately 7.5% of Canadians have a known food allergy^1^. Although it has been suggested that the prevalence of food allergy and food-induced anaphylaxis has increased over the last decade^2^, it is not clear if there are temporal trends in food triggers of anaphylaxis. We aimed to assess the rate of anaphylaxis caused by the nine most common food allergens in children at the Montreal Children’s Hospital (MCH) over the past 6 years.

**Methods:** Over a 6-year period, children presenting to the Emergency Department (ED) at the MCH with anaphylaxis were recruited as part of the Cross-Canada Anaphylaxis Registry. A standardized form documenting symptoms and triggers of anaphylaxis was completed by an ED physician at the time of presentation (i.e., prospective recruitment). Missed cases were identified according to a previously validated algorithm based on ICD-10 codes related to anaphylaxis. Descriptive statistics were used to compare yearly rates for anaphylactic reactions for each food allergen.

**Results:** From April 2011 to March 2017 at the MCH, 1407 cases of food-induced anaphylaxis were recruited of which 36.6% were prospective. Over half (59.4% [95% CI 56.8%, 62.0%]) were males with a median age of 5.0 years (IQR 2.0, 10.6). From 2011 to 2017, there was a significant decrease in the yearly percentage of anaphylactic reactions to peanut (− 15.03% [95% CI − 24.19%, − 5.87%]. There was an increase the number of anaphylactic reactions attributed to tree nut (7.22% [95% CI 1.85%, 12.59%]). No significant change was found for the other seven common allergens. There has also not been any significant change in the percentage of food-induced anaphylaxis among all cases of anaphylaxis over the 6-year period (2.43% [95% CI, − 4.25%, 9.12%]).

**Conclusion:** A decrease in anaphylactic reactions to peanut may indicate an increased awareness of peanut allergy and vigilance. Educational programs promoting awareness and avoidance of tree nut allergens are required along with all other allergens.

### A50 Emergency management of adult and pediatric idiopathic anaphylaxis: a 6-year follow-up study in Canada

#### Michelle Le^1^, Sofianne Gabrielli^2^, Ann Clarke^3^, Judy Morris^4^, Jocelyn Gravel^5^, Edmond S. Chan^6^, Rod Lim^7^, Andrew O’Keefe^8^, Greg Shand^2^, Moshe Ben-Shoshan^9^

##### ^1^McGill University, Montreal, Quebec, Canada; ^2^Division of Clinical Epidemiology, McGill University Health Centre, Montreal, Quebec, Canada; ^3^Division of Rheumatology, Department of Medicine, Cumming School of Medicine, University of Calgary, Calgary, Alberta, Canada, Calgary, AB, Canada; ^4^Department of Emergency Medicine, Sacre-Coeur Hospital, Montreal, Quebec, Canada; ^5^Department of Emergency Medicine, CHU Sainte- Justine, Montreal, Quebec, Canada; ^6^Division of Allergy & Immunology, Department of Pediatrics, BC Children’s Hospital, Vancouver, British Columbia, Canada; ^7^Department of Pediatrics & Medicine, Children’s Hospital at London Health Sciences Centre, London, ON, Canada; ^8^Departmment of Pediatrics, Faculty of Medicine, Memorial University, St John’s, Newfoundland, Canada; ^9^Division of Pediatric Allergy and Clinical Immunology, Department of Pediatrics, Montreal Children’s Hospital, Montreal, QC Canada

**Correspondence:** Michelle Le

*Allergy, Asthma and Clinical Immunology* 2019, **15(Suppl 1):**A50

**Background:** Data regarding treatment and management of idiopathic anaphylaxis (IA) is very limited. This study aims to assess the demographics, clinical characteristics and management of adult and pediatric IA cases across Canada.

**Methods:** Data on 222 pediatric and 73 adult IA cases were prospectively collected between 2011 and 2018 in emergency rooms at 5 centres across Canada (Montreal Children’s, Sacré-Coeur, Sainte-Justine, Janeway Children’s and BC Children’s Hospitals) as part of the Cross-Canada Anaphylaxis Registry (C-CARE). Severe cases were defined as ones that manifested as cyanosis, hypoxia (saturation < 92%), respiratory arrest, hypotension, dysrhythmia, confusion or loss of consciousness. Factors associated with epinephrine treatment were identified using multivariate logistic regression.

**Results:** Among 295 IA cases, the median age was 9.0 years (IQR: 5.0, 14.3) and 38.1 (IQR: 8.1, 51.3) for children and adults, respectively. A significantly higher number of pediatric cases had a known food allergy, 43.9% (95% CI 37.3%, 50.7%), in comparison to adults, 20.5% (95% CI 12.3%, 31.9%). A statistically higher number of pediatric cases were severe, 36.0% (95% CI 29.7%, 43.2%), compared to adults, 6.8% (95% CI 0.0%, 14.6%). Epinephrine was not administered in 25.2% (95% CI 19.8%, 31.6%) of pediatric cases and 46.5% (95% CI 34.7%, 58.6%) of adult cases. Referral for allergist follow up was given to 76.1% (95% CI 69.6%, 81.5%) of pediatrics and 78.1% (95% CI 66.6%, 86.6%) of adult patients. Pediatric patients who were not treated with epinephrine either outside or inside the healthcare facility were more likely to have severe reactions (adjusted Odds Ratio (aOR) 1.14 [95% CI 1.00, 1.29]). In contrast, adult patients who were not treated with epinephrine either outside or inside the healthcare facility were more likely to have mild reactions (aOR 1.91 [95% CI 1.24, 2.94]).

**Conclusions:** Our findings highlight the need to increase awareness of appropriate management of IA with epinephrine followed by consultation with an allergist across Canada.

### A51 Does time matter? Challenging infants with atopic dermatitis and/or egg allergy to peanut less than a week after initial assessment may decrease their risk of peanut allergy

#### Vince Wu^1^, Claire Wilmer ^2^, Jason A. Ohayon^3^

##### ^1^Department of Biochemistry and Biomedical Sciences, McMaster University, Hamilton, Ontario, Canada; ^2^Department of Chemical Biology, McMaster University, Hamilton, Ontario, Canada; ^3^Department of Pediatrics, McMaster University, Hamilton Ontario, Canada

**Correspondence:** Vince Wu

*Allergy, Asthma and Clinical Immunology* 2019, **15(Suppl 1):**A51

**Background:** In 2017, the National Institute of Allergy and Infectious Diseases (NIAID) issued a recommendation of early peanut (PN) introduction to infants with severe atopic dermatitis (AD) and/or egg allergy. The outcome of early PN introduction has identified some infants unable to tolerate PN, the reasons of which may not be fully known.

**Methods:** A retrospective trial was carried out in a community clinic, identifying above infants with no history of PN exposure. Infants were offered PN in clinic and monitored. The protocol provided increasing amounts of PN flour to a final dose of 3.8 g of protein. Infants were monitored for allergic symptoms.

**Results:** Twenty-eight infants 4 to 12 months (median 7 months) were identified and challenged over an 18 month period. Twenty-three of 28 infants were tolerant (82.1%) to PN and 5/28 were allergic (17.9%). Allergic reactions included urticaria (1), vomiting (3), and anaphylaxis (1). Skin prick test was positive for 43% of tolerant patients with an average size of 4.3 mm, and positive for 40% of allergic patients with an average size of 4.5 mm. Seventeen infants were seen and challenged less than 1 week from their initial assessment, 15/17 (88.2%) infants were tolerant and 2/17 (11.8%) infants were allergic. Eleven infants were seen and challenged greater than 1 week from initial assessment, 8/11 (72.7%) infants were tolerant and 3/11 infants (27.3%) were allergic. Three infants initially tolerant to PN in office subsequently developed FPIES to PN.

**Conclusions:** Early PN introduction in atopic infants was safe in the majority of high risk infants as per NIAID. Time from assessment to challenge appears to influence outcome of PN tolerance. Challenging infants less than a week from their initial assessment decreased the risk of allergic response. Skin test size did not appear to differentiate between allergic and tolerant infants.

### A52 Simulation-based education to improve management of refractory anaphylaxis in an allergy clinic setting

#### Ana Copaescu^1^, Nathalie Nadon^2^, Rémi Gagnon^3^, Arnaud Robitaille^4^, Mohamed Badawy^5^, David Claveau^6^ Anne Des Roches^1^, Jean Paradis^1^, Matthieu Vincent^7^, Philippe Bégin^1^

##### ^1^Allergy-Immunology Division, Department of Medicine, Université de Montréal, Montréal, Québec, Canada; ^2^Department of Medicine, Université de Montréal, Montréal, Québec, Canada; ^3^Allergy-Immunology Division, Department of Medicine, Université Laval, Québec, Québec, Canada; ^4^Department of Anesthesiology, Université de Montréal, Montréal, Québec, Canada; ^5^Department of Anesthesiology, Montreal Neurological Institute and Hospital, Montréal, Québec, Canada; ^6^Department of Emergency, Université de Montréal, Montréal, Québec, Canada; ^7^Department of Emergency, McGill University, Montréal, Québec, Canada

**Correspondence:** Ana Copaescu

*Allergy, Asthma and Clinical Immunology* 2019, **15(Suppl 1):**A52

**Background:** High-fidelity simulations based on real life clinical scenarios have frequently been used to improve patient care, knowledge and teamwork in acute care setting but are seldom included in the allergy curriculum or continuous medical education [1]. Recent changes in staff and clinical environment motivated the creation of a simulation training program for severe refractory anaphylaxis at our hospital-based allergy clinic.

**Methods:** Advanced anaphylaxis life support scenarios were designed by a multidisciplinary panel of ER, ICU, anesthesiology and allergy specialists and then adapted for the allergy clinic setting. The first part of the program took place in the high-fidelity simulation-training laboratory and focused on updating participants competencies. The second part was an in situ simulation with live actors and a high-fidelity dummy at the allergy clinic, involving the code team and hospital security. Participants filled out a questionnaire on training objectives. We then performed qualitative interviews with staff after they had managed real cases of refractory anaphylaxis in the clinic.

**Results:** Four (4) nurses, 7 allergy fellows and 7 allergists underwent the simulation. Participant questionnaires showed perceived improvement in various aspects of crisis and anaphylaxis management. The in situ simulation revealed gaps in process, especially with regards to access to medication and material, intensive care response, code signaling and crash cart transport, which were subsequently resolved. Qualitative interviews with participants having dealt with anaphylaxis at the clinic revealed a more rapid and orderly response and an improved confidence in their abilities and that of other staff members to manage anaphylaxis.

**Conclusion:** High-fidelity simulations can improve management of anaphylaxis at the allergy clinic as well as team confidence. This activity was instrumental in reducing staff reluctance to perform high-risk challenges in the ambulatory setting, thus lifting an important barrier for the implementation of oral immunotherapy at our adult center.

### A53

Abstract withdrawn

### A54 Extended analysis of parental confidence in recognizing anaphylaxis and using the epinephrine autoinjector during oral food challenges

#### Nicole Lee^1,2^, Lianne Soller^1,2^, Timothy Teoh^2^, Ingrid Baerg^2^, Lindsay Yaworski^2^, Tiffany Wong^1,2^, Kyla J. Hildebrand^1,2^, Victoria E. Cook^1,2^, Catherine Biggs^1,2^, Scott B. Cameron^1^, Edmond S. Chan^1,2^

##### ^1^Division of Allergy and Immunology, Department of Pediatrics, Faculty of Medicine, UBC, Vancouver, BC, Canada; ^2^ Allergy Clinic, BC Children’s Hospital, Vancouver, BC, Canada

**Correspondence:** Nicole Lee

*Allergy, Asthma and Clinical Immunology* 2019, **15(Suppl 1):**A54

**Background:** Administration of intramuscular epinephrine is the first-line treatment for food-induced anaphylaxis. Patients are prescribed an epinephrine autoinjector (EAI) at the time of food allergy diagnosis. However, a prescription does not guarantee availability, and even when the device is available, it’s often not used. We previously reported improved confidence after medical supervision of parent/child autoinjector administration during OFCs. We sought to confirm those findings in a larger cohort.

**Methods:** Parents of children undergoing OFCs at the BC Children’s Hospital (BCCH) Allergy Clinic (2014–17) were asked to administer the EAI under supervision of a nurse/allergist if anaphylaxis occurred. A pre-challenge questionnaire asked about demographic/clinical characteristics, and baseline confidence in four domains: (1) ability to recognize anaphylaxis, (2) EAI administration, (3) knowledge of EAI technique, and (4) skill in EAI use. If anaphylaxis occurred, the parent/child administered the EAI, and a post-challenge questionnaire was completed. Confidence levels were evaluated on a 5-point scale (1 = not very confident to 5 = very confident). Means and 95% confidence intervals (CI) were calculated. Multivariate logistic regression was performed to determine predictors of increased confidence for the four domains.

**Results:** Of 329 families approached, 317 consented (96.4% response rate) and 353 OFCs were performed. 115 (32.6%) challenges failed, of which 53 (46.1%) required epinephrine (15% of OFCs). We confirmed a significantly positive impact in all four confidence domains. Those who reported greater knowledge in EAI technique and skill in EAI use were more likely to have older children (OR: 1.23, 95% CI 1.02, 1.47, and OR: 1.17, 95% CI 1.00, 1.37, respectively). There was no difference in confidence for other predictor variables.

**Conclusions:** Our study confirmed in a larger cohort that administering epinephrine under medical supervision during OFC has a positive effect on the four confidence domains measured. Therefore, our allergy clinic has implemented self-injection of epinephrine using autoinjectors as part of routine clinical practice.

### A55 Canadian parents’ barriers to participating in oral food challenges (OFC)

#### Elaine Hsu^1^, Lianne Soller^1^, Christopher Mill^1^, Elissa M. Abrams^2^ and Edmond S. Chan^1^

##### ^1^Division of Allergy and Immunology, Department of Pediatrics, Faculty of Medicine, University of British Columbia, BC Children’s Hospital, Vancouver, British Columbia, Canada; ^2^Department of Pediatrics, Section of Allergy and Clinical Immunology, University of Manitoba, Winnipeg, Manitoba, Canada

**Correspondence:** Elaine Hsu

*Allergy, Asthma and Clinical Immunology* 2019, **15(Suppl 1):**A55

**Background:** OFCs, considered the gold standard to diagnose food allergy, are routinely not performed for reasons which have not been well-studied. We explored parental attitudes to find out the barriers and solutions to participating in OFCs.

**Methods:** In 2017, surveys were sent to BC Children’s Hospital (BCCH) Allergy Clinic parents, and three focus groups were held at BCCH. Parents were asked about their experiences with OFCs, barriers preventing them from participating, and solutions to these barriers.

**Results:** 110 of 200 parents completed the survey (55% response rate). Top barriers included concern over serious reactions during OFCs (chosen as “moderately to extremely influential” by 62% of parents), concern over child anxiety (60%), and child fear in participating in OFCs (58%). Top solutions included performing OFCs in hospital (versus community clinics) in case of severe reaction (91%), an in-depth guide for families on how OFCs are conducted (85%), and allergist-led information sessions discussing OFC risks and benefits (84%). Twenty-seven parents attended focus groups (76% female, 89% post-secondary educated, 70% Caucasian, mean age 44 years). Additional barriers were poor experiences with previous OFCs, concern over losing their child’s trust, low impact of one OFC for multiple food allergy, and lack of OFC statistics/success stories. Top solutions included giving children control over when to stop the OFC, pre/post-OFC psychosocial support, practical support including food dosages and recipes, and discussing benefits of failed OFCs.

**Conclusions:** This is the first Canadian study to examine barriers and solutions to participating in OFCs from the parent perspective. Our study suggests that providing better OFC information to families, having psychosocial resources available, and creating dedicated OFC centres in hospitals could increase willingness of Canadian families to participate in OFCs. A limitation was all participants were BCCH patients or members of a local support group; they may not be representative of allergy families.

### A56 Skin prick test in milk allergic patients undergoing oral immunotherapy

#### Sofianne Gabrielli^1^, Bruce Thomas Miles^1^, Alexandra Langlois^1^, Bahar Torabi^2^, Duncan Lejtenyi^1^, Ann E. Clarke^3^, Bruce Mazer^1^, Moshe Ben-Shoshan^1^

##### ^1^Division of Allergy and Clinical Immunology, Department of Pediatrics, Montreal Children’s Hospital, McGill University Health Centre, Montreal, QC, Canada; ^2^The Research Institute of the McGill University Health Centre, Division of Pediatric Allergy and Clinical Immunology, Department of Pediatrics, Montreal Children’s Hospital, Montreal, QC, Canada; ^3^Division of Rheumatology, Department of Medicine, University of Calgary, Calgary, AB, Canada

**Correspondence:** Sofianne Gabrielli

*Allergy, Asthma and Clinical Immunology* 2019, **15(Suppl 1):**A56

**Background:** Skin prick tests (SPT)s are useful in the diagnosis of food allergy, however there are few studies which evaluate SPT results defining desensitization achieved with oral immunotherapy (OIT) [1]. Our objective was to assess the accuracy of SPTs with extract, diluted, and whole milk to detect desensitization in children with cow’s milk allergy undergoing OIT.

**Methods:** Children with milk allergy were recruited at the Montreal Children’s Hospital and consenting participants were blindly randomized into the OIT group vs. avoidance group. Children in the active arm received weekly increases in doses until 200 mL of milk was ingested daily. Paired t-test was used to compare SPT wheal diameter [vs. negative control with glycerinated phenol-sodium chloride 0. 9% 1:1 (ALK)] for cow’s milk extract (Omega), diluted 2% cow’s milk (1:10), and undiluted cow’s milk at study entry and when challenge with 200 mL was tolerated. SPTs for controls were conducted at study entry and 1 year later.

**Results:** Among 30 children undergoing OIT, median age was 12.5 years (IQR, 10.0, 15.0) and 50.0% were males. The differences in wheal size from baseline to 200 mL were 4.0 mm (95% CI 2.2, 5.8) for milk extract, 5.5 mm (95% CI 3.7, 7.3) for diluted milk, and 6.4 mm (95% CI 3.9, 8.9) for whole milk (attained after an average of 7.4 months). Average SPT at desensitization was negative for only diluted milk at 200 mL (< 3.0 mm). Among 12 allergic controls, median age was 14.5 years (IQR, 9.0, 16.5) and 66.7% were males. Changes in SPT size for extract, diluted and undiluted milk were not substantial [(0.58 mm (95% CI − 2.3, 3.5), 2.1 mm (95% CI − 2.0, 6.1, and 2.8 mm (95% CI − 2.4, 7.9) respectively].

**Conclusion:** Changes in clinical reactivity to cow’s milk during OIT are associated with substantial decrease in SPT size assessed by diluted, undiluted or by milk extract.

### A57 Differential profiling of small molecule distribution in raw vs. roasted peanuts using high-resolution magic angle spinning (HR-MAS) and solution ^1^H nuclear magnetic resonance (NMR) spectroscopy

#### Casey G. Cohen, Wei Zhao, Bertrand J. Jean-Claude, Bruce D. Mazer

##### Research Institute of the McGill University Health Centre, Montreal, Quebec, Canada

**Correspondence:** Casey G. Cohen

*Allergy, Asthma and Clinical Immunology* 2019, **15(Suppl 1):**A57

**Background:** Peanut allergy is considered the most severe of all food allergies as it is the leading cause of fatal anaphylaxis. An estimated 1% of the North American population suffers from peanut allergy. Evidence suggests that the allergenicity of peanut is significantly increased in its roasted form when compared with raw. Our project aims to develop methods to decrease the allergenicity of roasted peanut. We therefore undertook a nuclear magnetic resonance (NMR) study to establish small molecule signatures of mixtures derived from roasted and raw peanuts.

**Methods:** High-Resolution Magic Angle Spinning (HR-MAS) ^1^H NMR was used to take snapshots of the peanut in its solid form under both raw and roasted conditions. Peanuts were ground into a paste and dissolved in hexane to remove the lipid content, and then both untreated peanut and defatted peanut were analyzed by HR-MAS ^1^H NMR. Raw and roasted peanuts were soaked in double distilled water and the resulting solutions were characterized by ^1^H NMR.

**Results:** The results showed that differences between raw and roasted whole peanuts in the solid state could not be detected by HR-MAS NMR due to the dominance of the triglyceride peaks in the spectrum. Defatting of the peanut prior to HR-MAS NMR analysis revealed significant differences between the small molecule profiles of both raw and roasted peanut. In the peanut-soaked solutions, significant differences were also observed in the sugar pattern, with sucrose clearly being the dominant species in the raw peanut, but with glucose being dominant in the roasted peanut.

**Conclusion:** The results suggest that NMR spectroscopy, with both the solid and liquid states, is a useful tool for determining small molecule profile differences between the two levels of allergic states of the peanut.

### A58 Sensitivity and specificity of a generalized anxiety tool to measure food allergy associated anxiety

#### Lianne Soller^1,2^, Sharon To^3^, Theresa Newlove^3^, Edmond S. Chan^1,2^

##### ^1^Division of Allergy and Immunology, Department of Pediatrics, Faculty of Medicine, UBC, Vancouver, BC, Canada; ^2^Allergy Clinic, BC Children’s Hospital, Vancouver, BC, Canada; ^3^Department of Psychology, BC Children’s Hospital, Vancouver, BC, Canada

**Correspondence:** Lianne Soller

*Allergy, Asthma and Clinical Immunology* 2019, **15(Suppl 1):**A58

**Background:** While pediatric allergists describe that families experience significant anxiety and difficulty coping with living with food allergy (FA), no instruments exist that can screen and measure parental anxiety specific to FA. Instead, researchers use tools that measure generalized anxiety. This study sought to measure anxiety in Canadian parents of children with FA, and compare tools measuring generalized anxiety to self-reported FA-associated anxiety.

**Methods:** Parents were invited, through Food Allergy Canada, to participate in an online survey about their generalized anxiety using State Trait Anxiety Inventory (STAI) and FA-specific anxiety using a visual analogue scale (VAS). We used a cut-off of 47 for high anxiety for state and trait anxiety based on the STAI manual [1], and a cut-off of 92.7, one-standard deviation above the mean for FA-specific anxiety using VAS. We calculated sensitivity and specificity of using STAI versus using VAS.

**Results:** Among 1244 who clicked the survey, 548 completed it (44.1%). When parents were asked to rate (on a VAS of 0 to 100) how anxious they are about their child’s FA, the average score was 71.2. The mean State and Trait Anxiety scores were 42.0 and 41.4, respectively. Sensitivity of the STAI when State anxiety was used is 68.6% and 50.0% for Trait, and the specificity is 70.0% and 71.8% for state and trait, respectively.

**Conclusions:** Our data show that generalized anxiety questionnaires like the STAI don’t adequately identify parents who are anxious about their child’s FA as measured by VAS, having classified 31–50% of parents who report being anxious about FA as not anxious. Therefore, an anxiety diagnostic tool specific to FA is warranted. Our future research will develop a diagnostic tool validated against clinical criteria that can be used by allergists to quickly identify anxious parents and provide resources to cope with having a child with FA.

**Acknowledgements:** Ranjit Dhanjal, Jennifer Gerdts, Laurie Harada of Food Allergy Canada

### A59 The peculiar biomedical puzzle of immunotherapy (IT) for food allergy (FA)

#### Stephanie A. Nair

##### McGill University, Department of Sociology and Social Studies of Medicine, Montreal Quebec, Canada

**Correspondence:** Stephanie A. Nair

*Allergy, Asthma and Clinical Immunology* 2019, **15(Suppl 1):**A59

**Background:** Immunotherapy for Food Allergy has recently (since at least the beginning of the twenty-first century) become an object of increased scientific experimentation in clinical trials across North America. IT has simultaneously been integrated into clinical and private practices across Canada and the US, although the extent to which it has been adopted and performed in private or community practice by physicians, academicians and scientists is unknown. IT represents a peculiar biomedical puzzle, as contradictory definitions and understandings of what IT is, operate simultaneously across both community and private practice and in clinical trials for IT.

**Methods:** Based on in-depth, semi-structured interviews with 40 experts and innovators in the field of food allergy research and practice across North America and extensive observational/ethnographic research at clinical and scientific conferences for allergy and immunology, this paper explores the ambiguity that currently characterizes the field and highlights the multi-dimensional debate about IT for FA.

**Results:** A perceived lack of reliable diagnostic tests and procedures has amplified experts’ concerns about who is receiving or who should receive IT for FA. Relatedly, debate about whether IT for FA is a “food” or a “drug”, has diversified the understandings, practices and goals of IT for FA. Despite the desire to standardize the practice of IT and to bridge the “gaps” between academic research and routine care, contradictory and potentially incompatible understandings of IT continue to aggravate the domain of food allergy and immunology.

**Conclusion:** It is evident therefore, that the uptake of IT may depend not merely on demonstrating that IT “works” in a biomedical, immunological or clinical sense but relatedly also on reconciling the ontological and epistemological ambiguities and uncertainties that pervade FA in both research and clinical/private practice.

### A60 Occurrence of food allergen in products with precautionary labeling in Canada

#### Emilie Manny^1,2^, Virginie Barrere^1,2^, Jérémie Théolier^1,2^, Elise Fortin^1^, Stéphanie Parent^1^, Sébastien La Vieille^1^ & Samuel Godefroy^1,2^

##### ^1^Faculty of Food and Agricultural Sciences (FSAA), Université Laval, Quebec, Quebec, Canada; ^2^Institute of Nutrition and Functional Foods (INAF), Université Laval, Quebec, Quebec, Canada

**Correspondence:** Emilie Manny

*Allergy, Asthma and Clinical Immunology* 2019, **15(Suppl 1):**A60

**Background:** The use of precautionary allergen labeling (PAL) to indicate the potential presence of priority food allergens as a result of cross-contamination is increasing [1]. These statements are often overused by industries and misunderstood by consumers and can lead allergic patients to adopt risky behaviors, resulting in potential adverse reactions [2]. The aim of this study is to quantify for the first time the risk for Canadian allergic individuals exposed to pre-packaged products that may contain allergens due to cross-contamination.

**Methods:** Foodstuffs with PAL have been analysed for the possible presence of milk, eggs and peanuts. Products were chosen according to a sampling plan based on the recalls done by the Canadian Food Inspection Agency (CFIA) from 1997 to 2017. Sandwich ELISA kits from r-biopharm and Morinaga were used for allergens’ detection and quantification. For each food product, the allergic risk associated with its consumption will be estimated using the “consumption estimates per eating occasion” data held in the Canadian Community Health Survey (CCHS-2015). The occurrence of adventitious allergens in foods and the dose–response relationship will be estimated with a probabilistic approach like those already published in the literature [3].

**Results:** Three allergens have been investigated (milk = 253, eggs = 91, peanuts = 48). Considering all food product categories and the allergens together, only 17% of the products with PAL had detectable amounts of allergens. As an example, dark chocolate has the highest occurrence of milk with 88% of positive products, with a milk protein content range of 10.5–6231 ppm (mg/kg). Besides, no consistency was shown between the different lots (n = 5).

**Conclusion:** More data must be acquired to undergo risk assessments on the probability of occurrence of allergic reactions in Canadian allergic individuals consuming products with PALs. Allergen management guidelines linked to industry and regulation stakeholders will be proposed following the results of the risk assessments.

**Acknowledgements:** This research study is financially supported by AllerGen. The authors are thankful to the Canadian Food Inspection Agency (CFIA) for providing guidance and training, and r-biopharm and Morinaga for their support.

## OTHER ALLERGY/IMMUNOLOGY

### A61 Review of the Manitoba cohort of patients with hereditary angioedema with normal C1 inhibitor

#### Lundy M^c^Kibbin, Colin Barber, Chrystyna Kalicinsky

##### Rady Faculty of Medicine, Department of Internal Medicine, Section of Allergy and Clinical Immunology, University of Manitoba, Winnipeg, Manitoba, Canada

**Correspondence:** Lundy M^c^Kibbin

*Allergy, Asthma and Clinical Immunology* 2019, **15(Suppl 1):**A61

**Background:** Hereditary angioedema with normal C1 inhibitor (HAE‐nC1 INH) is a rare, underappreciated condition characterized by recurrent subcutaneous angioedema^1^. The underlying pathophysiology and diagnostic criteria continues to evolve. There is a significant overlap between HAE –nC1 INH and idiopathic nonhistaminergic angioedema, and this may ultimately be found to be the same condition. Characterization of cohorts suspected to have these conditions is warranted to help refine diagnosis, pathophysiology, and treatment response.

**Methods:** A retrospective chart review of 418 charts of patients diagnosed with angioedema was conducted. The following inclusion criteria were used: lack of response to antihistamines, steroids, and epinephrine; normal C4, C1 INH level and function; lack of urticaria or pruritus; occurrence without offending drugs; and family history. Charts meeting these criteria were reviewed for frequency and type of episodes as well as use and response to therapies.

**Results:** 6 patients met the above criteria. 3 underwent genetic testing and none were found to have factor XII abnormalities. None had angiopoietin 1 or plasmin testing^2^. 5 of 6 patients were successfully treated with other regimens for acute treatment of attacks (4 with C1 INH and 1 with Tranexamic acid). 4 patients have used Icatibant with good response (typically under 40 min for near full recovery); of these, 3 required Icatibant as acute treatment after other therapies were ineffective. There were 9 patients who otherwise met criteria, but due to a lack of family history were classified as having idiopathic non-histaminergic angioedema.

**Conclusions:** This retrospective chart review found 6 HAE‐nC1 INH patients in Manitoba. 1 responded to Tranexamic acid and not C1 INH, 4 typically responded to C1 INH, and 1 responded exclusively to Icatibant. All patients—4 total—who used Icatibant responded; of the 4, 3 used Icatibant after other therapies had failed.

### A62

Abstract withdrawn

### A63 Lanadelumab Inhibition of plasma kallikrein activity for effective HAE prophylaxis

#### R. Gagnon^1^, M. Cicardi^2^, M. Shennak^3^, R. Zaragoza-Urdaz^4^, J. F. Marier^5^, P. Lu^6^, D. Sexton^6^, R. Faucette^6^, J. Kenniston^6^, H. Rong^6^, Y. Wang^6^, W. H. Yang^7^, J. Hébert^1^

##### ^1^Centre de Recherche Appliquée en Allergie de Québec, Québec City, QC, Canada; ^2^Department of Biomedical and Clinical Sciences, Luigi Sacco Hospital, University of Milan, ASST Fatebenefratelli Sacco, Milan, Italy; ^3^Triumpharma, Amman, Jordan; ^4^University of Puerto Rico School of Medicine, San Juan, Puerto Rico; ^5^Certara Strategic Consulting, Montreal, QC, Canada; ^6^Shire, Cambridge, MA, USA; ^7^Ottawa Allergy Research Corporation and University of Ottawa Medical School, Ottawa, ON, Canada

**Correspondence:** R. Gagnon

*Allergy, Asthma and Clinical Immunology* 2019, **15(Suppl 1):**A63

**Background:** Hereditary angioedema (HAE) results from a mutation in the C1 esterase inhibitor (C1-INH) gene. Deficiencies in C1-INH result in uncontrolled plasma kallikrein activity, which is associated with HAE attacks. Lanadelumab binds specifically to active plasma kallikrein (Ki = 125 pM) and was efficacious in the prevention of HAE attacks in the phase 3 HELP study. Here we evaluate the dosing of lanadelumab required to achieve stable plasma kallikrein inhibition at levels sufficient to prevent HAE attacks.

**Methods:** Patients with type I/II HAE received placebo or lanadelumab (150 mg q4wks, 300 mg q4wks, or 300 mg q2wks) over 26 weeks in the HELP study. Blood samples were collected pre-dose and at intervals up to 26 weeks for measurement of lanadelumab and cleaved high molecular weight kininogen (cHMWK), a pharmacodynamic marker of plasma kallikrein activity.

**Results:** Plasma lanadelumab concentrations reached steady state at ~ week 10, with a half-life of ~ 14 days. Compared with healthy subjects, plasma kallikrein levels were estimated based on previous reports to be elevated in HAE plasma between attacks (by 10–276 nM) and during attacks (an additional 20–186 nM). Following lanadelumab treatment, plasma kallikrein activity (cHMWK levels) decreased in a concentration-dependent manner, reaching a 44.7% reduction from baseline by week 8 with 300 mg q2wks lanadelumab, but remained elevated in the placebo group. Mean (± SE) maximum inhibition (I_max_) was 53.7 ± 5.9%, and IC_50_ (concentration at 50% of I_max_) was 5705 ± 13.9 ng/mL. 300 mg q2wks lanadelumab achieved steady state average plasma concentrations (29,200 ng/mL) more than fivefold above the IC_50_. Over 26 weeks, patients receiving lanadelumab had fewer attacks/month compared with those receiving placebo (0.31–0.48 vs 2.46 attacks/month, respectively).

**Conclusions:** Lanadelumab resulted in a marked suppression of kallikrein activity at drug levels approximately equimolar to the amount of protease, resulting in sufficient inhibition for effective HAE prophylaxis.

### A64 Efficacy of lanadelumab in the Phase 3 HELP study: Exploratory analyses based on prior disease activity and prior use of C1-INH long term prophylaxis therapy

#### William H. Yang^1^, John T. Anderson^2^, Paula Busse^3^, Marco Cicardi^4^, Mark Davis-Lorton^5^, Douglas T. Johnston^6^, Peng Lu^7^, Christina Nurse^7^, Marcus Maurer^8^

##### ^1^Ottawa Allergy Research Corporation and University of Ottawa Medical School, Ottawa, ON, Canada; ^2^Alabama Allergy & Asthma Center, Birmingham, AL, USA; ^3^Division of Allergy and Clinical Immunology, Icahn School of Medicine at Mount Sinai, New York, NY, USA; ^4^Department of Biomedical and Clinical Sciences, Luigi Sacco Hospital, University of Milan, ASST Fatebenefratelli Sacco, Milan, Italy; ^5^ENT and Allergy Associates, Sleepy Hollow, NY, USA; ^6^Clinical Research of Charlotte, Charlotte, NC, USA; ^7^Shire, Lexington, MA, USA; ^8^Department of Dermatology and Allergy, Allergie-Centrum-Charité, Charité–Universitätsmedizin Berlin, Berlin, Germany

**Correspondence:** William H. Yang

*Allergy, Asthma and Clinical Immunology* 2019, **15(Suppl 1):**A64

**Background:** The HELP Study evaluated the efficacy and safety of lanadelumab for long-term prophylaxis (LTP) in patients ≥ 12 years old with HAE type I/II (NCT02586805). Here, we report lanadelumab efficacy based on a patient’s prior disease activity and prior use of C1-INH LTP.

**Methods:** The HELP study is a phase 3, randomized, double-blind, placebo-controlled study. We performed two exploratory efficacy analyses: (1) a responder analysis comparing normalized HAE attack rates over 26 weeks of treatment to a 4–8 week run-in period prior to treatment with lanadelumab and (2) a Poisson regression model to compare the mean HAE attack rate in the lanadelumab groups to placebo by patient prior C1-INH LTP use.

**Results:** Over the 26-week treatment period, the percentage of patients with a ≥ 50%, ≥ 70%, and ≥ 90% reduction in investigator-confirmed HAE attacks from the run-in period, respectively, was 89.3%, 78.6% and 64.3%, [lanadelumab 150 mg q4wks (n = 28)]; 100%, 75.9%, 55.2% [lanadelumab 300 mg q4wks (n = 29)]; 100%, 88.9% and 66.7% [lanadelumab 300 mg q2wks (n = 27)] and 31.7%, 9.8% 4.9% [placebo (n = 41)], respectively. In C1-INH LTP patients (n = 60), the attack rate was significantly reduced in all lanadelumab groups versus placebo (P < 0.001); the reduction was similar in magnitude to those who did not receive prior LTP (n = 55). For the lanadelumab 150 mg q4wks, 300 mg q4wks, 300 mg q2wks and placebo groups, respectively, C1-INH LTP users reported mean monthly attack rates (3 months prior to the study) of 3.0, 2.7, 2.6 and 4.0; during run-in 3.3, 3.7, 4.6 and 4.6; and during the treatment period 0.5, 0.7, 0.5 and 2.9.

**Conclusions:** Treatment with lanadelumab for 26 weeks resulted in a high rate of patients who experienced a clinically meaningful reduction in investigator-confirmed HAE attacks compared to baseline run-in. Furthermore, all lanadelumab doses significantly reduced attack rates versus placebo, regardless of whether patients had received prior C1-INH LTP.

### A65 Alterations in cord blood hemopoietic progenitor cell surface receptor expression precede atopy and poor lung function at 1- and 3-years in the Canadian Healthy Infant Longitudinal Development Study

#### Loubna Akhabir^1^, Elli Rosenberg^1^, Delia Heroux^1^, Jyoti Balhara^1^, Padmaja Subbarao^2^, Kelly McNagny^3^, Malcolm Sears^1^, Diana L. Lefebvre^1^, Judah A. Denburg^1^

##### ^1^Department of Medicine, McMaster University, Hamilton, ON, Canada; ^2^Department of Pediatrics, Hospital for Sick Children, University of Toronto, Toronto, ON, Canada; ^3^Biomedical Research Centre, The University of British Columbia, Vancouver, BC, Canada

**Correspondence:** Loubna Akhabir and Elli Rosenberg

*Allergy, Asthma and Clinical Immunology* 2019, **15(Suppl 1):**A65

**Background:** Hemopoietic progenitor cells (HPC), both in the bone marrow and in peripheral tissues, differentiate towards inflammatory effector cells and, thus, can modulate central and peripheral inflammation. There is growing evidence for the involvement of hemopoietic processes in the pathogenesis of atopy and asthma from pre-conception and birth. This is the basis for the “bone marrow” hypothesis of allergic disease, arguing that a perinatal environmental challenge leads to the skewed production and mobilization of HPC, regulating central and peripheral production of cell types that perpetuate allergic responses. The objective of this study was to assess the association of cell surface receptor profiles of cord blood (CB) HPC with atopy development and lung function at 1- and 3-years in the Canadian Healthy Infant Longitudinal Development (CHILD) Study

**Methods:** We used six-colour flow cytometry to assess cytokine and toll-like receptor expression levels in CB HPC from infants with atopy data (defined as positive skin prick test and atopic dermatitis and wheeze) and lung function data (by lung clearance index (LCI)) at 1- and 3-years of age in CHILD.

**Results:** We found a significant increase in IL5R and IL17RB-expressing HPC populations in the CB of children atopic at 1-year. Conversely, GM-CSFR and ST2-expressing CB HPC were decreased in atopic children both at 1- and 3-years. The expression levels of IL17RB on the surface of CB-HPC were higher in atopics at 3-years. Finally, infants with poor lung function at 3-years exhibited higher IL5R expression on the surface of CB HPC.

**Conclusion:** This study provides evidence of pre-existing cellular alteration in the infants’ CB progenitors at birth, which antedate development of atopy/allergic disease and potentially future asthma. Our results can contribute to novel strategies for atopic/allergic disease interception in infants before onset, and hence participate in the health and well-being of Canadian children.

### A66 Efficacy and safety of lanadelumab for prevention of hereditary angioedema attacks: results from the phase 3 HELP Study

#### Jacques Hébert^1^, Aleena Banerji^2^, Jonathan A. Bernstein^3^, Paula J. Busse^4^, Marco Cicardi^5^, Mark Davis-Lorton^6,7^, Selina Gierer^8^, Hilary J. Longhurst^9,10^, Marcus Maurer^11^, Marc Riedl^12^, Raffi Tachdjian^13^, Bruce Zuraw^12^, Peng Lu^14^, James Hao^14^, and William H. Yang^15^

##### ^1^Centre de Recherche Appliqué en Allergie de Québec, Quebec, QC Canada; ^2^Division of Rheumatology, Allergy and Immunology, Department of Medicine, Massachusetts General Hospital, Harvard Medical School, Boston, MA, USA; ^3^Department of Internal Medicine/Allergy Section, University of Cincinnati Medical Center, Cincinnati, OH, USA; ^4^Icahn School of Medicine at Mount Sinai, New York, NY, USA; ^5^Department of Biomedical and Clinical Sciences, Luigi Sacco Hospital, University of Milan, Milan, Italy; ASST Fatebenefratelli Sacco, Milan, Italy; ^6^Rheumatology Allergy and Immunology, NYU Winthrop Hospital, Mineola, NY, USA; ^7^ENT and Allergy Associates, Sleepy Hollow, NY, USA; ^8^Division of Allergy, Clinical Immunology & Rheumatology, University of Kansas Medical Center, Kansas City, KS, USA; ^9^Department of Immunology, Barts Health NHS Trust, London, UK; ^10^Addenbrooke’s Hospital, Cambridge University Hospitals NHS Foundation Trust, Cambridge, UK; ^11^Department of Dermatology and Allergy, Allergie-Centrum-Charité, Charité-Universitätsmedizin Berlin, Berlin, Germany; ^12^Division of Rheumatology, Allergy and Immunology, University of California-San Diego School of Medicine, La Jolla, CA, USA; ^13^AIRE Medical of Los Angeles, University of California Los Angeles, Los Angeles, CA, USA; ^14^Shire, Lexington, MA, USA; ^15^Ottawa Allergy Research Corporation and University of Ottawa Medical School, Ottawa, ON, Canada

**Correspondence:** Jacques Hébert

*Allergy, Asthma and Clinical Immunology* 2019, **15(Suppl 1):**A66

**Background:** The efficacy and safety of lanadelumab, a fully human monoclonal antibody inhibitor of plasma kallikrein, in preventing hereditary angioedema (HAE) attacks were evaluated.

**Methods:** In the randomized, double-blind, placebo-controlled, parallel arm, multi-center phase 3 HELP Study (NCT02586805), patients ≥ 12 years old with HAE type I/II and ≥ 1 attack during a 4-week run-in period received subcutaneous injections of placebo or lanadelumab (150 mg q4wks, 300 mg q4wks, or 300 mg q2wks) for 26 weeks (days 0–182).

**Results:** Of 125 enrolled patients (mean age 40.7 yrs; 70.4% female; 90.4% Caucasian); 113 completed the study. Over 26 weeks, lanadelumab reduced the number of attacks versus placebo by 75.6%, 73.3%, and 86.9% in the 150 mg q4wks, 300 mg q4wks, and 300 mg q2wks groups, respectively; all adjusted p < 0.001. The attack rate compared with run-in was reduced by ≥ 50% in 89.3%, 100%, and 100% of patients in the lanadelumab groups, respectively (31.7% placebo). The rate of moderate/severe attacks was reduced by up to 83.4% versus placebo, and up to 44.4% of lanadelumab-treated patients (2.4% placebo) were attack-free. During steady state (days 70–182), the efficacy of lanadelumab was more pronounced: the number of attacks versus placebo was reduced by 77.6%, 80.6%, and 91.5%, respectively; the rate of moderate/severe attacks was reduced by up to 88.4% versus placebo; and up to 76.9% of lanadelumab-treated patients were attack-free (2.7% placebo). There were no treatment-related serious AEs or deaths. The most common AEs were injection site reactions. Most AEs were mild to moderate in severity.

**Conclusions:** Lanadelumab significantly reduced the number of attacks and number of moderate/severe attacks over 26 weeks versus placebo. Most patients experienced an attack rate reduction of ≥ 50%. The benefit of lanadelumab was optimal during steady state. Lanadelumab was generally safe and well tolerated.

**Acknowledgements:** Presented on behalf of the HELP Study investigators.

### A67 Maternal report of in-home occurrence of environmental exposures during pregnancy and allergic outcomes of their children at 2 years of age

#### Mallory Gallant^1^, Wilma Hopman^2^, Michelle North^3^, Lisa Steacy^3^, Anne K. Ellis^1,3^

##### ^1^Departments of Medicine and Biomedical & Molecular Sciences, Queen’s University, Kingston, ON, Canada; ^2^Kingston General Health Research Institute, Kingston, ON Canada; ^3^Allergy Research Unit, Kingston Health Sciences Centre-Kingston General Hospital site, Kingston, ON, Canada

**Correspondence:** Mallory Gallant

*Allergy, Asthma and Clinical Immunology* 2019, **15(Suppl 1):**A67

**Background:** Previous work has indicated that maternal environmental exposures during pregnancy can influence allergic disease progression in her offspring. Some exposures may be protective or harmful. Although these exposures rarely occur in singularity, the combined effect of multiple exposures on allergy remains unclear. We investigated maternal prenatal exposure to seven factors and determined their influence on allergy in the children at 2 years of age.

**Methods:** Prior to delivery, consenting pregnant women (n = 92) completed a survey regarding their home environment during pregnancy. Data on the presence of dogs, cats, mold, carpets, air fresheners, candles/incense, and cigarette smoke during pregnancy was captured for each participant. Children completed allergy testing at 2 years of age via skin prick testing (SPT). Mother’s allergic status was determined at least 6 months post-delivery. Statistical analysis was performed using GraphPad Prism 7.

**Results:** 74 children had a negative SPT and 18 had a positive SPT at 2 years of age. All prenatal exposures except for carpet increased the odds ratio (OR) of a child’s positive SPT; however, candles/incense was the only exposure that was statistically significant (OR: 5.1, 95% confidence interval 1.7–13.9 p = 0.006). The median total number of exposures for children with a positive SPT was greater when compared to their negative SPT counterparts (4 vs 2 p = 0.005). All children with 0 or 1 exposure had a negative SPT and as the total number of exposures increased, the percentage of SPT positive and SPT negative children increased and decreased respectively (p < 0.001).

**Conclusion:** We have provided additional evidence supporting the influence of prenatal exposures on childhood allergy. The relationship between increasing total exposure number and increasing percentages of positive SPT is important as it suggests that the combined effects of multiple exposures may be more influential on allergy development than one single exposure.

### A68 Triggers, pharmacologic treatments, and complementary-alternative medicine use in patients with chronic urticaria

#### Hoang Pham, Manstein Kan, Amin Kanani

##### Department of Medicine, Division of Allergy and Clinical Immunology, University of British Columbia, Vancouver, British Columbia, Canada

**Correspondence:** Hoang Pham

*Allergy, Asthma and Clinical Immunology* 2019, **15(Suppl 1):**A68

**Background:** The natural history of chronic urticaria is limited, especially in the Canadian context. The purpose of this study is to determine the triggers and various treatments adopted by patients—including complementary/alternative medicine (CAM) and acute emergency services—from a single center in Vancouver, BC.

**Methods:** This is a prospective cross-sectional, single centered study in Canada, which involved a review of participant’s medical records and a participant survey to evaluate basic demographics, triggers, and therapies used by individuals with chronic urticaria.

**Results:** 72 participants completed the survey with 59 females (82%). Patient ethnicities included 39 Caucasians (54%), 25 Asians (35%), 5 Middle Easterners (7%), 1 Hispanic (1%), and 2 First Nations (3%). The mean age of onset was 43 ± 17 years old. Patient perceived triggers included scratching 47%, stress 38%, heat 35%, cold 14%, food 11%, NSAIDS 11%, exercise 10%, alcohol 7%, and sunlight 3%. Sleep was affected in 75%. Treatments used and patient reported benefit (in parentheses) included first generation antihistamines 76% (78%), second generation antihistamines 97% (81%), prednisone 39% (75%), omalizumab 21% (73%), cyclosporine 4% (100%), montelukast 3% (0%), IVIG 1% (0%), methotrexate 1% (100%), naturopathy 21% (27%), acupuncture 7% (20%), and traditional Chinese medicine 3% (28%). Emergency department visits were reported by 43% of the participants.

**Conclusions:** This study observed a higher than expected proportion of females compared to males. Evidenced-based therapies such as omalizumab and cyclosporine may need more knowledge translation while unproven CAM therapies should be discouraged. Better understanding of the natural history, treatments, and outcomes in chronic urticaria will allow physicians to better inform patients and reduce emergency department visits.

### A69 Assessing risk factors associated with a positive graded oral challenge (GOC) in children with suspected amoxicillin allergy

#### Rutherford Exius^1^, Sofianne Gabrielli^2,4^, Christine McCusker^1,2,3,4^, Moshe Ben-Shoshan^1,2,3,4^

##### ^1^Division of Clinical Epidemiology, Department of Medicine, McGill University Health Center, Montreal, Quebec, Canada; ^2^Division of Pediatric Allergy and Clinical Immunology, Department of Pediatrics, Montreal Children’s Hospital, McGill University Health Center, Montreal, Quebec, Canada; ^3^Division of Dermatology, Department of Medicine, McGill University Health Center, Montreal, Quebec, Canada; ^4^Division of Experimental Medicine, The Research Institute of the McGill University Health Centre, McGill University, Montreal, Quebec, Canada

**Correspondence:** Rutherford Exius

*Allergy, Asthma and Clinical Immunology* 2019, **15(Suppl 1):**A69

**Background:** The diagnosis of amoxicillin hypersensitivity is challenging in children given that the sensitivity of commercially available skin tests is low and oral challenges are often required to establish the diagnosis. We aimed to assess the diagnostic properties of a graded oral challenge (GOC) among children presenting with a rash during amoxicillin and determine risk factors associated with positives challenges

**Methods:** A cohort study was conducted between May 1, 2013, and June 7, 2018, at the allergy clinic of the Montreal Children’s Hospital. All children referred with suspected allergy to amoxicillin were recruited and a GOC was conducted. Eligible children were followed up to assess reactions to subsequent use of amoxicillin. Data were collected on demographics clinical characteristics of reaction and comorbidities.

**Results:** 1478 children were assessed (median age, 1.7 years [interquartile range, 1.0–3.5 years]; 745 [53.5%] male). 1405 (95.1%) tolerated the GOC, 28 (1.9%) developed mild immediate reactions, and 45 (3.0%) developed nonimmediate reactions. The GOC had a specificity of 100.0% (95% CI 90.9%–100.0%), a negative predictive value of 97.1%(95% CI 93.5%–98.8%), and a positive predictive value of 100.0% (95% CI 93.8%–100.0%). Among all 728 participants eligible for annual follow-up, 679 (93.3%; 95% CI 91.1%–94.9%) responded, 227 of whom received subsequent full treatment with amoxicillin; 201 of these 227 participants (88.5%) reported tolerance, while 26 (11.5%) had mild to severe reactions. History of a reaction occurring within 5 min of exposure was associated with immediate reactions to the GOC (adjusted Odds Ratio [aOR] = 1.17; 95% CI 1.11–1.23) A rash lasting longer than 7 days (aOR = 1.09; 95% CI 1.02–1.17) was associated with nonimmediate reactions and with reaction under subsequent full treatment (aOR = 1.15; 95% CI 1.01–1.31).

**Conclusions:** GOCs provide an accurate and safe confirmatory test for skin-related reactions to amoxicillin. Further studies are required to assess factors associated with the GOC outcome groups.

### A70 The use of acid-suppressive medications during infancy is associated with an ‘allergic’ shift in gut microbiota composition

#### Kelsea M. Drall^1^, Amanda A. Lau^1^, Hein M. Tun^1^, Hien Q. Huynh^1^, David S. Guttman^2^, Malcolm R. Sears^3^, Puishkumar J. Mandhane^1^, Padmaja Subbarao^4^, Stuart E. Turvey^5^, Allan B. Becker^6^, Diana L. Lefebvre^3^, James A. Scott^7^, Anita L. Kozyrskyj^1^ and the CHILD Study Investigators

##### ^1^Department of Pediatrics, University of Alberta, Edmonton, Alberta, Canada; ^2^Centre for the Analysis of Genome Evolution and Function, University of Toronto, Toronto, Ontario, Canada; ^3^Department of Medicine, McMaster University, Hamilton, Ontario, Canada; ^4^Department of Pediatrics, University of Toronto, Toronto, Ontario, Canada; ^5^Department of Pediatrics, University of British Columbia, Vancouver, BC, Canada; ^6^Department of Pediatrics and Child Health, University of Manitoba, Winnipeg, Manitoba, Canada; ^7^Dalla Lana School of Public Health, University of Toronto, Toronto, Ontario, Canada

**Correspondence:** Kelsea M. Drall

*Allergy, Asthma and Clinical Immunology* 2019, **15(Suppl 1):**A70

**Background:** The use of acid-suppressive medications during infancy is consistently linked to the development of allergic disease [1, 2]. The mechanism of this association remains unknown, but disturbances to the gut microbiota may play a role as intestinal bacteria are pH sensitive. This study aims to explore the relationship between the use of proton pump inhibitors (PPIs) or histamine-2 receptor antagonists (H2RAs) and the fecal microbiota of infants with gastroesophageal reflux disease (GERD).

**Methods:** Drug use was reported in a medication questionnaire completed at 3 months postpartum for infants in the Canadian Healthy Infant Longitudinal Development (CHILD) cohort (N = 3455). A subset of 1028 infants with a completed medication questionnaire and fecal sample collected at 3 months were included in this study. Samples were profiled using 16S rRNA sequencing and quantified using qPCR. Kruskal–Wallis tests were used to compare the median relative abundances of taxa and the Chao1 richness and Shannon diversity indices of GERD medication users and non-users. Colonization (yes/no) was evaluated using Chi square tests.

**Results:** 5% of infants reported using an acid-suppressive medication (3.95% H2RA and 1.32% PPI). Compared to non-medicated infants, those receiving PPIs had an increased abundance of Lachnospiraceae (p = 0.05), Streptococcaceae (p = 0.01, also p = 0.02 with H2RA) and reduced Bifidobacteraceae (p = 0.07). Furthermore, 53.9% of infants receiving an H2RA were colonized *Clostridium difficile*, compared to 36.9% of non-users (p = 0.04). Overall, the microbial richness and diversity were not different between groups and findings remained largely unchanged following stratification and adjustment for feeding mode, infant use of antibiotics and indication for vomiting.

**Conclusion:** Decreased abundance of Bifidobacteraceae and colonization with *C. difficile* are characteristic of infants with an increased risk of allergy and asthma [3, 4]. Our study suggests that the use of GERD drugs during infancy is not without consequence and the impact on the gut microbiota needs to be considered when prescribing these medications.

### A71 Patient satisfaction in hereditary angioedema patients on C1 esterase inhibitor home infusion program

#### Hasandeep Kular, Amin Kanani

##### Division of Allergy and Immunology, University of British Columbia, Vancouver, British Columbia, Canada

**Correspondence:** Hasandeep Kular

*Allergy, Asthma and Clinical Immunology* 2019, **15(Suppl 1):**A71

**Background:** Hereditary angioedema (HAE) is caused by a C1 esterase inhibitor deficiency which results in acute painful swelling in the upper airway, bowel, extremities, and face [1]. Self-administration of C1 esterase inhibitor has been shown to be safe and provide improvement in quality of life [2–4]. We assessed patient satisfaction with self-administered C1 esterase inhibitor treatment.

**Methods:** HAE patients on C1 inhibitor home infusion program were identified through the Canadian Blood Services transfusion registry. Data was available for 26 patients in one health authority in British Columbia, Canada. A patient satisfaction survey was administered by the home infusion program nurse.

**Results:** 25 out of 26 identified patients participated in the interview survey.

Assessment of patients’ overall satisfaction infusing at home revealed 57% of responders reporting 5/5 satisfaction, and 22% reporting 4/5 satisfaction. Only 4% of responders reported being not at all satisfied. 28% of patients reported greatly improved length or severity of acute angioedema episodes, 19% reported somewhat improved, while 43% reported no change. Eight patients used C1 esterase inhibitor infusions as a regular prophylactic home dose. 75% of these patients reported a decreased frequency of acute episodes. 81% of responders rated self-administered C1 inhibitor as being more effective than other forms of treatment. Responders indicated that other treatments caused major side effects impacting their life, such as danazol causing unwanted hair growth, mood changes, and weight gain. In comparison, 21/22 patients reported no side effects to C1 inhibitor.

**Conclusions:** The majority of patients expressed high overall satisfaction with home infusion. Most reported increased effectiveness compared to other treatments and almost all reported no significant side effects. There was a decreased frequency of acute episodes among those using prophylactic home C1 inhibitor doses. This further supports HAE guideline recommendations for providing self-administration C1 inhibitor training [5, 6].

**Acknowledgements:** Canadian Blood Services, BC Provincial Blood Coordinating Office (PBCO), BC Clinical and Support Services Society (BCCSS), Fraser Health Authority.

### A72 Diagnosing serum sickness-like reactions using a graded oral challenge

#### Luca Delli Colli, Sofianne Gabrielli, Christine McCusker, Moshe Ben-Shoshan

##### Division of Allergy and Clinical Immunology, Department of Pediatrics, Montreal Children’s Hospital, Montreal, Quebec, Canada

**Correspondence:** Luca Delli Colli

*Allergy, Asthma and Clinical Immunology* 2019, **15(Suppl 1):**A72

**Background:** Serum sickness-like reactions (SSLR)s are defined by presence of rash, primarily hives, and joint complaints (arthralgia/arthritis) and are believed to occur due to a non-IgE mediated allergic-type response to drugs and/or acute viral infections^1^. Thus, determining the etiology of any given episode is challenging. We aim to evaluate children presenting with SSLRs associated with medication use through a graded oral challenge.

**Methods:** All children referred to the Montreal Children’s Hospital for potential antibiotic allergy and a clinical presentation compatible with SSLR were recruited for the LAACTAM study between March 2013 and June 2018. A standardized survey with questions on treatment, symptoms, and associated factors was filled and an oral challenge (10% and 90% of the oral dose) was conducted. Patients with negative challenge were contacted annually to query subsequent antibiotic use.

**Results:** Among 34 patients presenting with suspected SSLRs, the median age was 2.1 years (IQR 1.3, 4.0) and 50.0% (95% CI 34.1%, 66.0%) were males. The majority, 88.2% (95% CI 82.4%, 100.0%), of the patients reported reactions to Amoxicillin, while 5.88% (95% CI 0.00%, 17.6%) reacted to Clavulin and 5.88% (95% CI 0.00%, 17.6%) to Cefprozil. The most common symptoms were arthritis/arthralgia (100.0% [95%CI, 87.4%, 100.0%]), urticaria (64.7% [95%CI, 46.5%, 79.7%]), and macular/papular rash (47.1% [95% CI 30.2%, 64.6%]). History of drug allergy in a parent was found in 23.5% (95% CI 11.4%, 41.7%). Amongst the 34 patients, 2.94% (95% CI 0.00%, 14.3%) reacted immediately to a graded oral challenge (within 1 h) and 2.94% (95% CI 0.00%, 14.3%) had a non-immediate reaction. Of the 29 negative challenge patients eligible for follow-up, 19 (65.5%) patients responded and 8 patients (42.1%) reported subsequent use of the culprit antibiotic, of which 50.0% [95% CI 21.5%, 78.5%)].

**Conclusion:** A graded oral challenge is a safe procedure in patients presenting positive SSLR. However, there is a high rate of repeat reactions with subsequent use of the culprit antibiotic.

### A73 Efficacy of omalizumab in adults with chronic inducible urticaria refractory to histamine receptor antagonists

#### Shun Chi Ryan Lo^1^, Deena Kobric^2^, Gordon Sussman^3^

##### ^1^Department of Medicine, University of British Columbia, Vancouver, British Columbia, Canada; ^2^Gordon Sussman Clinical Allergy Research Inc., Toronto, Ontario, Canada; ^3^Division of Allergy and Clinical Immunology, Department of Medicine, University of Toronto, Toronto, Ontario, Canada

**Correspondence:** Deena Kobric

*Allergy, Asthma and Clinical Immunology* 2019, **15(Suppl 1):**A73

**Background:** Omalizumab is currently approved by the FDA for treatment of chronic spontaneous urticaria (CSU) refractory to histamine receptor antagonists. Although the efficacy of omalizumab in CSU is well published, its use in chronic inducible urticaria (CINDU) is under studied. We aimed to further characterize the effect of omalizumab on refractory CINDU and its subtypes through off-label use and subsequent monitoring of symptoms.

**Methods:** Starting in 2014, patients presenting with CINDU refractory to high-dose histamine receptor antagonists were offered omalizumab. Starting dose was 300 mg intramuscularly monthly, with uptitration to 450 mg and 600 mg if the patient is unsatisfied with any ongoing symptoms. Follow-up was every 3 months. Symptom control was clinically evaluated at each visit. A decrease in urticaria severity was deemed a response, and patients without any further symptoms were deemed to be in remission.

**Results:** Among 21 patients treated with omalizumab, 14 (67%) demonstrated response, and 9 (43%) were in remission. Six patients presented with cholinergic urticaria, 5 with symptomatic dermographism, 3 with delayed-pressure urticaria, 3 with solar urticaria, 2 with cold urticaria, 1 with heat urticaria, and 1 with aquagenic urticaria, with response rates of 83%, 60%, 100%, 67%, 50%, 100%, and 0%, and remission rates of 50%, 40%, 67%, 0%, 50%, 100%, and 0% respectively. Dose was uptitrated to 450 mg in 1 patient with cholinergic urticaria, and 1 patient with solar urticaria; and to 600 mg in 1 patient with symptomatic dermographism.

**Conclusion:** Our data contributes to the growing body of evidence that omalizumab is efficacious in CINDU which currently has limited treatment options. A portion of patients showed a lack of response despite uptitration, which may imply that CINDU has both IgE-mediated and non-IgE mediated mechanisms. Further studies with greater recruitment and a randomized approach is warranted.

### A74 Evaluation of C1 inhibitor utilization in British Columbia

#### Kateryna Vostretsova^1^, Amin Kanani^2^

##### ^1^Allergy and Immunology, University of British Columbia, Vancouver, BC, V5R 3V8, Canada; ^2^Allergy and Immunology, University of British Columbia, Vancouver, BC, V5Z 1M9, Canada

**Correspondence:** Kateryna Vostretsova

*Allergy, Asthma and Clinical Immunology* 2019, **15(Suppl 1):**A74

**Background:** Hereditary angioedema (HAE) is an autosomal dominant condition resulting in recurrent submucosal or subcutaneous angioedema as a result of C1 inhibitor deficiency. Extensive research into the pathophysiology of this condition has provided for better life-saving treatments however data surrounding utilization and cost of treating this condition are scarce.

**Methods:** Data was obtained with permission from the British Columbia Provincial Blood coordinating office, which has access to the central transfusion registry and data from Canadian Blood Services. Out of the 6 health authorities in British Columbia (BC), Fraser Health data was used as it is the region with highest use of C1 Inhibitor and it is one of two authorities with a formal home infusion program. Discharge abstract database was also used to assess the number of times C1 inhibitor was administered from 2007/08 to 2014/15.

**Results:** C1inhibitor utilization has been slowly increasing across the country. In 2016/2017, there was a 29.6% and 9.3% increased use in Canada and BC respectively with Fraser Health accounting for 57% of BC utilization. As of Dec 2016, 26 patients have been trained as part of the home infusion program and 12 patients have used C1Inhibitor at home resulting in more doses of prophylaxis administered in the home setting and less overall emergency department visits after training (53.7 vs. 38 annual number of Emergency Department (ED) visits). Those trained were also more likely to be diagnosed with HAE during admission preventing unnecessary delays in treatment.

**Conclusion:** The demand for C1 Inhibitor is on the rise. The reasons for this are multiple including better awareness and understanding of HAE along with easier access through home infusion programs leading to less ED visits. Future studies assessing utilization are needed to gain a better understanding of the increasing use of this life-saving treatment.

### A75 Prevalence of skin prick test positivity to inhalant allergens in patients with chronic spontaneous urticaria (CSU): a systematic literature review

#### Melanie Wong^1^, Paul K. Keith^2,3^

##### ^1^Faculty of Health Sciences McMaster University, Hamilton, Ontario, Canada; ^2^Department of Medicine, McMaster University, Hamilton, Ontario, Canada; ^3^Division of Clinical Immunology & Allergy, McMaster University, Hamilton, Ontario, Canada

**Correspondence:** Melanie Wong

*Allergy, Asthma and Clinical Immunology* 2019, **15(Suppl 1):**A75

**Background:** Current guidelines do not recommend performing aeroallergen skin prick testing (SPT) in chronic spontaneous urticaria (CSU). The objective of this review was to investigate the prevalence of aeroallergen sensitization and allergic inflammation in patients with CSU.

**Methods:** Systematic literature reviews were conducted using the PubMed, Medline and Google Scholar databases to identify all English studies published in peer-reviewed journals between 1991 and 2018 that evaluated the prevalence of markers of allergic inflammation to aeroallergens and total IgE in CSU patients.

**Results:** Seventeen studies included a total of 2569 CSU patients. The prevalence of aeroallergen-positive SPT in all CSU patients tested was 34%. Five studies included a healthy control group; positive tests to one or more aeroallergens were present in 39.3% of CSU patients compared to 19.1% in controls (*P *= 0.096). SPT sensitivity to house dust mite was positive in 34.2% of CSU patients compared to 11.4% of controls. CSU patients were 4.2 times more likely to be sensitized to house dust mite (95% CI 1.8–10) compared to controls (*P *= 0.02). The prevalence of positive personal history of atopic conditions was described in five studies, with allergic rhinitis (17.1%) and asthma (7.9%) being the most common. The rate of SPT positivity and presence of allergic rhinitis and asthma may have been underreported, since many patients were on high-dose antihistamines and/or systemic steroids. Fourteen studies involving 1481 CSU patients found the mean total IgE levels to be 211.5 IU/ml (normal < 165 IU/mL), with a range of 133–570.6 IU/mL.

**Conclusion:** Evidence of aeroallergen sensitization was present more commonly in CSU patients than controls. The presence of sensitization may be important in the pathogenesis of CSU. Further studies may be warranted to determine if specific allergen avoidance, desensitization or improvement in the mucosal allergic inflammation present in asthma and/or rhinitis has any benefit in the management of CSU in sensitized individuals.

### A76 Diagnosis of ibuprofen allergy through oral challenge

#### Judy Gaffar, Sofianne Gabrielli^1^, Christine McCusker^1^, Christos Tsoukas^2^, Moshe Ben-Shoshan^1^

##### ^1^Division of Allergy Immunology and Dermatology, Montreal Children’s Hospital, Montreal, Quebec, Canada; ^2^Department of Medicine, Division of Allergy and Clinical Immunology, McGill University, Montreal, Quebec, Canada; Division of Experimental Medicine, The Research Institute of the McGill University Health Centre, McGill University, Montreal, Quebec, Canada

**Correspondence:** Judy Gaffar

*Allergy, Asthma and Clinical Immunology* 2019, **15(Suppl 1):**A76

**Background:** Non-steroidal anti-inflammatory drugs (NSAIDs), mainly Ibuprofen, are frequently implicated in hypersensitivity reactions in children. Given that Ibuprofen is often used to manage fever and pain in children and given the absence of standardized skin tests in the diagnosis of NSAID allergy, drug challenges are the only available tool to establish the diagnosis of this drug allergy. We aimed to assess the risk of Ibuprofen allergy in patients through the graded oral challenge.

**Methods:** All children referred to the Montreal Children’s Hospital for suspected NSAIDs allergy were recruited for the LACTAAM study between January 2017 and May 2018. A standardized survey of treatment, symptoms, and associated factors was filled and an oral challenge (10% and 90% of the oral dose) was conducted. The families were contacted annually to inquire about subsequent NSAID use and associated reactions. Descriptive statistics were used to characterize the reactions.

**Results:** Twenty-two patients with a reported allergy to Ibuprofen were recruited. The majority of the reactions (68%) occurred within 1 hour after using the medication and 95% occurred within the first 3 days of taking the drug. The most common symptom was angioedema (59%). All children underwent an oral challenge and five patients (22.73% [95% CI 8.69%, 45.82%]) had a positive challenge, three of which were immediate reactions and two non-immediate reactions. Among the five patients, symptoms were consistent with anaphylaxis in three (60%), and two (40%) were treated with epinephrine. Among the 17 patients with negative oral challenge eligible for follow-up, nine (53%) patients responded. Of the contacted patients, six (67%) reported subsequent Ibuprofen use of which one patient (16.67% [95% CI 0.88%, 63.52%]) reacted and had symptoms consistent with anaphylaxis.

**Conclusion:** Graded oral challenges can be used to diagnose an allergy to Ibuprofen. However, there is a 17% risk of subsequent reaction despite negative challenge in this small cohort.

### A77 Review of cold-induced urticaria characteristics, diagnosis and management in a western Canadian allergy practice

#### Peter Stepaniuk^1^, Kateryna Vostretsova^2^, Amin Kanani^2^

##### ^1^Department of Internal Medicine, University of British Columbia, Vancouver, BC, Canada; ^2^Division of Allergy and Immunology, University of British Columbia, Vancouver, BC, Canada

**Correspondence:** Peter Stepaniuk

*Allergy, Asthma and Clinical Immunology* 2019, **15(Suppl 1):**A77

**Background:** Cold-induced urticaria is a significant condition, especially among young females. Despite the morbidity of this disease, studies that fully characterize the disease are limited.

**Methods:** We analyzed the characteristics of patients diagnosed with cold-induced urticaria at a community-based allergy practice in Vancouver, BC, Canada between 2003 and 2016. Detailed patient history, diagnostic measures and treatment were evaluated.

**Results:** A total of 50 patients were found to have active cold-induced urticaria with a median age of 28.5 (range 2–67) years and 35 patients (70%) were female. 16 patients (32%) had a co-occurring physical urticarias while 26 patients (52%) had secondary allergic diagnoses and 3 patients (6%) were thought to have a provoking factor. Of those with a clinical history of suspected cold-induced urticaria that were evaluated with ice cube testing, a positive test was obtained in 84.7% of patients. Treatment was largely with non-sedating antihistamines, with the majority of patients receiving this modality.

**Conclusions:** Cold-induced urticaria is a complex disease with significant overlap with other chronic inducible urticarias and other allergic diseases. Diagnostic testing shows inconsistent results and the mainstay of treatment consists of non-sedating antihistamines, with other options available for those who do not respond.

### A78 Lanadelumab improves health-related quality of life in patients with hereditary angioedema (HAE): findings from the HELP study

#### Gagan Jain^1^, Gordon Sussman^**2**^, William R. Lumry^3^, Peng Lu^1^, Hannah Lewis^4^, Marcus Maurer^5^

##### ^1^Shire, Lexington, MA, USA; ^2^University of Toronto, Toronto, Canada; ^3^Allergy and Asthma Research Associates Research Center, Dallas, TX, USA; ^4^ICON plc, London, UK; ^5^Department of Dermatology and Allergy, Allergie-Centrum-Charité, Charité–Universitätsmedizin Berlin, Berlin, Germany

**Correspondence:** Gagan Jain

*Allergy, Asthma and Clinical Immunology* 2019, **15(Suppl 1):**A78

**Objective:** HAE negatively impacts daily life. Effect of lanadelumab on health-related quality-of-life (HRQoL) was assessed in the HELP Study.

**Methods:** Patients with HAE were randomized to 26 weeks of treatment with lanadelumab 150 mg-q4wks (n = 28), 300 mg-q4wks (n = 29), 300 mg-q2wks (n = 27), or placebo (n = 41). HRQoL was assessed using the Angiodema Quality of Life (AE-QoL) questionnaire, including total score and 4 domain scores; lower scores reflect less impairment. Changes in AE-QoL total scores were measured; minimal clinically important difference = 6 points. Changes in functioning domain scores, as well as functioning domain item-level responses at Day 0 and Day 182 were assessed descriptively.

**Results:** At Day 0, HRQoL impairment was similar for placebo, lanadelumab 150 mg-q4wks, 300 mg-q4wks, and 300 mg-q2wks arms: mean ± SD AE-QoL total scores were 42.8 ± 17.5, 48.8 ± 20.3, 47.5 ± 21.9, and 43.8 ± 16.8, respectively. At Day 182, lanadelumab arms had clinically meaningful reduction (i.e., improvement) in AE-QoL total score: mean ± SD − 19.8 ± 19.1, − 17.4 ± 18.7, and − 21.3 ± 18.4, respectively, compared with − 4.7 ± 18.8 for placebo. At Day 0, functioning domain impairment was similar for placebo, lanadelumab 150 mg-q4wk, 300 mg-q4wks, and 300 mg-q2wks arms: mean ± SD scores were 43.2 ± 24.8, 47.3 ± 24.4, 44.5 ± 24.4, and 43.1 ± 24.1, respectively. At Day 182, reductions in scores were substantially higher in the lanadelumab arms: mean ± SD − 27.8 ± 23.1, − 24.3 ± 22.7, and − 36.0 ± 22.3 points, respectively, compared with − 5.4 ± 22.7 points with placebo. Whereas at Day 0 a similar percentage of patients in all treatment groups were restricted with regard to work, physical activity, leisure time, and social relations, at Day 182 a higher proportion of patients receiving placebo than those receiving lanadelumab 150 mg-q4wks, 300 mg-q4wks, and 300 mg-q2wks were “occasionally, often, or very often” restricted with regard to these item-level responses, including work (47%, 31%, 14%, 4%); physical activity (55%, 23%, 32%, 12%); leisure time (45%, 15%, 21%, 8%); and social relations (50%, 23%, 18%, 8%), respectively.

**Conclusions:** Patients receiving lanadelumab for HAE prophylaxis showed improvements in HRQoL compared with placebo.

**Funding Source**: Shire HGT, Lexington, MA, USA.

**Acknowledgements:** HELP Study investigators Canada: J. Hébert, B. Ritchie, G. Sussman, W.H. Yang; Germany: C. Escuriola Ettingshausen, M. Magerl, I. Martinez-Saguer, M. Maurer, P. Staubach, S. Zimmer; Italy: M. Cicardi, F. Perego, M.A. Wu, A. Zanichelli; Jordan: A. Al-Ghazawi, M. Shennak; Puerto Rico: R.H. Zaragoza-Urdaz; United Kingdom: H.J. Longhurst, R. Ghurye, E. Zinser; United States: J. Anderson, A. Banerji, A.P. Baptist, J.A. Bernstein, P.B. Boggs, P.J. Busse, S. Christiansen, T. Craig, M. Davis-Lorton, S. Gierer, R.G. Gower, D. Harris, D.I. Hong, J. Jacobs, D.T. Johnston, E.S. Levitch, H.H. Li, R.F. Lockey, P. Lugar, W.R. Lumry, M.E. Manning, D.L. McNeil, I. Melamed, T. Mostofi, T. Nickel, W.R. Otto, A.A. Petrov, K. Poarch, C. Radojicic, S.M. Rehman, M.A. Riedl, L.B. Schwartz, R. Shapiro, E. Sher, A.M. Smith, T.D. Smith, D. Soteres, R. Tachdjian, H.J. Wedner, M.E. Weinstein, H. Zafra, B.L. Zuraw.

## CASE REPORT

### A79 A rare case of anaphylaxis to Indian jujube (Ziziphus Mauritiana)

#### Babak Aberumand^1^, Rozita Borici-Mazi^2^

##### ^1^Department of Medicine, Queen’s University, Kingston, Ontario, Canada; ^2^Division of Allergy & Immunology, Department of Medicine, Queen’s University, Kingston, Ontario, Canada

**Correspondence:** Babak Aberumand

*Allergy, Asthma and Clinical Immunology* 2019, **15(Suppl 1):**A79

**Background:** Indian jujube (Ziziphus mauritiana), also known as Ber, is a tree of the family Rhamnaceae that bears a sweet fruit. It is native to tropical and subtropical regions of Asia that include Southern China, India and Malaysia [1]. A few case reports have described a latex-fruit syndrome in which there is a cross-reaction between the allergenic components of latex and the Indian jujube [2–4]. We present the first case of an anaphylactic reaction to this fruit in a patient with no previous history of allergies.

**Case presentation:** A 55-year-old male was referred to the Outpatient Allergy Clinic at Queen’s University for evaluation of anaphylaxis caused by ingestion of Indian jujube. He presented to the Emergency Department (ED) with scalp pruritus, dyspnea and generalized urticaria, which occurred 2 h after he had consumed a homemade candied fruit cocktail consisting of Indian jujube, water, and a Tai and Indian sweetener. In the ED, he was treated with epinephrine, intravenous diphenhydramine and steroids. He did not endorse any previous history of environmental or food allergies, but had consumed this fruit frequently since childhood. In clinic, he underwent skin-prick testing with a diluted saline slurry (1/20 w/v) of candied jujube, which resulted in a positive wheal and flare response with appropriate controls. On subsequent visit, skin-prick tests were performed with full strength (1/10 w/v) saline slurries of the Tai and Indian sweetener used to make the cocktail. Both tests were negative. He was diagnosed with an IgE-mediated anaphylactic reaction to the jujube fruit. He was advised to avoid consumption of jujubes and carry an epinephrine autoinjector.

**Conclusions:** Anaphylaxis secondary to Indian jujube ingestion is an extremely rare phenomenon in patients with or without a latex allergy. Further studies are needed to help elucidate the underlying mechanism of this fruit’s ability to trigger a life-threatening condition.

**Statement of consent:** Consent to publish was obtained from the patient [or guardians of the patient] involved in this study.

### A80 Successful implantation after treatment with subcutaneous immunoglobulin in a patient with APECED and primary infertility due to premature ovarian failure

#### Salma Alkhammash, Geneviève Genest

##### Division of Clinical Immunology & Allergy, Department of Medicine, McGill University, Montreal, Quebec, Canada

**Correspondence:** Salma Alkhammash

*Allergy, Asthma and Clinical Immunology* 2019, **15(Suppl 1):**A80

**Introduction:** Autoimmune polyendocrinopathy-candidiasis-ectodermal dystrophy (APECED) (MIM 240300), is a rare autosomal recessive disease caused by underlying mutation of the Autoimmune Regulator (AIRE) gene, which promotes deletion of self-reactive T lymphocytes and prevents autoimmunity. Female patients with APECED have a 60% risk of developing autoimmune primary ovarian failure (a-POF) before they reach the age of 30. In-vitro-fertilization with donor egg may enable conception but literature on the subject is scant and outcomes of fertility treatments in such patients is unknown. Presumably, implantation and pregnancy outcomes should be low due to the presence of auto-antibodies to cytokines that regulate the immune events crucial to early pregnancy. We report the case of a patient with APECED POF who successfully underwent embryo transfer after pre-conceptual treatment with subcutaneous immunoglobulin (scIg).

**Case presentation:** A 29-year old woman with APECED manifesting as with hypothyroidism, hypoparathyroidism, adrenal insufficiency, nephrocalcinosis, arthritis and POF consulted our clinic for recurrent implantation failure. Her egg donor was 24 and 25 at the time of oocyte retrievals and fertilization yielded excellent quality blastocysts. 8 transfers were sequentially performed with ideal endometrial preparation; 6 failed, one yielded a biochemical pregnancy and the other, a 5 4/7 week miscarriage. Recurrent implantation failure workup was negative and apart from organ-specific auto-immunity, no evidence of systemic inflammation/autoimmunity was found. ScIg was initiated on a weekly basis at 600 mg/kg one month prior to embryo transfer. She is currently at 17 weeks gestation with a viable intra-uterine pregnancy.

**Conclusions:** Immunoglobulins (IG) have been used for years as an off-label treatment to improve fertility and pregnancy outcomes in women with suspected immune-mediated reproductive failure. Its use in patients with APECED POF has not been reported to our knowledge. As APECED may cause auto and alloimmune implantation failure or miscarriage by interfering with cytokine signals required to direct decidualization and placenta formation in early pregnancy, neutralization of such anti-cytokine antibodies by IG may provide a mechanism by which IG improves pregnancy outcomes in such patients. Should this pregnancy succeed, further studies will be needed to evaluate the effectiveness of IG in patients with APECED POF and to elucidate underlying mechanisms of endometrial immune regulation.

**Consent to publish:** was obtained from the patient involved in this study.

### A81 Hypersensitivity pneumonitis in the setting of electronic cigarette use: a case report

#### Karver Zaborniak, Chrystyna Kalicinsky

##### Department of Allergy and Clinical Immunology, University of Manitoba, Winnipeg, Manitoba, Canada

**Correspondence:** Karver Zaborniak

*Allergy, Asthma and Clinical Immunology* 2019, **15(Suppl 1):**A81

**Background:** Hypersensitivity pneumonitis is a complex syndrome caused by immunological reaction to inhaled organic and/or inorganic materials [1]. The epidemiology of this diagnosis is largely unknown; likely owing to the absence of internationally recognized diagnostic criteria. Hypersensitivity pneumonitis is classified as acute or chronic on the basis of several factors, such as the length of exposure and duration of symptoms [2]. Treatment of hypersensitivity pneumonitis is based on antigen avoidance and immune suppression.

**Case presentation:** A 73-year-old female was admitted to hospital with a history of dyspnea and non-productive cough. Past medical history was significant for type-2 diabetes mellitus, osteoarthritis, and a 50-pack year smoking history. Social history revealed electronic cigarette use for smoking cessation. Physical examination was significant for a room air saturation of 82%, and bilateral inspiratory crackles. A CT chest revealed diffuse ground glass opacities in the upper lung fields, associated subplueral septal thickening, traction bronchiectasis, and areas of honeycombing. Bronchial washings did not yield positive culture results for bacterial, fungal, or mycobacterial agents. Screening autoimmune testing was negative, and a nasal swab sample was negative for viral agents. A diagnosis of chronic hypersensitivity pneumonitis relating to electronic cigarette use was made, and the patient was started on a tapering dose of prednisone. At a 1 month follow-up, the patient was noted to be symptom free, and chest radiography revealed significant interval improvements.

**Conclusions:** In the absence of recognized diagnostic criteria, hypersensitivity pneumonitis requires a high-index of suspicion for diagnosis. This case is among the first to demonstrate a link between electronic cigarettes and the development of hypersensitivity pneumonitis.

**Statement of consent:** Consent to publish was obtained from the patient involved in this study.

### A82 Case report: can an insulin allergy be desensitized?

#### Jodi Valois^1^, Stephanie Santucci^1^, Cathy Sun^2^, Heidi Dutton^2^, William H. Yang^1,2^

##### ^1^Ottawa Allergy Research, Ottawa, Ontario, Canada; ^2^University of Ottawa Medical School, Ottawa, Ontario, Canada

**Correspondence:** Jodi Valois

*Allergy, Asthma and Clinical Immunology* 2019, **15(Suppl 1):**A82

**Background:** Allergies to Insulin are rare; the prevalence of having an allergy to insulin therapy is 0.1–3%. Immediate hypersensitivity reactions (type 1) are the most common and in rare cases can cause anaphylaxis. Insulin desensitization has been proven as an effective treatment for developing a tolerance to insulin.

**Case presentation:** R.S is a 68-year-old Caucasian male. He was diagnosed with type 2 Diabetes Mellitus 25 years ago and has a history of pruritus and urticaria after subcutaneous administration of various brands of premixed insulin. Skin prick testing revealed a positive reaction to Glulisine, Glargine, Detemir, Lispro, Novorapid, Novomix, and Aspart. His HbA1C at the time of skin testing was 10.2%. Based on the patient’s history and recent hospitalizations he was determined to be an ideal candidate for rapid insulin desensitization. The insulin desensitization was performed in a controlled environment with a goal of reaching 50units of Glargine insulin. Overall the patient tolerated the desensitization very well with minor site reactions and pruritus. Presently the patient continues to tolerate 50units of Glargine qhs with Cetirizine for pruritus and has a HbA1C of 8%.

**Conclusions:** Rapid subcutaneous Insulin desensitization can be successful in treating patients with an Insulin allergy. Careful selection of patients for this procedure is necessary.

**Statement of consent:** Consent to publish was obtained from the patient involved in this study.

### A83 Malignancy in the setting of hereditary angioedema (HAE): a possible consequence of chronically low C4 from C1-INH deficiency or dysfunction

#### Paul Oykhman, Paul K. Keith

##### Department of Medicine, Division of Clinical Immunology and Allergy, McMaster University, Hamilton, Ontario, Canada

**Correspondence:** Paul Oykhman, Paul K. Keith

*Allergy, Asthma and Clinical Immunology* 2019, **15(Suppl 1):**A83

**Background:** HAE is a condition characterized by recurrent angioedema in the setting of deficiency of C1-inhibitor (C1-INH), which in turn leads to hyper-activation of kallikrein and overproduction of bradykinin. The lack of potent inhibition of C1r and C1 s by C1-INH results in chronically low/undetectable levels of C4. Previous reports have demonstrated increased risk of autoimmune disease and lymphoproliferative disorders in patients with congenital C4 deficiency [1]. This raises the possibility that low C4 in HAE may also predispose to these conditions. This may in turn influence the choice of therapy and treatment targets. We describe a case of a patient with HAE with chronically low C4 who went on to develop splenic B cell lymphoma.

**Case presentation:** A 52 yo F with recurrent episodes of acute abdominal pain since the age of 20 (with prior surgery for suspected diverticulitis) as well as a family history of angioedema in her mother was diagnosed with HAE after she presented with laryngeal edema requiring a tracheostomy. She was found to have an undetectable C4 of < 0.04 g/L (0.13–0.52 g/L) and a low C1 inhibitor level at < 0.03 (0.12–0.35 g/L*).* She went on to develop recurrent attacks of laryngeal and suspected bowel wall edema, initially treated with danazol, followed by IV C1-INH on demand treatment. Although her attacks reduced in frequency, her C4 levels remained chronically low/undetectable. At 70 years old, she was diagnosed with splenic B cell lymphoma with marrow involvement. Soon after she was admitted with sepsis and unfortunately died despite treatment.

**Conclusions:** As congenital C4 deficiency has been associated with an increased risk of malignancy, future consideration may need to be given to whether HAE with low C4 is best managed by C1-INH replacement at doses which restore C4 levels versus treatments, which only prevent kallikrein activation but do not restore C4 levels.

**Statement of consent:** Consent to publish was obtained from the patient involved in this study.

### A84 A case of selective IgA deficiency: orchestrated by the microbiome?

#### Aaron P. van der Leek^1^, Anne Hicks^1^, Yarden Yanishevsky^1^, Anita L. Kozyrskyj^1,2,3^

##### ^1^Department of Paediatrics, Faculty of Medicine and Dentistry, University of Alberta, Edmonton, Alberta, Canada; ^2^Department of Obstetrics and Gynecology, Faculty of Medicine and Dentistry, University of Alberta, Edmonton, Alberta, Canada; ^3^School of Public Health, University of Alberta, Edmonton, Alberta, Canada

**Correspondence:** Aaron P. van der Leek

*Allergy, Asthma and Clinical Immunology* 2019, **15(Suppl 1):**A84

**Background:** Initially provided by breastmilk, secretory immunoglobulin A (sIgA) interacts with gut microbiota during infancy to shape gut mucosal immunity development. Individuals with selective IgA deficiency (SIgAD) have increased risk for sinopulmonary and gastrointestinal infections, atopy, asthma and autoimmune diseases [1–4]. Gut microbial dysbiosis from antibiotic treatment and/or limited breastfeeding may increase risk of these outcomes in IgA-deficient patients. Currently, SIgAD diagnostics and treatment are limited. This case discussion will focus on an IgA-deficient 4-year-old male seen at 18 months for failure to thrive and chronic sinopulmonary infection [3,5,6].

**Case presentation:** The patient presented with recurrent pneumonia, failure to thrive and intermittent respiratory distress. He was born early term with low birth weight and had trouble breastfeeding, prompting maternal treatment with the galactagogue, domperidone. Despite a suggestive history of dairy allergy, he now tolerates dairy. Workup demonstrated undetectable IgA and IgG at 6 months, with undetectable IgA and normal IgG at 18 months. Other immune system markers were normal and cystic fibrosis was ruled out. The patient had symptoms suggestive for asthma and responded well to inhaled corticosteroid and bronchodilator; though by 3 years asthma symptoms had resolved. The patient also had episodes consistent with pneumonia, which lessened in frequency and severity over time. By age 1, he had received 2 courses of clarithromycin and one of amoxicillin, all in succession.

**Conclusions:** Diagnosis of IgA deficiency is controversial. Prompt recognition and treatment of suspected bacterial infections is the mainstay of therapy. Identifying pre-postnatal exposures that impact the gut microbiome and sIgA levels may elucidate preventable contributors to the rising incidence of atopic, inflammatory and infectious disease associated with this diagnosis. This case highlights the relationship between breastfeeding, birth weight, antibiotic therapy and the development of atopy in IgA deficiency [4]. Consent to publish was obtained from the patient [or guardians of the patient] involved in this study.

**Consent to publish:** was received from the patient involved in this study.

### A85 Hypereosinophilic syndrome: a diagnostic dilemma

#### Rongbo Zhu^1^, Reena Pattani^1,2^

##### ^1^Department of Medicine, University of Toronto, 190 Elizabeth Street, R. Fraser Elliott Building 3-805, Toronto, ON, M5G 2C4, Canada; ^2^Division of General Internal Medicine, St. Michael’s Hospital, 30 Bond Street, Toronto, ON, M6H2X5, Canada

**Correspondence:** Rongbo Zhu

*Allergy, Asthma and Clinical Immunology* 2019, **15(Suppl 1):**A85

**Background:** Hypereosinophilic syndrome (HES) is a heterogeneous disorder characterized by hypereosinophilia (> 1.5 × 10^9^/L) with eosinophil-mediated end organ damage. We present a case that showcases the diagnostic challenges of HES.

**Case presentation:** A 31-year-old man presented with a 2 week history of worsening SOB, cough, diarrhea, and constitutional symptoms. He had a history of asthma with 2 years of chronic cough and recently travelled to Pakistan for 2 months. Physical exam showed diffuse wheeze. His bloodwork was significant for eosinophilia at 2.1 (0.04–0.40 × 10^9^/L) and a rise in his troponin at 0.101 ug/L (< 0.040) without ECG changes. He was admitted for a workup of HES. Subsequent workup showed normal electrolytes/creatinine/urinalysis/liver enzymes/function. IgE was elevated at 1911 kIU/L (1.0–165.0). SPEP was non-diagnostic. Infectious workup was negative for ova/parasites (strongyloides/toxicara/shistosomiasis/trinchinella/HIV/HepB/HepC). ANA/ANCAs were negative and complements/tryptase/vitamin B12/AM cortisol were normal. CT Chest/Abdomen/Pelvis demonstrated extensive mediastinal lymphadenopathy. Cardiac MRI was suggestive of eosinophilic myocarditis. He underwent a transbronchial biopsy showing eosinophilic infiltrates but negative for AFB/fungus/lymphoma. His colonoscopy/EGD was negative for infection/malignancy. A bone marrow biopsy showed cellular differential of 54% eosinophils but no neoplastic process. Flow cytometry was non-diagnostic. Genetic testing for PDGFR/FIP1L1 was negative. He developed non-painful numbness of his calf/soles in hospital. A sural nerve biopsy showed Wallerian degeneration which was non-diagnostic. He was provisionally diagnosed with ANCA-negative eosinophilic granulomatosis with polyangiitis (eGPA) vs idiopathic HES and was started on high dose steroids. His condition improved significantly and he was discharged with close follow-up.

**Conclusions:** HES can be diagnostically challenging. A systematic approach is important to ensure an infection/malignancy is ruled out before subjecting patients to high dose corticosteroids. In this case, despite the negative ANCA and absence of diagnostic pathology, the diagnosis of eGPA was favoured due to the asthma-like symptoms at presentation.

**Statement of consent:** Consent to publish was obtained from the patient involved in this case.

### A86 Acquired angioedema secondary to multiple myeloma: a mimicker of intraperitoneal carcinomatosis resulting in surgery

#### Sanju Mishra, Harold Kim

##### Department of Medicine, Division of Clinical Immunology & Allergy, Western University, London, ON, Canada

**Correspondence:** Sanju Mishra

*Allergy, Asthma and Clinical Immunology* 2019, **15(Suppl 1):**A86

**Background:** Acquired angioedema due to acquired C1-inhibitor deficiency (C1-INH-AAE) is a rare condition that presents with recurrent episodes of swelling. C1-INH-AAE is most commonly associated with lymphoproliferative disorders. Presentation is variable with swelling of the tongue and lips being most prominent; however, less common presentations, such as angioedema of the gut, can produce symptoms that may mimic peritonitis. On abdominal imaging, ascites is often identified, which may cause clinicians to overlook rare causes such as C1-INH-AAE [1].

**Case presentation:** BM is a 70-year-old female who was diagnosed with monoclonal gammopathy of undetermined significance (MGUS) in 2004. Her disease progressed to smouldering myeloma in May 2016. Soon afterwards, BM began experiencing symptoms, which in retrospect were consistent with C1-INH-AAE. She had an episode of swelling of the lips and tongue requiring an emergency department visit. She also began experiencing recurrent episodes of abdominal pain which was associated with diarrhea. On an abdominal CT completed in March 2017, ascites and possible peritoneal metastases were visualized. A paracentesis was attempted the next week, but no ascites was present. Based on her presentation she was assessed by the Gynecology service and a laparotomy, abdominal hysterectomy, bilateral salpingo-oophorectomy and infracolic omentectomy were completed. All pathology was negative for malignancy. Based on the episode of angioedema, a referral was sent to Allergy/Immunology. There were concerns of C1-INH-AAE and bloodwork was consistent with this diagnosis. She was referred back to Hematology where progression to multiple myeloma was confirmed. With treatment of multiple myeloma, including: chemotherapy and stem cell transplant, the episodes of abdominal pain and diarrhea have ceased.

**Conclusions:** The case illustrates the challenge of diagnosing C1-INH-AAE and emphasizes the importance of considering this diagnosis in patients with potential angioedema symptoms and hematologic malignancy.

**Statement of consent:** Consent to publish was obtained from the patient.

### A87 Refractory urticaria and the importance of diagnosing Schnitzler’s syndrome: a case report and review of the literature

#### Carol Saleh^1^, Tina Nham^2^, Derek K. Chu^3^, Deborah M. Siegal^4^

##### ^1^Department of Medicine, McMaster University, Hamilton, Ontario, Canada; ^2^Michael G. DeGroote School of Medicine, McMaster University, Hamilton, Ontario, Canada; ^3^Division of Clinical Immunology and Allergy, Department of Medicine, McMaster University, Hamilton, Ontario, Canada; ^4^Division of Hematology and Thromboembolism, St. Joseph’s Healthcare, Hamilton, Ontario, Canada

**Correspondence:** Deborah M. Siegal

*Allergy, Asthma and Clinical Immunology* 2019, **15(Suppl 1):**A87

**Background:** Chronic urticaria (CU) affects 1% of the U.S. population and is responsive to antihistamines and/or omalizumab [1]. Schnitzler’s syndrome is a rare autoinflammatory disorder that presents similarly to CU, but is distinguished by fevers, bone pain and immunoglobulin (Ig) M monoclonal gammopathy. Moreover, Schnitzler’s syndrome is refractory to standard CU treatment and patients develop progressive, disabling symptoms.

**Case presentation:** We report a case of Schnitzler’s syndrome in a 52-year old male who presented with CU. He developed fatigue, fever, weight loss, arthralgia and bone pain. Over time, laboratory investigations became significant for microcytic anemia, neutrophilia and elevated c-reactive protein, erythrocyte sedimentation rate and IgE levels, in addition to an IgM monoclonal protein. He achieved partial remission with omalizumab, cyclosporine and cetirizine. After 6 years, his worsening symptoms and abnormal investigations lead to a diagnosis of Schnitzler’s syndrome. Initiation of treatment with the interleukin (IL)-1 receptor antagonist, anakinra, led to a rapid and complete resolution of his symptoms.

**Conclusions:** Schnitzler’s syndrome is a disabling condition that is difficult to diagnose due to its rarity and similarity to benign conditions like CU. We highlight the importance of considering Schnitzler’s syndrome in the setting of refractory CU, particularly when accompanied by systemic symptoms and abnormal investigations. Anakinra is a highly effective treatment suggesting IL-1 has a pivotal role in its pathogenesis, however this is the first case to document elevated IgE levels in Schnitzler’s syndrome, which may have contributed to the partial remission with omalizumab and delay in diagnosis.

**Statement of consent:** Consent to publish was obtained from the patient involved in this study.

### A88 A case of shrimp allergy transfer through stem cell transplantation

#### Andrew Wong-Pack^1^, Pascale Dupuis^2^, Matthew D’Arsie^1^, Harold W. Kim^2,3^

##### ^1^Schulich School of Medicine and Dentistry, Western University, London, Ontario, Canada; ^2^Division of Clinical Immunology and Allergy, Western University, London, Ontario, Canada; ^3^Division of Clinical Immunology and Allergy, McMaster University, Hamilton, Ontario, Canada

**Correspondence:** Andrew Wong-Pack

*Allergy, Asthma and Clinical Immunology* 2019, **15(Suppl 1):**A88

**Background:** Food allergy transfer following solid-organ transplantation and less commonly, stem cell transplantation (SCT) is a complication that is increasingly being recognized [1, 2]. Proposed mechanisms of action for the development of food allergy include the passive transfer of preformed donor IgE and of allergen-specific T and B cells [3]. We describe a case of shrimp allergy transfer after a successful allogeneic SCT for treatment of aplastic anemia.

**Case presentation:** A 49-year-old female received an allogeneic stem cell transplant from her brother in 1996 for treatment of aplastic anemia. Prior to transplantation she consumed shellfish regularly. Upon first re-exposure to shrimp post-transplant, she immediately developed facial angioedema, throat pruritus and shortness of breath. She self-treated with diphenhydramine and the angioedema resolved over days. She was seen by an allergist and diagnosed with a crustacean allergy. She had one other episode of anaphylaxis in 2006 after probable ingestion of a crustacean at a party. Recently, her skin prick test to shrimp was 5 mm, crab 3 mm and lobster 3 mm. The patient’s donor has a long-standing history of crustacean allergy. With crab and shrimp, he experienced tongue numbness, throat discomfort, and shortness of breath that was associated with asthma at the time. His skin prick testing was positive to shrimp 5 mm, crab 3 mm, and lobster 3 mm while on cetirizine 20 mg daily.

**Conclusions:** Food allergy transfer following SCT has seldom been described in the literature. To our knowledge, this is the first case report of shrimp allergy transfer. Although a rare occurrence, clinicians should be aware of the donor’s food allergies, and counsel patients appropriately regarding the potential and implication of food allergy transfer.

**Statement of consent:** Consent to publish was obtained from both the patient and sibling involved.

### A89 An unusual case of spontaneous remission of cold urticaria

#### Deena Kobric^1^, Caitlin Carrigan^2^, Safi Zaidi^1^, Gordon Sussman^1,3^

##### ^1^Gordon Sussman Clinical Research Inc., Toronto, Ontario, Canada; ^2^McMaster University, Hamilton, Ontario, Canada; ^3^University of Toronto, Toronto, Ontario, Canada

**Correspondence:** Deena Kobric

*Allergy, Asthma and Clinical Immunology* 2019, **15(Suppl 1):**A89

**Background:** Cold urticaria is the fourth most common type of physical urticaria with an incidence rate of 0.05% and average disease duration of 7.9 ± 5.8 years [1]. Symptoms include urticaria, angioedema, and not as commonly, hypotension when exposed to cold temperatures [2]. It is diagnosed by an ice cube test, however, it can also be diagnosed through patient history or a TempTest. The TempTest was developed as an accurate tool for the diagnosis of temperature sensitive urticaria. The device produces temperatures between 4 and 44 °C allowing for threshold temperatures to be determined.

**Case presentation:** An 18-year-old male presented with urticaria following swimming starting 2 weeks prior to his allergic evaluation. During initial evaluation a TempTest was performed, determining that his threshold was 22 °C, there was no family history of cold contact urticaria, and no history of fever, bone pain, or fatigue, the physical examination was normal. The patient was prescribed high dose second generation H1-antihistamines. At his next appointment, approximately 6 weeks following onset of symptoms, his threshold was 16 °C while taking the prescribed medication. He continued the regimen for 3 month at which time he was no longer experiencing symptoms. Five months post-onset of the patient’s first symptoms, a TempTest was performed while the patient wasn’t taking antihistamines and was found to be negative. The patient has been asymptomatic and in remission for 6 months.

**Conclusions:** Previous studies have considered the spontaneous remission rate of cold urticaria over longer periods of time; however, the remission rate within the first year of onset of symptoms has not been widely studied. Thus, research should be done to determine the proportion of patients with cold urticaria who have spontaneous remission within the first year of onset, and examine the mechanism and reason behind the rapid remission.

**Statement of consent:** Written informed consent for publication of their clinical details and/or clinical images was obtained from the patient/parent/guardian/relative of the patient. A copy of the consent form is available for review by the Editor of this journal.

### A90 Anaphylactic reaction to proton pump inhibitors

#### Safi Zaidi^1^, Kate Kim^2^, Deena Kobric^1^, Gordon Sussman^1,3^

##### ^1^Gordon Sussman Clinical Research Inc., Toronto, Ontario, Canada; ^2^National University of Ireland, Galway Medical School, Galway, Ireland; ^3^University of Toronto, Toronto, Ontario, Canada

**Correspondence:** Safi Zaidi

*Allergy, Asthma and Clinical Immunology* 2019, **15(Suppl 1):**A90

**Background:** Proton pump inhibitors (PPIs) are commonly used to treat the gastroesophageal reflux disease, peptic ulcer disease, as well as infections. They act to accumulate weak bases in the acid environment of the canaliculi of the stimulated parietal cell where they are activated, which irreversibly inhibit the H+/K+ ATPase (the proton pump). Histamine-2 blockers (H2 blockers) have also been used for the treatment of acid related disease by being competitive antagonists of the receptor, which inhibit basal acid. Due to the inhibition of acid, it decreases acid secretion in response to histamine, gastrin, and acetylcholine. Both PPIs and H2 blockers are generally well tolerated and anaphylactic reactions are rare to find. There have been several cases of adverse events to PPIs but anaphylaxis to PPIs has rarely been reported.

**Case presentation:** A 65-year-old male patient presents with a history of a mild allergic reaction to one-time use of dexlansoprazole 60 mg 2 years ago featuring an erythematous rash over the face. Recently he was prescribed lansoprazole 30 mg for his gastroesophageal reflux disease (GERD). After taking it for the first time, he developed onset of anaphylaxis with symptoms of urticaria, itchy palms, angioedema, tightness of throat, and shortness of breath. He received 50 mg of diphenhydramine at the pharmacy. 911 was called and the subject was placed on oxygen, given epinephrine 0.5 mg, solumedrol 125 mg, and an additional 50 mg of diphenhydramine.

**Conclusion:** In clinic, famotidine 10 mg was given to the patient as a drug challenge to see if the patient shows symptoms of allergic reaction to H2 blockers. Drug challenge result was negative, as there were no signs of adverse events after observing for over 3 h. From this challenge, the patient is only showing allergic reaction to lansoprazole. Therefore, the patient is allergic to proton pump inhibitors. The patient can continue treating GERD by taking famotidine.

**Statement of consent:** Written informed consent for publication of their clinical details and/or clinical images was obtained from the patient/parent/guardian/relative of the patient. A copy of the consent form is available for review by the Editor of this journal.

### A91 Rare case of estrogen induced dermatitis

#### Hoang Pham, Hanan Ahmed, Amin Kanani

##### ^1^Department of Medicine, Division of Allergy and Clinical Immunology, University of British Columbia, Vancouver, British Columbia, Canada

**Correspondence:** Hoang Pham

*Allergy, Asthma and Clinical Immunology* 2019, **15(Suppl 1):**A91

**Background:** Estrogen induced dermatitis is a rare and challenging entity to diagnose; however, it should be considered in women who present with cyclic rashes that wax and wane with the menstrual cycle^1^. The pathogenesis is not fully understood but the literature suggests a sensitization reaction to relatively elevated levels of endogenous estrogen in the premenstrual phase of the menstrual cycle as well as possible diminished barrier function^2^. Exogenous estrogen exposure may induce autoantibody formation^2, 3^. Skin eruptions are highly variable^1^. Testing has not been standardized but intradermal injection of 0.1 mL of 1 mg/mL estrone is advocated as ideal; others test by oral challenge to ethinyl estradiol^1^. Treatment options reported include tamoxifen, LHRH agonists, and oophorectomy^1^.

**Case presentation:** A 38-year old female with a past medical history of migraines presents with a 3 year history of a cyclical pruritic erythematous maculopapular and plaque rashes—initially distributed to her periorbital and nasolabial areas before generalizing—starting on day 0 or 1 of her menstrual cycle, lasting 3 days before completely resolving. The rash was absent during her entire pregnancy in 2015 but she flared into an intense episode a few days after her C-section. OCPs were used in the past but they never caused this rash and they were discontinued because the patient experienced hair loss. Intradermal testing to medroxyprogesterone was negative. Skin prick testing with oral estrogen was negative. Diagnosis of estrogen induced dermatitis was based on clinical presentation. She currently prefers to manage with bilastine 40–80 mg orally at the time of menses which has decreased her symptoms by 50%

**Conclusions:** Estrogen induced dermatitis is a rare disease that allergists do not routinely diagnose. This diagnosis may be missed if recurrent skin eruptions are not carefully charted out with respect to the menstrual cycle, pregnancy, or variations in estrogen levels.

**Statement of consent:** Written informed consent for the publication of these details was obtained from the patient.

### A92 Anaphylaxis to patent blue dye in a 17-year-old boy

#### Mélanie Leung^1^, Christine T. McCusker^2^, and Moshe-Ben-Shoshan^3^

##### ^1^McGill University, Montreal, QC, Canada; ^2^Division of Allergy and Immunology, Department of Paediatrics, McGill University Health Centre, Montreal, Canada. Meakins-Christie Laboratories of the Research Institute of the McGill University Health Centre, Montreal, Canada; ^3^Division of Pediatric Allergy and Clinical Immunology, Department of Pediatrics, McGill University Health Centre, Montreal, Quebec, Canada

**Correspondence:** Mélanie Leung

*Allergy, Asthma and Clinical Immunology* 2019, **15(Suppl 1):**A92

**Background:** Hypersensitivities to patent blue occur in about 0.1 to 2.8% of patients undergoing sentinel lymph node viewing [1]. Biphasic reactions arise in 23% of drug-induced allergies [2] and were never described in children for patent blue allergy. Documentation on its diagnosis and management is limited. The National Institute of Allergy and Infectious Disease recommends prompt administration of epinephrine, in the risk of anaphylaxis [3]. This case reports a biphasic allergic reaction with anaphylaxis to patent blue in a 17-year-old boy. It highlights the challenges in this diagnosis and management.

**Case description:** A 17-year-old boy underwent a laparoscopic varicocelectomy, at the Montreal Children Hospital. After the injection of patent blue and Ketorolac, he developed rapidly-progressing diffuse hives. Antihistamines, H2 antagonists, and glucocorticoids were administered. 2 h later, he had urticaria, and abdominal pain, and dyspnea. Epinephrine and oxygen were given. Given the temporality of the administration of patent blue and Ketorolac, skin prick tests were performed and were negative. Given the prior tolerance of NSAIDs, only an intradermal test to patent blue was carried and was positive. Avoidance of patent blue, a note in his medical file, and a MedicAlert bracelet were recommended. There was no subsequent allergic reaction.

**Conclusion:** For the patient’s initial reaction, due to the sole presence of cutaneous symptoms, no epinephrine was given. His second reaction could be due to the delayed release of inflammatory mediators or of dye, or the incomplete resolution of his first reaction. We recommend immediate administration of epinephrine to prevent biphasic reactions and in the risk of anaphylaxis. Patent blue allergy should be considered post-surgically and can present without cutaneous blue colorations. This diagnosis is difficult, due to the variety of presentations and its previous absent report in children. To facilitate and to confirm its diagnosis, our case supports the use of skin tests.

**Statement of consent:** *“Consent to publish was obtained from the guardians of the patient involved in this study.”*

### A93 A case of ranitidine-induced exanthema

#### Geneviève Bouvette^1^, Ana-Maria Copaescu^2^, Marie-Soleil Masse^2^

##### ^1^Division of Internal Medicine, Department of Medicine, Centre Hospitalier de l’Université de Montréal (CHUM), Montreal, Quebec, Canada; ^2^Division of Allergy and Clinical Immunology, Department of Medicine, Centre Hospitalier de l’Université de Montréal (CHUM), Montreal, Quebec, Canada

**Correspondence:** Geneviève Bouvette

*Allergy, Asthma and Clinical Immunology* 2019, **15(Suppl 1):**A93

**Background:** Ranitidine is a H_2_-receptor antagonist of the gastric parietal cells commonly used to reduce gastric acid production in gastroesophageal reflux disease (GERD), gastro-duodenal ulcers and hypersecretory states. Available in multiple pharmaceutical forms, this low-cost drug has a well-recognized safety profile [1] making it a drug of choice in multiple care settings. Only a few cases of allergic reactions to ranitidine are described to this day.

**Case presentation:** We report the case of a 45-year-old female who developed three skin eruptions following repetitive exposure to ranitidine. Intradermoreaction (IDR) tests, challenge tests and patch tests were carried out with the medication the patient was exposed to before her eruption in order to identify the responsible drug. Only the patch tests for ranitidine came back positive. Evaluation for cross-reaction with famotidine, another H2-receptor antagonist, came back negative.

**Conclusions:** We report a case of type IV delayed hypersensitivity reaction to ranitidine which presented as a morbilliform skin eruption following exposure to the drug. Despite its widespread use and established safety profile, clinicians should be aware that ranitidine has allergic potential.

**Statement of consent:** Consent to publish was obtained from the patient involved in this study.

### A94 A case of familial cold autoinflammatory syndrome type 2 with NLRP12 mutation

#### Abeer Feteih, Reza Alizadehfar

##### Division of Clinical Immunology & Allergy, Department of Medicine, McGill University, Montreal, Quebec, Canada

**Correspondence:** Abeer Feteih

*Allergy, Asthma and Clinical Immunology* 2019, **15(Suppl 1):**A94

**Background:** Familial Cold Autoinflammatory Syndrome type 2 (FCAS-2) is a rare autosomal dominant disorder associated with a mutation in nucleotide-binding oligomerization domain-like receptor protein 12 (NLRP12). It’s characterized by episodic urticaria, arthralgia, myalgia, and headache. In most patients, the episodes are associated with fever. Majority of patients, not all, have their symptoms triggered by cold exposure. Other symptoms include abdominal and thoracic pain, and sensorineural hearing loss.

**Case presentation:** We present a case of a 46-year-old female who was referred to the primary immunodeficiency clinic 4 years ago for the evaluation of possible Familial Mediterranean Fever (FMF) due to recurrent abdominal pain and family history of FMF. She has a history of ischemic colitis followed by gastroenterology. Her pain didn’t improve with aspirin, asacol, and colchicine. Other symptoms included polyarthralgias, headaches, occasional pruritic rash, nausea, photophobia, frequent liquid bowel movements, and without history of fever. Molecular analysis showed that the patient is a carrier of the pyrine gene mutation but doesn’t have FMF disease because of absence of typical symptoms and no clear response to colchicine. Two years later, the patient’s symptoms didn’t improve despite trial of methotrexate and prednisone. There was no evidence of vasculitis after evaluation by rheumatology. Abdominal arteriogram was normal. The patient had elevated levels of amyloid A level of 29,571, and genetic testing showed autosomal dominant heterozygous mutation in NLRP12 gene with c.838C>T (p.Gln280*) variant. The patient was started on Canakinumab 150 mg subcutaneously every 8 weeks then increased to 300 mg for better symptom control.

**Conclusion:** This case report illustrates a patient with non-typical clinical presentation of FCAS-2 with NLRP12 mutation with a rare variant, which makes this condition challenging to diagnose.

**Statement of consent:** Written informed consent to publish was obtained from the patient.

### A95 Omalizumab as single-dose therapy for vernal keratoconjunctivitis

#### Rachel Simpson^1,2^, Jason K Lee^2,3^

##### ^1^Division of Life Sciences, Department of Biomedical and Molecular Sciences, Queen’s University, Kingston, ON, Canada; ^2^Evidence Based Medical Educator Inc., 123 Edward St., Suite 920, Toronto, ON, Canada; ^3^Section Head of Asthma, Canadian Society of Allergy and Clinical Immunology, Toronto, ON, Canada

**Correspondence:** Rachel Simpson

*Allergy, Asthma and Clinical Immunology* 2019, **15(Suppl 1):**A95

**Background:** Vernal keratoconjunctivitis (VKC) is a bilateral inflammatory ocular condition often brought on by an allergic response. In recent years, omalizumab, an anti-IgE monoclonal antibody, has seen to have promising effects on VKC in children and adults. However, a dosing schedule of omalizumab in VKC cases has yet to be established, with reported treatment schedules following asthma dosing guidelines ranging from 6 months to 24 months either bi-monthly or monthly [1–5]. These prolonged schedules may both inconvenience patients and increase unnecessary healthcare costs. In this case report, we describe the first case of VKC to be successfully treated with a single 300 mg dose of omalizumab.

**Case presentation:** A 54-year-old male with history of seasonal allergic rhinoconjunctivitis with multiple aeroallergen pollen including grass allergies presented with severe ocular allergies. In the peak of grass season, the patient stated a bilateral gritty eye sensation on a daily basis accompanied with conjunctivitis symptoms several times a day. A diagnosis of VKC was made. Despite conventional treatment, symptoms persisted. A decision was made to administer a single 300 mg dose of subcutaneous omalizumab. Following treatment with omalizumab, ocular symptoms improved completely within a week.

**Conclusions:** We present the first case to our knowledge of VKC to be successfully treated only using a single 300 mg subcutaneous dose of omalizumab. Previous cases have seen treatment of VKC with omalizumab to be effective, but on a longer schedule based on asthma omalizumab dosing guidelines. As such, patients with VKC may respond favorably and completely to only a single dose of omalizumab on a much shorter time frame than would be conventionally used to treat asthma if the problem is mainly driven in a single allergy season.

**Statement of consent:** Consent to publish was obtained from the patient involved in this study.

## ALLIED HEALTH

### A96 Outcomes of teenagers with high-risk asthma followed at the Children’s Allergy & Asthma Education Centre (CAAEC)

#### Jo-Anne St-Vincent^1^, Diane Marks^1^, Beverley Kulbaba^1^, Paria Mostafai Rad^1^, Nancy Ross^1^, Shauna Filuk^1^, Tamar Rubin^1^, Allan Becker^1,2^, Elinor Simons^1,2^

##### ^1^Children’s Allergy & Asthma Education Centre, Health Sciences Centre, Winnipeg, Manitoba, Canada; ^2^Section of Allergy and Clinical Immunology and Children’s Hospital Research Institute of Manitoba, Department of Pediatrics and Child Health, University of Manitoba, Winnipeg, Manitoba, Canada

**Correspondence:** Jo-Anne St-Vincent and Diane Marks

Jo-Anne St-Vincent and Diane Marks Co-first authors

*Allergy, Asthma and Clinical Immunology* 2019, **15(Suppl 1):**A96

**Background:** In 2006, the Children’s Allergy and Asthma Education Centre (CAAEC) began following children at higher risk of serious asthma exacerbations. We worked with their families to improve asthma control, decrease risk of serious asthma-related events and optimize long-term health outcomes and lung function. Criteria for inclusion on the High-Risk Asthma List were: history of PICU admission, frequent Emergency Department visits or hospitalizations, challenging social situations, poor treatment adherence and multiple environmental or food allergies.

**Methods:** We explored the characteristics of teenagers over age 15 years, including their history of other allergic conditions, reasons for inclusion on the High-Risk Asthma List and asthma outcomes at ages 15–17 years.

**Results:** Many of the 50 teenagers with High-Risk Asthma had aeroallergen sensitization (88%), allergic rhinitis (74%), eczema (38%) or food allergies (42%); 34% had a history of PICU admission, 6% had been intubated, and 80% had social concerns. Between ages 15–17 years, 40% had Emergency visits for asthma, 14% had at least 3 Emergency visits, 20% had oral steroids dispensed and 26% had a short-acting bronchodilator dispensed more than twice per year. Dispensed asthma controller medications included inhaled corticosteroids [ICS] (24%), ICS-long-acting bronchodilator combinations (64%), montelukast (20%) and omalizumab (4%). Most recent spirometry showed a median FEV1 of 88% predicted (range 44–122%); 8% had FEV1 < 70% predicted, 2% had FEV1 < 50% predicted, 14% had reversible airflow obstruction and 18% had fixed airflow obstruction.

**Conclusions:** Many CAAEC teenagers with High-Risk Asthma continue to have serious asthma-related events and most continue to require controller medication. The high prevalence of risk factors, exacerbations, airflow obstruction and social concerns emphasizes the importance of addressing medical and social challenges. These findings will inform strategies to prepare teenagers with High-Risk Asthma to maximize their self-management and self-advocacy as they become fully responsible for their health care.

### A97 Epinephrine auto-injectors dispensed to teenagers with high-risk asthma and food allergy while followed by the Children’s Allergy & Asthma Education Centre (CAAEC)

#### Beverley Kulbaba^1^, JoAnne St-Vincent^1^, Diane Marks^1^, Paria Mostafai Rad^1^, Nancy Ross^1^, Shauna Filuk^1^, Tamar Rubin^1^, Allan Becker^1,2^, Elinor Simons^1,2^

##### ^1^Children’s Allergy & Asthma Education Centre, Health Sciences Centre, Winnipeg, Manitoba, Canada; ^2^Section of Allergy and Clinical Immunology and Children’s Hospital Research Institute of Manitoba, Department of Pediatrics and Child Health, University of Manitoba, Winnipeg, Manitoba, Canada

**Correspondence:** Beverley Kulbaba

*Allergy, Asthma and Clinical Immunology* 2019, **15(Suppl 1):**A97

**Background:** The Children’s Allergy and Asthma Education Centre (CAAEC) follows children at high risk for serious asthma exacerbations to improve preventative asthma management. Concomitant food allergy and anaphylaxis risk has become increasingly prevalent among these children. High-risk asthma increases their risk of severe anaphylaxis. We monitored epinephrine auto-injector (EAI) dispensing and carrying among children followed until their teens.

**Methods:** Among teenagers aged 15–17 years on the High-Risk Asthma List, we explored lifetime history of food allergy, persistence of food allergy, and EAI dispensing after availability of the Manitoba provincial Drug Program Information Network (DPIN) in 2011. We examined the pattern of EAI dispensing before and after age 17 years.

**Results:** Of the 50 teenagers followed on the High-Risk Asthma List until age 15–17 years, 21 (42%) had a lifetime history of food allergy and anaphylaxis and had been prescribed an EAI. All remained allergic to at least one food at age 15–17 years, most commonly peanut (81%), cashew (48%) and boney fish (33%). 90% had received an EAI for anaphylaxis, 71% had at least one EAI dispensed from 15 to 17 years, 24% had received EAI samples from the CAAEC, and 76% had their EAI with them at their last visit. Only 57% continued to have an EAI dispensed after age 17 years.

**Conclusions:** Of teenagers followed at the CAAEC for high-risk asthma until their mid-teens, almost half had persistent food allergies and a history of treatment with an EAI at least once for anaphylaxis. Only half continued to carry an EAI after age 17 years. These teenagers are at great risk of life-threatening symptoms from high-risk asthma and anaphylaxis and are at even greater risk because of both conditions. We will implement a graduation CAAEC education session for these teenagers before they transition to self-management.

### A98

Abstract withdrawn

### A99

Abstract withdrawn

### A100 Real world data of Canadians living with hereditary angioedema: part 3-treatment utilization

#### Anne Rowe^1^, Jacquie Badiou^1^, Linda Howlett^1^, Kim Steele^1^, Jenna Falbo^2^, Stephanie Santucci^2^, Jodi Valois^2^, William H. Yang^2,3^

##### ^1^HAE Canada, Ottawa, Ontario, Canada; ^2^Ottawa Allergy Research, Ottawa, Ontario, Canada; ^3^University of Ottawa Medical School, Ottawa, Ontario, Canada

**Correspondence:** Anne Rowe

*Allergy, Asthma and Clinical Immunology* 2019, **15(Suppl 1):**A100

**Background:** Hereditary Angioedema (HAE) is a complex debilitating disease that is often misdiagnosed and under treated. Our study objective was to gain insight into which treatments Canadians are using to treat their acute attacks, prophylactic treatments, and their frequency of administration.

**Methods:** In 2017–2018, data was collected through voluntary online surveys of children, youth, and adults who live with HAE and their caregivers in Canada. The following data was based solely on adult participants.

**Results:** Our Canadian participants are using the following to treat attacks: C1 esterase Inhibitor (Berinert)-61%, C1 esterase Inhibitor (Cinryze)-3%, Icatibant (Firazyr)-10%, Androgen (Danazol)-5%, Tranexamic acid-3%, No pharmaceutical treatment-3%, and Other-15%. Berinert is being used by patients: On demand (36%), Chronic Prophylaxis only (9%), Chronic Prophylaxis and on demand (55%). The majority of respondents use Berinert weekly or twice weekly. Cinryze is being administered as: Chronic Prophylaxis only (33%), and for both Chronic Prophylaxis and on demand (67%). All respondents equally treat at different intervals. Firazyr is being administered as: On demand (89%), and for both Chronic Prophylaxis and on demand (11%). Half of the participants require only one treatment for an acute attack and rarely use it for prophylaxis. Danazol is used for: On demand (25%), Chronic Prophylaxis only (25%), Chronic Prophylaxis and on demand (50%). All respondents equally treat at different intervals. Tranexamic acid is being taken for Chronic Prophylaxis and on demand (100%) by all respondents.

**Conclusion:** The data collected demonstrates that treatments are being used interchangeably for acute and prophylaxis treatment despite the indications listed on the product monographs. These results validate that patients in consultation with their HAE specialists have determined an individualized treatment schedule that works best to control the symptoms of their disease. All results are limited to the respondents and may not represent the broader Canadian HAE population.

### A101 Real world data of Canadians living with hereditary angioedema: part 4-treatment satisfaction

#### Kristy Brosz^1^, Jacquie Badiou^1^, Linda Howlett^1^, Anne Rowe^1^, Kim Steele^1^, Jenna Falbo^2^, Stephanie Santucci^2^, Jodi Valois^2^, William H. Yang^2,3^

##### ^1^HAE Canada, Ottawa, Ontario, Canada; ^2^Ottawa Allergy Research, Ottawa, Ontario, Canada; ^3^University of Ottawa Medical School, Ottawa, Ontario, Canada

**Correspondence:** Kristy Brosz

*Allergy, Asthma and Clinical Immunology* 2019, **15(Suppl 1):**A101

**Background:** Hereditary angioedema (HAE) is a chronic spontaneous life-threatening disease. Due to the unpredictable nature associated with the disease it can have a significant impact on a patient’s quality of life. We sought to better understand the overall satisfaction of treatments from a patient perspective.

**Methods:** In 2017–2018, data was collected through voluntary online surveys of children, youth, and adults who live with HAE and their caregivers in Canada. The following data was based solely on adult participants.

**Results:** Once a proper diagnosis was obtained following patient navigation and treatments were established the annual number of days missed from work or school decreased by an average of 48%, the amount of phone calls to doctor’s offices decreased 60%, the occurrence of unscheduled visits to health care professionals decreased 75%, the frequency of emergency room visits decreased 50%, and the number of hospitalizations decreased 67%. Most patients reported they were satisfied with the frequency they must use their HAE treatments (31%) and satisfied with the effectiveness of their current treatments to prevent attacks (40%). Overall patients were satisfied (39%) and very satisfied (24%) with their current HAE treatment.

**Conclusion:** Results show patients are generally satisfied with the treatments they are currently receiving; however, there are still necessary improvements that will enhance a patient’s quality of life. All results are limited to the respondents and may not represent the broader Canadian HAE population.

### A102 Impact of food allergy on school-age students: perceptions of Winnipeg parents of children with and without food allergy

#### Nancy Ross^1^, Shauna Filuk^1^, Bev Kulbaba^1^, JoAnne St. Vincent^1^, Elinor Simons^1,2^

##### ^1^Children’s Allergy & Asthma Education Centre, Health Sciences Centre, Winnipeg, Manitoba, Canada; ^2^Section of Allergy and Clinical Immunology and Children’s Hospital Research Institute of Manitoba, Department of Pediatrics and Child Health, University of Manitoba, Winnipeg, Manitoba, Canada

**Correspondence:** Nancy Ross

*Allergy, Asthma and Clinical Immunology* 2019, **15(Suppl 1):**A102

**Background:** The entire school community contributes to the safety of school-age children with food allergy. We sought to determine the food allergy perceptions and education needs of parents of children with and without food allergy, with the goal of enhancing the food-allergy education of all parents of school-age children.

**Methods:** The University of Manitoba Health Research Ethics Board and participating school divisions approved the study. Elementary school principals emailed the Invitation Letter and SurveyMonkey^®^ Links to their parent/caregiver contact list. We compared responses of parents of children with and without food allergy using Chi squared tests.

**Results:** Participants included 107 parents of children with food allergy and 454 parents of children without food allergy. Parents of school-age children (ages 7–12 years) with and without food allergy believed that banning foods from class kept allergic students safe (63.8% vs. 65.1%, respectively, p = 0.83), considered food allergy when sending food to school (97.7% vs. 95.7%, p = 0.39), and thought that having a child with food allergy in the classroom taught responsibility (21.5% vs. 22.0%, p = 0.90). Although more parents of children with food allergy thought that greater information and awareness about food allergy was needed (73.9% vs. 44.4%, p < 0.0001), > 80% of both groups thought that parents of children without food allergy required more education and thought that it would be helpful to have a Food Allergy Educator speak at the school.

**Conclusions:** Parents of school-age children with and without food allergy agreed regarding aspects of food allergy management that contributed to safety of children with food allergy. Our findings reflect that parents of children without food allergy are interested in and recognize their need for more education and awareness regarding food allergy in schools. Food allergy education is necessary for the entire school community and should include parents of school-aged children without food allergy.

**Acknowledgments:** We thank all parents who completed the surveys and The Canadian Allergy, Asthma and Immunology Foundation (CAAIF) for funding this study.

### A103 Development of a food allergy app for children and teens at risk for food-induced anaphylaxis

#### Shauna Filuk^1^, Nancy Ross^1^, Saiful Huq^2^, Bev Kulbaba^1^, Jo-Anne St. Vincent^1^, Elinor Simons^1,2^, Tamar Rubin^1,2^, Allan Becker^1,2^

##### ^1^Children’s Allergy & Asthma Education Centre, Health Sciences Centre, Winnipeg, Manitoba, Canada; ^2^Section of Allergy and Clinical Immunology and Children’s Hospital Research Institute of Manitoba, Department of Pediatrics and Child Health, University of Manitoba, Winnipeg, Manitoba, Canada

**Correspondence:** Shauna Filuk

*Allergy, Asthma and Clinical Immunology* 2019, **15(Suppl 1):**A103

**Background:** Adolescents with food allergy are at particular risk for life threatening anaphylaxis. The Children’s Allergy & Asthma Education Centre (CAAEC) has developed both asthma and food allergy education resources and programs for families, children and teens. To develop a food allergy education program for adolescents we undertook a literature review, focus groups and an online survey.

**Methods:** We conducted a literature review, focus groups (n = 16, ages 12–19) and a national survey for food allergic teens (n = 104). This showed Teens were interested in small group interactive education and mobile based learning. We developed a small group interactive program, the Allergy Lounge, and engaged Tactica Interactive for preliminary planning of an app for teens to improve knowledge and self-management skills. The concept for Kung Food emerged and evolved around wireframe screens. CAAEC screened food allergy trivia questions from Food Allergy Canada’s Youth Advisory Panel (FAC/YAP).

**Results:** Using wire frames, youth provided feedback (n = 23). The interactive trivia and scenarios were seen positively. The Kung Food character appealed to a younger age group and interaction and gamification were added. Collaboration with FAC/YAP provided trivia and links for the app’s Allergy Guide. AllerGen funded app development. The Kung Food app is now designed for ages 10–15 years. User testing is underway. A working app will be available in the fall 2018.

**Conclusion:** Interactive smartphone applications are a preferred method for youth to interact. However, development takes a considerable amount of time, effort and funding. The original app concept evolved to include gamification through “earning belts” and the target age shifted to 10–15 years. Further evaluation will assess if Kung Food is an effective tool to help educate and modify behavior for youth.

**Acknowledgements:** Food Allergy Canada’s Youth Advisory Panel contributed trivia questions and the allergy guide for the app. Funding from the Children’s Hospital Foundation of Manitoba supported multimedia initiatives and lead to the development of Kung Food. Funding for Kung Food was provided by AllerGen as part of a National Food Allergy Strategy.

### A104 Strategies for participant retention in pediatric longitudinal research studies: lessons learned from the Vancouver site of the CHILD study

#### Lauren Muttucomaroe^1^, Tamara Coffin^1^, Linda Warner^1^, Maureen Mooney^1^, Mary Beckingham^1^, Diana Lefebvre^2^, Padmaja Subbarao^3^, Malcolm Sears^4^ and Stuart Turvey^1^

##### ^1^Department of Pediatrics, Faculty of Medicine, The University of British Columbia, BC Children’s Hospital Research Institute, Vancouver, British Columbia, Canada; ^2^Department of Medicine, McMaster University, Hamilton, Ontario, Canada; ^3^Department of Paediatrics, Hospital for Sick Children, University of Toronto, Toronto, Ontario, Canada; ^4^Department of Medicine, Faculty of Respirology, McMaster University, Hamilton, Ontario, Canada

**Correspondence:** Lauren Muttucomaroe

*Allergy, Asthma and Clinical Immunology* 2019, **15(Suppl 1):**A104

**Background:** Birth cohort studies are a powerful research methodology; however, retaining pediatric cohorts for long periods is challenging. The Vancouver site of the Canadian Healthy Infant Longitudinal Development (CHILD) Study implemented strategies to optimize retention.

**Methods:** Multi-faceted strategies stemming from feedback from surveys included personalized approaches for families, and techniques to reduce apprehension from invasive procedures (staff recorded children’s reactions to blood collections using an adapted pain scale). Knowledge translation strategies included newsletters, a storybook and email updates.

**Results:** The Vancouver site of the CHILD study recruited 812 pregnant women and 783 infants remained eligible and were participating at birth. The number of participants competing major assessments were: 749 (95.7%) at 1 year, 730 (93.2%) at 3 years, and 728 (93.0%) at 5 years. The main reasons provided for withdrawals included: (i) lacks time required;(ii) lost to follow-up; and (iii) moved away from study site. A voluntary survey was completed by 405 participants: 92% reported that their child had an *‘overall positive experience’*; 90% indicated that they would *‘consider taking part in future studies based on their experience’*; 80% acknowledged a reduction in discomfort during the blood draw with topical anaesthetic. 62% reported *‘improving child health globally’* as their top reason for staying in the study. Invasive procedures like phlebotomy can be a barrier to participation in pediatric studies. At the Vancouver site, 548 (75.3%) participants completed the blood draw at age 5-years. Participants were monitored for pain and 84% of participants reported scores ranging between zero and two (0 = “No pain”, 2 = “A little bit of pain”).

**Conclusions:** By employing a family-focused, multifaceted retention approach, the Vancouver site achieved retention rates above the published average of 80–89% [1–3]. Moving forward, our site is looking to achieve optimal retention through strategies like focus groups, participant events and the creation of a Parent Advisory Council.

